# Burden of non-CO poisoning in 204 countries and territories, 1990–2021: results from the global burden of disease study 2021

**DOI:** 10.3389/fpubh.2025.1620523

**Published:** 2025-07-25

**Authors:** Rong Lei, Chaofu Yue, Feng Yue, Hong Gao, Xing He, Qinyong Yan, Zhigang Yang, Wei Bao, Caimei Hu, Qingsong Ma, Mei Yang

**Affiliations:** ^1^Department of Intensive Care Unit, Yunnan Qujing Central Hospital (Qujing No.1 People’s Hospital), Qujing, China; ^2^Department of Emergency, Yunnan Qujing Central Hospital (Qujing No.1 People’s Hospital), Qujing, China

**Keywords:** non-carbon monoxide poisoning, global disease burden, disability-adjusted life years, prevalence, mortality, socio-demographic index, vulnerable populations, public health strategies

## Abstract

**Background:**

Non-carbon monoxide (non-CO) poisoning remains a significant global public health issue, contributing to considerable morbidity and mortality. However, there is a lack of comprehensive analysis regarding the global burden and trends of non-CO poisoning.

**Methods:**

Data from the Global Burden of Disease (GBD) 2021 study were used to assess the global, regional, and national prevalence, mortality, and disability-adjusted life years (DALYs) associated with non-CO poisoning from 1990 to 2021. Descriptive statistical methods were applied to assess global, regional, and national trends in non-CO poisoning burden, with estimates of prevalence, mortality, and DALYs. Smoothing splines models were used to examine the relationship between non-CO poisoning burden and the Socio-Demographic Index (SDI).

**Results:**

In 2021, global non-CO poisoning accounted for 3.58 million prevalent cases (ASPR: 43.34/100,000), 27.26 thousand deaths (ASDR: 0.35/100,000), and 1.65 million DALYs (ASR: 21.72/100,000), with respective declines of 43.9, 38.9, and 43.5% since 1990. The burden of non-CO poisoning varies significantly across countries and regions, overall, the burden of non-CO poisoning shows a negative correlation with the SDI, with regions and countries with lower SDI values experiencing higher rates of poisoning-related harm. Children under 5 years old and the older adult bear a higher disease burden, with males generally experiencing higher disease burden than females.

**Conclusion:**

Although the global burden of non-CO poisoning has decreased, low- and middle-income countries (LMICs), especially those with lower SDI, continue to experience a disproportionately high burden. Future research should focus on agent-specific epidemiology, improving data collection in LMICs, and examining the impact of agricultural and environmental exposures. Targeted interventions for vulnerable populations, such as children and the older adult, as well as the integration of mental health considerations into prevention strategies, are essential for reducing the global burden.

## Introduction

Poisoning is a significant global public health issue, contributing to substantial morbidity and mortality across all age groups. It is defined as the unintentional exposure to a non-infectious substance through inhalation, ingestion, injection, or absorption, leading to physiological dysfunction or death (1, 2). According to the Global Burden of Disease (GBD) cause classification, poisoning is categorized into carbon monoxide (CO) poisoning and non-carbon monoxide (non-CO) poisoning. CO poisoning has been extensively studied, with regulatory measures such as ventilation improvements, mandatory CO alarms, and public health campaigns effectively reducing CO-related mortality (3, 4). In contrast, non-CO poisoning remains less systematically investigated despite its substantial burden and complex etiology.

Non-CO poisoning results from exposure to industrial chemicals, pesticides, pharmaceuticals, household toxins, heavy metals, solvents, agrochemicals, and other toxic substances, each with varying toxicokinetics and health outcomes (5–7). Despite progress in reducing the burden of CO poisoning, non-CO poisoning continues to pose a significant health threat, particularly in low- and middle-income countries (LMICs) (6, 8, 9). In India, pesticide poisoning remains one of the leading causes of poisoning-related mortality, disproportionately affecting rural agricultural communities due to limited healthcare access and regulatory oversight (10). In contrast, in high-income countries, non-CO poisoning patterns are more commonly associated with pharmaceutical overdoses and chronic exposure to environmental contaminants (11–13). For instance, in Europe, cadmium exposure has been strongly linked to chronic kidney disease and osteoporosis, with Poland and Czechia reporting the highest disease burden (14). Furthermore, exposure to perfluorooctanoic acid in Spain has been associated with an increased prevalence of hypertension. In Australia, pharmaceutical opioid overdoses have surpassed heroin-related mortality, with deaths increasing from 21.9 to 36.2 per million population between 2001 and 2012 (15). While regulatory measures and medical advancements have mitigated poisoning-related burdens in some regions, substantial disparities persist. Certain areas continue to experience high poisoning rates due to occupational exposures, inadequate chemical safety measures, and limited access to emergency toxicological care (9, 14, 16). Existing research on non-CO poisoning primarily focused on specific countries or regions, resulting in fragmented and localized data, while a comprehensive global assessment remains lacking.

This study aims to conduct a comprehensive assessment of non-CO poisoning burden across 204 countries and territories from 1990 to 2021, utilizing GBD 2021 data. The analysis will explore temporal trends, regional differences, and the relationship between poisoning burden and the Socio-Demographic Index (SDI) to generate epidemiological evidence for targeted policy interventions and improved global regulatory measures (17, 18). A comprehensive understanding of these patterns is crucial for guiding public health strategies to enhance poisoning surveillance, improve emergency response systems, and strengthen regulatory control over hazardous substances.

## Methods

### Study design and data source

This study is based on the GBD 2021 estimates, which provide standardized epidemiological assessments across 204 countries and territories from 1990 to 2021. The GBD database, maintained by the Institute for Health Metrics and Evaluation (IHME), systematically compiles data from multiple sources, including vital registration systems, hospital records, household surveys, and disease registries, to generate globally comparable estimates of disease burden (19). Non-CO poisoning mortality and population data were obtained from the GBD 2021 database, stratified by age, sex, year, country, and region. These data are publicly accessible via IHME’s web-based tools,^1^ ensuring transparency and reproducibility. All datasets are freely available through the Global Health Data Exchange,^2^ with detailed documentation on data sources, methodologies, and statistical modeling in previous reports (20, 21).

### Case definition and classification

In the GBD cause classification, poisonings are divided into Poisoning by carbon monoxide and Poisoning by other means. For clarity and consistency in analysis, we refer to ‘Poisoning by other means’ as non-CO poisoning, as defined in the GBD classification. Cases were identified based on International Classification of Diseases (ICD-10 and ICD-9) codes, ensuring consistency across reporting periods.

### Statistical analysis and visualization

Descriptive statistical methods were applied to analyze temporal trends in the burden of non-CO poisoning from 1990 to 2021, with estimates of prevalence, mortality, and disability-adjusted life years (DALYs) stratified by age, sex, country, and region (22). DALYs were computed as the sum of years of life lost (YLLs) due to premature mortality and years lived with disability (YLDs), following the GBD standard life table, with YLLs estimated by multiplying the number of deaths in each age group by the remaining life expectancy at that age. The association between non-CO poisoning burden (DALYs) and the Socio-Demographic Index (SDI) was assessed using Smoothing Splines models (23). Data visualization was conducted using R software (version 4.3.3), and final figure refinements were performed in Adobe Illustrator (CS5) to ensure clarity and precision. Detailed methodological principles and computational frameworks are available in previous studies (21).

## Results

### Global level

In 2021, there were an estimated 3.58 million (95% uncertainty interval [UI]: 3.05–4.45) prevalent cases of non-CO poisoning globally. The age-standardized prevalence rate (ASPR) was 43.34 per 100,000 population, representing a 43.9% decline compared to 1990. A total of 27,260 deaths (95% UI: 16,621–33,237) were attributed to non-CO poisoning, with an age-standardized death rate (ASDR) of 0.35 per 100,000, reflecting a 38.9% reduction since 1990. Non-CO poisoning accounted for 1.65 million DALYs (95% UI: 1.14–2.06), with an age-standardized disability-adjusted life year (DALY) rate of 21.72 per 100,000 population, indicating a 43.5% decrease from 1990 (Table 1).

**Table 1:** Prevalent cases, deaths, and disability-adjusted life years (DALYs) for non-carbon monoxide poisoning in 2021, and percentage change in age-standardized rate (ASR) per 100,000, by Global Burden of Disease region, from 1990 to 2021.

	Prevalence Number in millions (95%UI)	ASPR per 100,000 (95%UI)	Percentage change in ASPR from 1990 to 2021	Death Number in thousands (95% UI)	ASDR per 100,000 (95% UI)	Percentage change in ASDR from 1990 to 2021	DALYs Number in thousands (95% UI)	ASR of DALY per 100,000 (95% UI)	Percentage change in ASR of DALY from 1990 to 2021
Global	3.583 (3.046, 4.448)	43.337 (36.84, 53.724)	−43.904 (−44.561, −43.146)	27.263 (16.621, 33.237)	0.345 (0.211, 0.425)	−38.874 (−62.709, −20.483)	1652.096 (1139.789, 2057.575)	21.724 (14.935, 27.503)	−43.461 (−61.753, −24.279)
High-income Asia Pacific	0.297 (0.249, 0.373)	127.008 (105.972, 160.837)	−34.583 (−36.066, −32.875)	0.165 (0.138, 0.245)	0.042 (0.035, 0.068)	−86.624 (−89.138, −77.9)	23.331 (16.709, 31.041)	10.933 (7.546, 14.826)	−59.088 (−65.742, −51.615)
High-income North America	0.655 (0.549, 0.796)	147.188 (122.955, 181.094)	−31.46 (−33.761, −29.437)	0.205 (0.195, 0.213)	0.049 (0.047, 0.05)	−75.147 (−76.202, −74.121)	45.757 (34.406, 59.289)	11.704 (8.763, 15.204)	−51.865 (−55.342, −49.103)
Western Europe	0.641 (0.538, 0.787)	126.926 (105.462, 156.654)	−23.575 (−24.909, −22.449)	0.13 (0.121, 0.137)	0.02 (0.019, 0.021)	−88.652 (−89.087, −88.148)	53.306 (36.427, 72.585)	11.561 (7.855, 15.952)	−48.429 (−55.341, −42.721)
Australasia	0.038 (0.031, 0.049)	113.875 (92.68, 146.03)	−13.988 (−16.378, −10.548)	0.01 (0.009, 0.011)	0.024 (0.022, 0.027)	−79.926 (−81.956, −77.781)	3.637 (2.496, 5.062)	11.346 (7.717, 15.999)	−36.378 (−44.183, −28.749)
Andean Latin America	0.04 (0.033, 0.051)	60.331 (50.463, 76.233)	−38.925 (−40.967, −36.3)	0.205 (0.161, 0.282)	0.314 (0.247, 0.43)	−75.546 (−81.228, −65.068)	14.261 (11.599, 18.825)	21.423 (17.381, 28.246)	−74.421 (−79.507, −65.634)
Tropical Latin America	0.069 (0.058, 0.085)	28.274 (24.05, 35.065)	−37.65 (−39.047, −35.798)	0.087 (0.082, 0.093)	0.037 (0.034, 0.039)	−75.025 (−76.849, −72.663)	9.913 (7.922, 12.048)	4.255 (3.419, 5.176)	−66.261 (−69.938, −62.413)
Central Latin America	0.251 (0.207, 0.324)	96.659 (79.792, 124.77)	−32.955 (−34.079, −31.547)	0.554 (0.48, 0.635)	0.216 (0.187, 0.248)	−70.776 (−74.756, −66.667)	52.301 (43.872, 63.442)	20.309 (17.013, 24.651)	−62.842 (−66.792, −59.006)
Southern Latin America	0.194 (0.16, 0.249)	269.706 (222.741, 349.398)	−20.088 (−25.427, −17.638)	0.037 (0.031, 0.043)	0.049 (0.041, 0.058)	−91.659 (−93.091, −90.001)	18.087 (12.2, 24.886)	25.994 (17.522, 35.747)	−56.848 (−64.9, −49.653)
Caribbean	0.038 (0.032, 0.048)	76.566 (63.68, 96.518)	−18.657 (−20.974, −16.495)	0.099 (0.072, 0.158)	0.219 (0.158, 0.352)	−76.886 (−83.864, −60.266)	9.687 (7.327, 14.011)	21.603 (16.037, 32.133)	−68.443 (−77.674, −50.491)
Central Europe	0.178 (0.141, 0.227)	137.968 (108.767, 178.383)	−43.888 (−45.953, −41.898)	0.07 (0.055, 0.11)	0.041 (0.033, 0.065)	−94.564 (−95.741, −91.32)	18.188 (12.52, 25.452)	14.701 (10.035, 20.841)	−75.236 (−80.413, −69.832)
Eastern Europe	0.159 (0.128, 0.22)	66.526 (52.886, 91.727)	−43.164 (−46.785, −39.993)	0.082 (0.075, 0.089)	0.035 (0.032, 0.037)	−59.467 (−71.164, −48.211)	15.859 (11.87, 20.554)	7.208 (5.431, 9.341)	−53.271 (−58.169, −48.711)
Central Asia	0.072 (0.056, 0.093)	75.322 (59.129, 97.96)	−33.824 (−35.96, −31.549)	0.041 (0.033, 0.053)	0.044 (0.036, 0.057)	−78.301 (−83.775, −70.624)	8.64 (6.483, 11.16)	8.99 (6.75, 11.626)	−58.594 (−65.109, −52.978)
North Africa and Middle East	0.092 (0.076, 0.115)	15.098 (12.471, 18.788)	−47.9 (−49.536, −46.219)	1.299 (0.691, 1.827)	0.217 (0.116, 0.304)	−69.092 (−76.616, −57.967)	87.78 (50.337, 122.186)	14.005 (8.019, 19.463)	−70.509 (−77.302, −60.356)
South Asia	0.154 (0.127, 0.194)	8.286 (6.886, 10.445)	−40.675 (−42.845, −38.574)	1.95 (1.14, 2.578)	0.115 (0.069, 0.151)	−63.338 (−71.954, −48.567)	119.226 (78.842, 157.547)	6.527 (4.322, 8.63)	−67.348 (−75.139, −53.12)
Southeast Asia	0.136 (0.114, 0.171)	18.701 (15.723, 23.424)	−34.534 (−35.753, −33.465)	2.529 (1.304, 3.216)	0.349 (0.184, 0.441)	−43.748 (−58.317, −20.307)	131.483 (75.355, 163.743)	17.902 (10.336, 22.214)	−44.338 (−56.869, −25.817)
East Asia	0.376 (0.312, 0.454)	20.923 (17.398, 25.628)	−30.922 (−35.666, −26.74)	8.319 (3.274, 11.004)	0.455 (0.177, 0.595)	−7.328 (−78.821, 95.793)	320.52 (143.253, 414.845)	19.959 (8.887, 25.499)	−27.376 (−81.002, 38.982)
Oceania	0.003 (0.002, 0.003)	21.422 (18.118, 26.471)	−12.068 (−14.018, −10.084)	0.05 (0.031, 0.103)	0.37 (0.226, 0.778)	−38.134 (−55.131, −8.908)	3.526 (2.312, 6.796)	23.256 (15.211, 45.652)	−35.992 (−54.191, −5.801)
Western Sub-Saharan Africa	0.08 (0.066, 0.1)	19.467 (16.081, 24.314)	−34.477 (−36.348, −32.551)	4.166 (2.623, 5.736)	1.094 (0.645, 1.484)	−32.285 (−54.955, −6.45)	279.223 (180.797, 383.516)	51.436 (33.281, 70.056)	−36.541 (−59.827, −5.264)
Eastern Sub-Saharan Africa	0.079 (0.065, 0.1)	22.114 (18.49, 27.605)	−43.351 (−45.625, −41.407)	4.803 (3.021, 6.767)	1.686 (1.14, 2.151)	−41.431 (−53.448, −21.431)	294.562 (182.481, 445.139)	71.39 (46.242, 99.64)	−47.752 (−62.248, −22.406)
Central Sub-Saharan Africa	0.018 (0.015, 0.022)	15.524 (13.005, 19.309)	−42.007 (−45.005, −39.339)	1.349 (0.809, 2.218)	1.532 (0.941, 2.261)	−35.039 (−52.153, −9.176)	77.834 (44.733, 144.212)	61.926 (38.091, 99.661)	−44.871 (−61.496, −12.439)
Southern Sub-Saharan Africa	0.013 (0.011, 0.016)	16.249 (13.534, 20.395)	−38.404 (−39.839, −37.01)	1.114 (0.849, 1.362)	1.425 (1.092, 1.736)	−19.053 (−37.218, 1.265)	64.975 (48.472, 80.055)	79.292 (59.134, 97.741)	−20.74 (−36.947, 2.475)

### Regional level

In 2021, there was significant regional variation in the burden of non-CO poisoning. Southern Latin America (269.7 per 100,000), High-income North America (147.2 per 100,000), and Central Europe (137.97 per 100,000) reported the highest ASPR, while regions such as South Asia (8.3 per 100,000), North Africa and the Middle East (15.1 per 100,000), and Central Sub-Saharan Africa (15.5 per 100,000) had the lowest ASPR.

In terms of death rates, Eastern Sub-Saharan Africa (1.686 per 100,000), Central Sub-Saharan Africa (1.532 per 100,000), and Southern Sub-Saharan Africa (1.425 per 100,000) exhibited the highest ASDR from poisoning. In contrast, regions like Western Europe (0.02 per 100,000), Australasia (0.024 per 100,000), and Eastern Europe (0.035 per 100,000) reported the lowest ASDR. The highest Age-standardized rate (ASR) of DALY for poisoning was found in Southern Sub-Saharan Africa (79.292 per 100,000), Eastern Sub-Saharan Africa (71.390 per 100,000), and Central Sub-Saharan Africa (61.926 per 100,000). Conversely, regions such as Tropical Latin America (4.255 per 100,000), South Asia (6.527 per 100,000), and Eastern Europe (7.208 per 100,000) exhibited the lowest ASR of DALY (Table 1).

From 1990 to 2021, significant reductions in the burden of non-CO poisoning were observed across all regions. The largest decreases in ASPR occurred in North Africa and the Middle East (−47.9%), Central Europe (−43.9%), and Eastern Sub-Saharan Africa (−43.4%), while the smallest reductions were seen in Oceania (−12.1%), Australasia (−13.99%), and the Caribbean (−18.7%). In terms of ASDR, the most significant declines were observed in Central Europe (−94.6%), Southern Latin America (−91.7%), and Western Europe (−88.7%), with Southern Sub-Saharan Africa (−19.1%), Western Sub-Saharan Africa (−32.3%), and Central Sub-Saharan Africa (−35.0%) experiencing the smallest reductions. For DALYs, the greatest reductions were found in Central Europe (−75.2%), Andean Latin America (−74.4%), and North Africa and the Middle East (−70.5%), while the smallest reductions were observed in Southern Sub-Saharan Africa (−20.7%), East Asia (−27.4%), and Oceania (−36.0%; Table 1).

### National level

**Figure 1:**
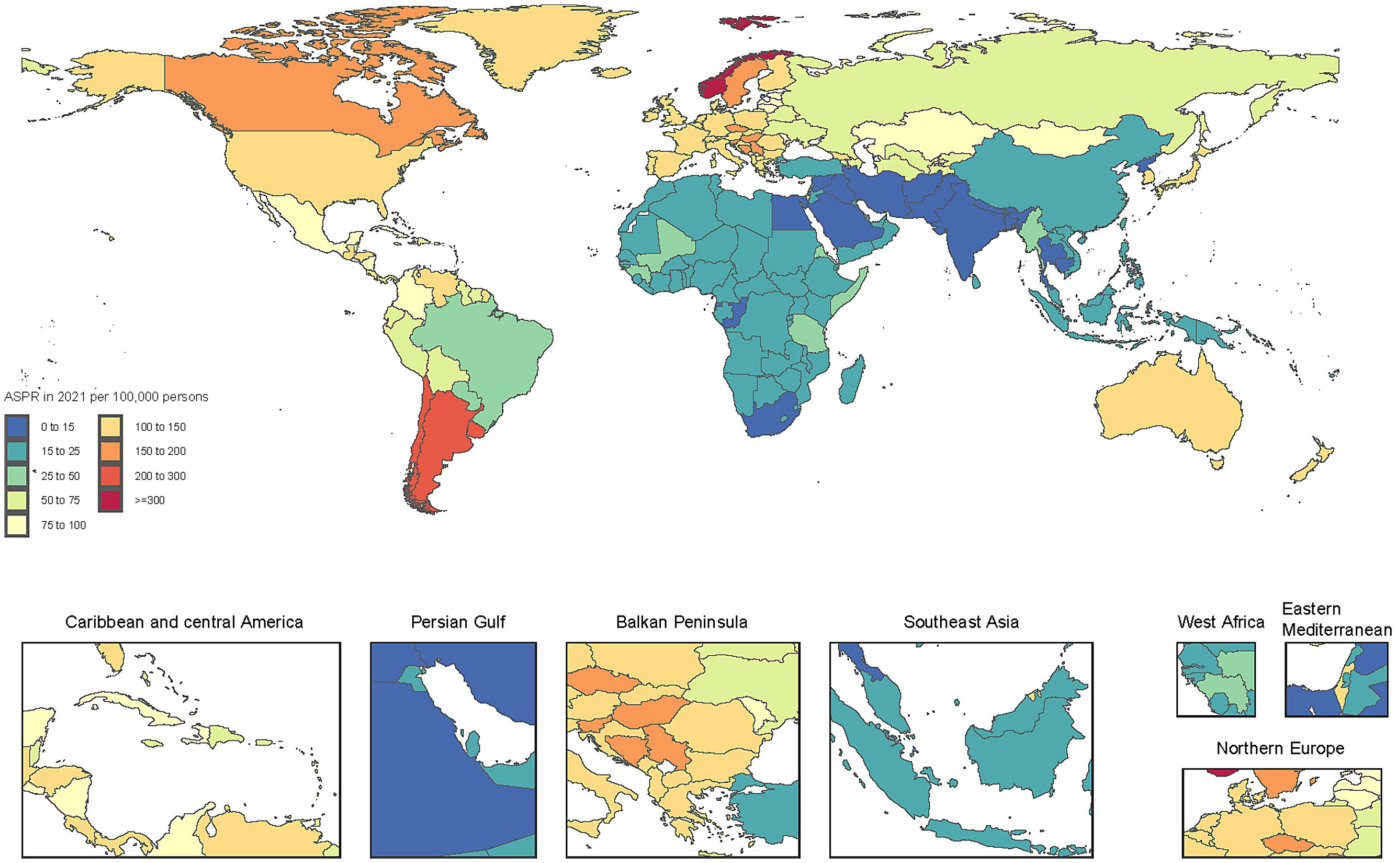
Age-standardized prevalence rate of non-CO poisoning per 100,000 population in 2021, by country.

The burden of non-CO poisoning demonstrated a generally declining trend across most countries from 1990 to 2021, although some regions experienced increases. The highest ASPR was reported in Norway (443.6 per 100,000), Argentina (289.8 per 100,000), and Chile (231.2 per 100,000), whereas the lowest was recorded in Bangladesh (7.5 per 100,000), India (7.9 per 100,000), and Bhutan (8.2 per 100,000; Figure 1; Supplementary Table S1). The highest ASDR were observed in Somalia (3.52 per 100,000), Zimbabwe (2.99 per 100,000), and South Sudan (2.69 per 100,000), while Bermuda (0.00069 per 100,000), Switzerland (0.00095 per 100,000), and Andorra (0.00257 per 100,000) reported the lowest ASDR. Regarding ASR of DALY, the highest were recorded in Jamaica (156.5 per 100,000), Oman (141.4 per 100,000), and Colombia (124.6 per 100,000), whereas Venezuela (1.9 per 100,000), Latvia (2.2 per 100,000), and Malaysia (2.8 per 100,000) had the lowest (Supplementary Table S3).

From 1990 to 2021, trends in the burden of non-CO poisoning varied markedly by country. Norway (+37.2%), Cuba (+25.7%), and the Solomon Islands (+7.1%) experienced the greatest increases in ASPR, while Mongolia (−65.3%), Iran (−64.1%), and Romania (−61.3%) showed the most substantial decreases. The most significant increases in ASDR were found in Cabo Verde (+903.7%), Lesotho (+38.5%), and Chad (+24.0%), in contrast to dramatic reductions in Bermuda (−99.7%), Puerto Rico (−99.5%), and Croatia (−98.6%). Similarly, the steepest rises in ASR of DALY occurred in Bahrain (+221.2%), Ghana (+39.0%), and Iceland (+26.8%), whereas the greatest declines were seen in Namibia (−94.5%), Egypt (−93.0%), and Nicaragua (−92.9%).

### Age and sex patterns

**Figure 2:**
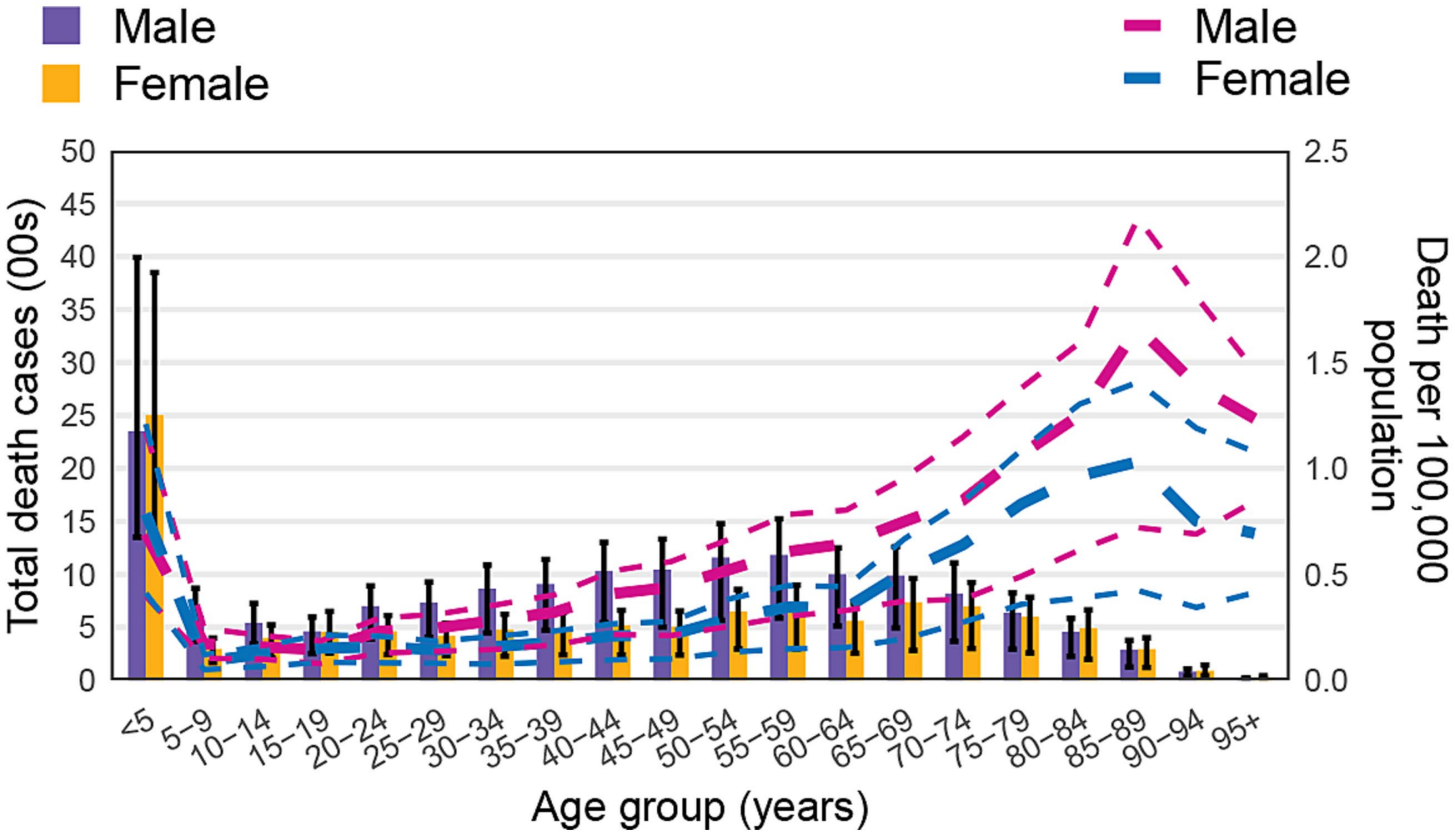
Number of prevalent cases globally and prevalence of non-CO poisoning per 100,000 population, by age and sex in 2021. Lines indicate the prevalence with 95% uncertainty intervals for males and females across different age groups.

In 2021, the global burden of non-CO poisoning exhibited distinct age- and sex-related patterns. The age-specific prevalence increased steadily with age, peaking in the ≥95 years group. In terms of prevalent cases, the highest number was observed in the 35–54 years age group, followed by a gradual decline in older populations. Males had more prevalent cases across all age groups up to 64 years. However, in older age groups (≥65 years), the number of prevalent cases in females gradually exceeded that in males (Figure 2).

**Figure 3:**
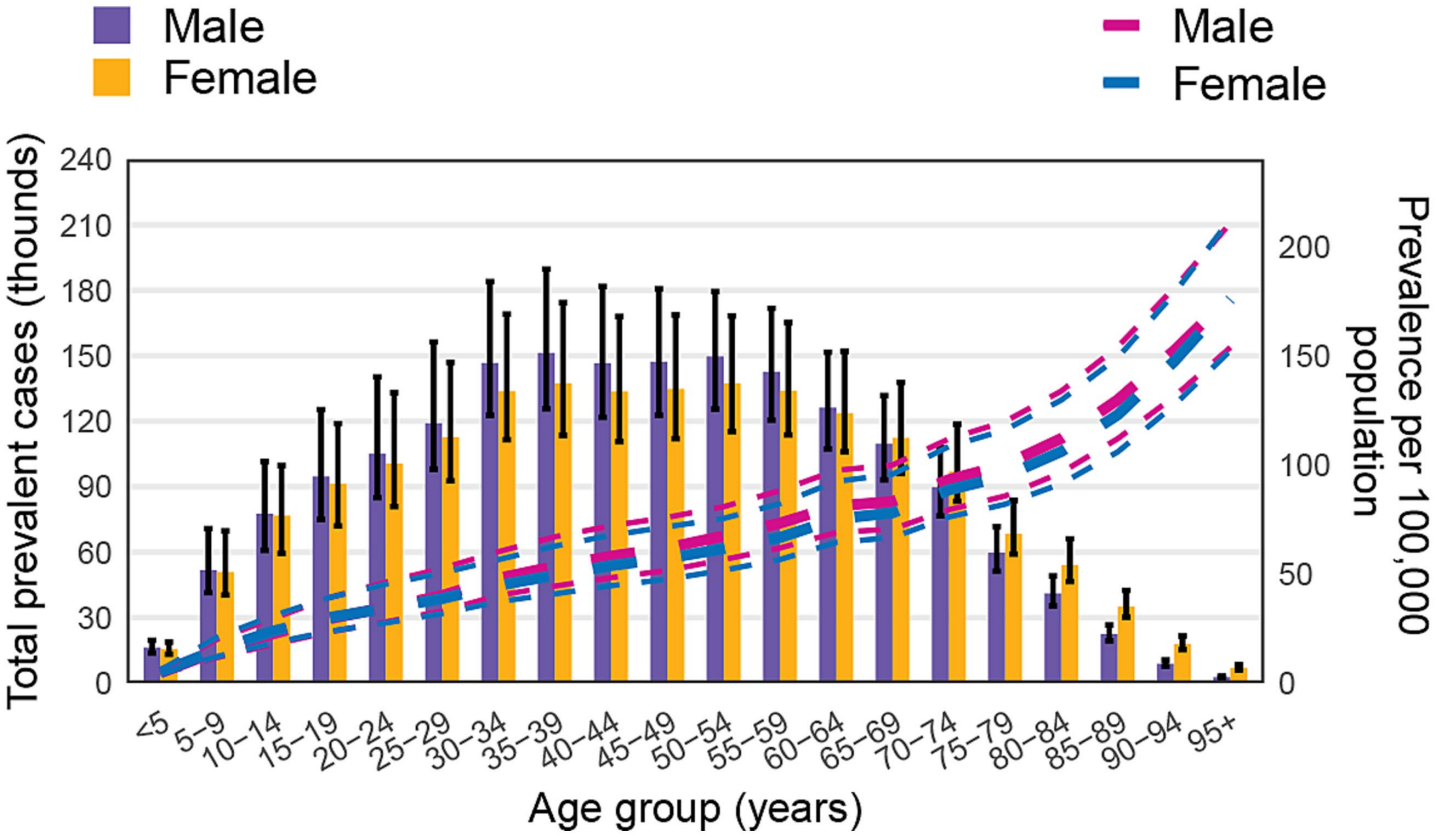
Global number of deaths and death rate of non-CO poisoning per 100,000 population, by age and sex in 2021. Lines show the death rates with 95% uncertainty intervals for males and females across different age groups.

Regarding death rate, the highest number of deaths was observed in the <5 years age group, with both sexes showing elevated rates. Death rate sharply declined in the 5–9 years age group, then progressively increased with age, peaking in the 85–89 years age group. While males experienced higher death rates and numbers in all age groups, female death numbers began to surpass those of males from the 80–84 years age group onward (Figure 3).

**Figure 4:**
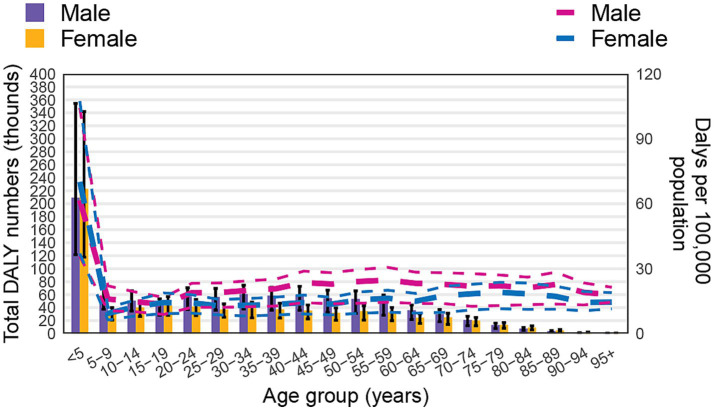
Global number of DALYs and DALY rate of non-CO poisoning per 100,000 population, by age and sex in 2021. Lines represent the DALY rate with 95% uncertainty intervals for males and females across different age groups.

In terms of DALYs, the pattern mirrored that of death. The DALY rate was particularly high in the <5 years age group, reflecting the severe impact of non-CO poisoning in this age bracket. After this, the DALY rate sharply declined in the 5–9 years group, then gradually increased with age, reaching a plateau in older age groups. Regarding DALY numbers, females in the <5 years group observed higher values than males, a trend that reversed as age increased, with males showing higher DALY numbers in most age groups. However, in the 80–84 years group, DALY numbers in females surpassed those in males once again (Figure 4).

### Association with the sociodemographic index

**Figure 5:**
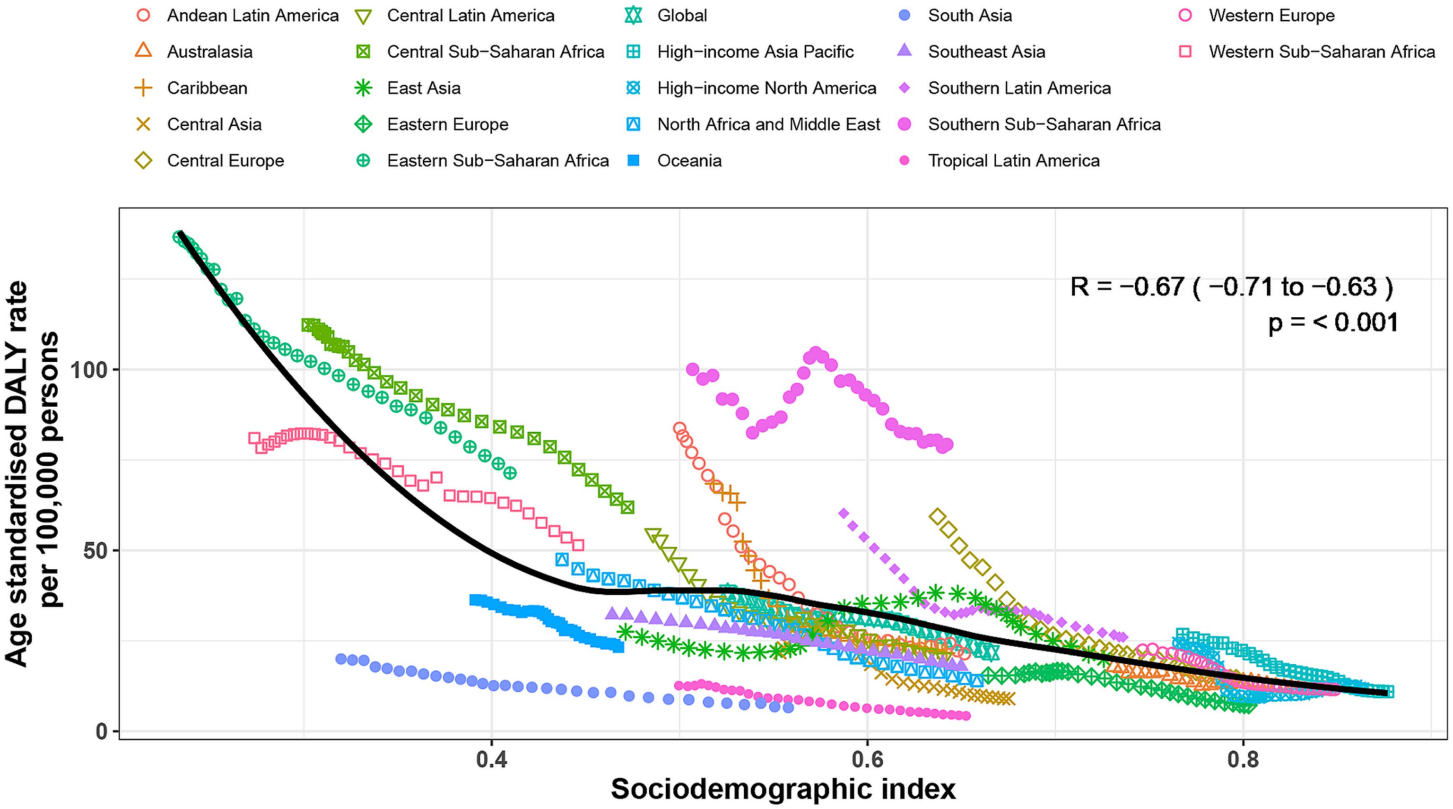
Age-standardized disability-adjusted life year (DALY) rates of non-CO poisoning by Socio-Demographic Index for 21 Global Burden of Disease regions, 1990–2021. Each region is represented by 32 data points corresponding to observed age-standardized DALY rates from 1990 to 2021. The solid line indicates the expected DALY rates based on the SDI and global patterns. Regions above the line represent a higher than expected burden of non-CO poisoning, while those below the line indicate a lower than expected burden.

The burden of non-CO poisoning exhibited a significant negative correlation with the SDI at both regional and national levels. At the global level, regions with lower SDI values demonstrated higher age-standardized DALY rate attributable to non-CO poisoning, while higher-SDI regions showed lower ASR values (Figure 4). At the regional level, the correlation coefficient between SDI and age-standardized DALY rate was −0.67 (95% CI: −0.71 to −0.63, *p* < 0.001; Figure 5). Similarly, at the national level, the correlation coefficient was −0.68 (95% CI: −0.75 to −0.6, *p* < 0.001; Supplementary Figure S1). Countries with lower SDI values had higher ASR of DALYs from non-CO poisoning, while those with higher SDI values exhibited lower ASR.

Although, overall, the burden of non-CO poisoning exhibited a significant negative correlation with SDI, a more nuanced pattern emerged when examining the relationship across the full SDI spectrum. At the regional level, SDI values below 0.45 were associated with a noticeable decrease in age-standardized DALY rate, followed by a slight increase in ASR of DALY between SDI values of 0.45 and 0.52. After this slight increase, ASR began to decrease again. Southern Sub-Saharan Africa had higher than expected ASR, while regions such as South Asia and Tropical Latin America had lower than expected burdens from 1990 to 2019 (Figure 5). At the national level, the burden of non-CO poisoning generally decreased with increasing SDI. However, in countries with an SDI greater than 0.8, a slight upward trend in the burden of non-CO poisoning was observed, as seen in countries like Norway and Monaco. Countries such as Zimbabwe, Lesotho, and Eswatini exhibited much higher than expected burdens, while countries like Bhutan, Pakistan, Qatar, and Switzerland showed much lower than expected ASR.

## Discussion

### Principal findings

This study offers a comprehensive global, regional, and national assessment of the burden of non-CO poisoning from 1990 to 2021, revealing significant reductions in prevalence, mortality, and DALYs. In 2021, there were 3.58 million prevalent cases of non-CO poisoning globally, 27.26 thousand deaths, and 1.65 million DALYs. The ASPR decreased by 43.9%, ASDR decreased by 38.9%, and ASR of DALY decreased by 43.5%. These reductions are a direct result of advancements in poisoning prevention strategies, improved healthcare accessibility, and strengthened regulatory enforcement (24–26). However, the persistence of geographic and socioeconomic disparities remains a significant challenge, particularly in LMICs, where structural factors such as limited access to healthcare, weak regulations, and socioeconomic pressures continue to exacerbate the poisoning burden (27–29).

In 2021, Asia and Africa exhibited relatively lower poisoning prevalence rates compared to other continents. However, it is noteworthy that Africa displayed the highest ASDR and age-standardized DALY rate, indicating that they may face significant challenges in poisoning prevention and emergency response systems (30, 31). In contrast, high-income regions such as North America and Europe reported relatively higher prevalence rates, yet their ASDRs and DALYs were lower than those observed in other regions. This disparity likely reflects the presence of well-established public health systems that are more adept at addressing poisoning incidents and mitigating long-term health impacts (Figure 1) (32–34). A similar trend is observed in the correlation between the Socio-Demographic Index (SDI) and age-standardized rates (ASR) of DALYs, highlighting the relationship between socio-economic development and the burden of poisoning.

A clear negative correlation is observed between the SDI and ASR of DALY, with lower SDI levels corresponding to higher ASR of DALY (Figure 2). For instance, regions such as Asia, Andean Latin America, and Tropical Latin America have demonstrated a decline in both mortality rates and DALYs, which may be attributed to improvements in economic development, healthcare access, and public health infrastructure (35). Interestingly, a slight increase in SDI levels between 0.4 and 0.6, followed by a gradual decline, was observed. This pattern may be linked to the increased use of chemicals and industrial pollution associated with rapid economic development in these regions (36, 37). It underscores the need for concurrent environmental regulation policies as countries advance economically, to prevent the adverse health outcomes associated with industrialization and chemical exposure (38–40).

At the national level, a clear inverse relationship was observed between SDI and the burden of non-CO poisoning (Supplementary Figure S1). Countries with lower SDI values generally experienced higher ASR of DALY, reflecting limited healthcare access, weaker regulatory systems, and inadequate emergency response infrastructure. This pattern is particularly evident in nations such as Zimbabwe, Lesotho, and Eswatini, where the poisoning burden significantly exceeded what would be expected based on SDI alone. Conversely, countries with higher SDI values, such as Switzerland, Qatar, and Monaco, typically reported low ASDRs and DALYs, suggesting that robust healthcare systems, strict environmental and chemical safety regulations, and strong public awareness contribute to effective prevention and management of poisoning incidents (41–43). However, a notable exception is seen in countries with SDI values exceeding 0.8, including Norway, where a slight upward trend in poisoning burden was observed. This may be partially attributed to more comprehensive surveillance and reporting systems, increased exposure to industrial and pharmaceutical chemicals, and rising rates of substance misuse—especially in high-income settings where prescription and recreational drug use is more prevalent (44, 45). In such contexts, the burden may reflect not only exposure risks but also societal and behavioral dimensions, such as mental health conditions and substance dependency (46, 47).

Geographic and cultural factors further mediate the relationship between SDI and poisoning burden. For instance, in South Asian countries like Pakistan, Bhutan, and India, low ASPRs and DALYs were reported despite modest SDI levels. This discrepancy may reflect substantial underreporting due to weak surveillance systems (48). Moreover, in regions where traditional medicine and informal care are commonly utilized, cases of poisoning may bypass formal health systems entirely, thereby distorting the true burden (49, 50). In contrast, Latin American countries such as Argentina and Chile, though categorized as upper-middle SDI countries, exhibited elevated ASPRs. This can be linked to extensive pesticide use in agriculture, occupational exposure among rural populations, and inconsistent enforcement of chemical safety regulations (51). Yet, their ASDRs and DALYs remain moderate, potentially due to improving emergency care and a growing emphasis on occupational health protection (52).

Our study revealed clear age-related disparities in non-CO poisoning burden in 2021, with children under five and older adult individuals exhibiting the highest poisoning burdens. Although children had the lowest prevalence rates of non-CO poisoning, they were disproportionately affected by death and DALYs, particularly those under five. This is due to their exploratory behaviors, accidental ingestion of household chemicals, and heightened vulnerability to poisoning, making them especially at risk (53, 54). In addition to the physiological vulnerability, children often lack the ability to articulate symptoms, delaying diagnosis and treatment (55, 56). Unintentional poisoning is a leading cause of childhood injury and mortality, particularly in LMICs, where regulations for hazardous substances in homes and environments are often insufficient (57, 58). Older adults also face increased risk, likely due to polypharmacy, cognitive decline, and reduced ability to metabolize toxins (59, 60). The rise in pharmaceutical poisoning in high-SDI countries, particularly involving opioids, underscores the need for targeted interventions for older populations, including safe medication practices and geriatric toxicology care (61).

Gender differences in poisoning were evident, with males consistently exhibiting higher ASPR than females (Figure 2). This pattern is often attributed to higher occupational exposure, risk-taking behaviors, and greater involvement in industrial and agricultural work (62, 63). However, in older age groups, female poisoning rates surpass male rates, likely due to increased use of medications and longer life expectancy, which expose women to higher rates of pharmaceutical overdoses and chronic toxic exposures (64, 65).

### Complexity of poisoning types and its implications

The complexity of non-CO poisoning lies not only in the variety of toxic agents involved but also in the diverse mechanisms of harm they induce. Poisoning can result in acute toxicity, long-term health consequences, and psychosocial impacts, each contributing uniquely to the global burden (66, 67). Acute poisoning is often linked to occupational exposure, such as pesticide use in agricultural communities, and household toxins (31, 57). In LMICs, pesticide-related poisoning remains prevalent, mainly due to the widespread use of high-risk chemicals in agriculture. Bans on highly toxic pesticides, such as paraquat in South Korea, Sri Lanka, and Japan, have significantly reduced pesticide-related deaths—for example, Japan saw a 92% decline over three decades (68, 69). Notably, in Sri Lanka, the 2015 restriction of less toxic pesticides was associated with increased mortality, highlighting the need to avoid arbitrary bans while more hazardous chemicals remain available (70). To maximize the effectiveness of such policies, complementary measures—including farmer education, safety training, and social support—are essential to improve protective behaviors and ensure the safe use of pesticides in agricultural communities (71).

In contrast, high-income regions, such as North America and parts of Europe, are experiencing a growing crisis of pharmaceutical poisonings, predominantly driven by opioids (46, 72, 73). In Canada, opioid-related deaths nearly doubled from 3,007 in 2019 to 6,222 in 2021, with years of life lost increasing from 126,115 to 256,336, disproportionately affecting adults aged 20–39, where opioids accounted for ~29% of all deaths in this group by 2021 (74). Similarly, in the United States, more than 800,000 premature deaths have been attributed to opioid overdoses since 2000 (72). These trends are not solely due to prescribing practices but also reflect broader social determinants such as economic instability, despair, and isolation (34).

Misuse of other psychoactive pharmaceuticals is also rising. In Australia, poisoning incidents involving antipsychotics such as quetiapine and olanzapine increased by 30.8 and 32.7%, respectively, from 2015 to 2020, often involving co-ingestion with opioids or benzodiazepines, highlighting patterns of risky polypharmacy (75). In response, various strategies have been proposed and partially implemented, including enhanced prescription monitoring programs, public health education, and improved naloxone distribution. Geospatial modeling in Vancouver showed that strategic placement of naloxone kits could improve access, potentially covering over 50% of opioid poisoning events compared to only 35% with existing distribution sites (76). Broader systemic changes—such as reforming prescribing culture, ensuring treatment access, and addressing social inequities—are essential to reverse current mortality trends (34).

Certain types of foodborne poisoning are highly region-specific, often shaped by local dietary practices and traditional food preparation methods. In China, mushroom poisoning remains a serious public health concern, particularly in provinces such as Guizhou, where most cases occur between June and October, and are concentrated in households among individuals aged 20–59. In 2023 alone, 33 species of poisonous mushrooms were identified across 72 incidents, with ingestion commonly driven by foraging habits and misidentification of wild mushrooms (77). In addition, outbreaks of botulism and bongkrekic acid poisoning linked to homemade fermented foods such as pickled eggs, wet rice noodles, and corn flour jelly have been reported, highlighting the risks associated with traditional food preparation (78). Bongkrekic acid outbreaks, mostly occurring in South and Southwest China, showed a case fatality rate of 29.5%, with 79% of incidents occurring in domestic settings (79). These examples underscore the urgent need for targeted public health interventions, including seasonal risk communication, education on safe food sourcing and preparation, and restriction of high-risk traditional practices, to reduce preventable toxic exposures.

The burden of poisoning is disproportionately high in children, especially those under five, due to their exploratory behavior and increased risk of ingesting household chemicals or medications. In the United States, household cleaning products are the second most common cause of poisoning in children under six, with alkali-based cleaners being particularly dangerous (80). Children often misidentify household chemicals, particularly those with food-like packaging or fruity scents, further increasing the risk of ingestion (81). Unsafe medication storage and inadequate parental awareness also exacerbate this risk (27). Targeted interventions, such as childproofing homes, educating caregivers on safe storage practices, and improving child-safe packaging, are essential for reducing these risks. Additionally, increasing access to poison control centers and regulating household chemicals can significantly help prevent pediatric poisonings (55).

Heavy metal exposure remains a critical public health issue, particularly in industrialized regions. Chronic exposure to metals such as lead, mercury, and arsenic has been linked to severe long-term health consequences, including renal damage, cardiovascular diseases, and neurological disorders (82). The GBD Study 2019 reported that lead exposure in the North Africa and Middle East region resulted in 489.3 DALYs per 100,000 population in 2019, with cardiovascular diseases and chronic kidney diseases accounting for significant portions of the burden (35). In China, mercury exposure, primarily from occupational sources and the use of mercury-containing cosmetics, has led to long-term complications such as nephrotic syndrome and peripheral neuropathy (83). Occupational studies have also demonstrated that lead exposure in workers, such as those in automotive garages, significantly elevates blood lead levels and contributes to chronic health risks like renal and neurological damage (63). Furthermore, mixed heavy metal exposure, including arsenic, cadmium, and nickel, has been positively correlated with early renal impairment, highlighting complex interactions between metals and their cumulative effects on kidney health (84). To mitigate these risks, strengthening environmental monitoring systems and implementing long-term, cost-effective interventions, such as regulatory measures and public health policies focused on early detection and screening in at-risk populations, are essential.

Self-poisoning, often linked to mental health disorders such as depression and substance abuse, remains a significant public health issue, particularly in high-stress environments. Studies show that a substantial proportion of poisoning cases are deliberate self-harm, with depression being a key factor; for example, a study in Australia found that 35.5% of young individuals treated for self-poisoning were diagnosed with depression, and 25.4% were referred for psychiatric care (85). Additionally, psychotropic drugs, including antidepressants and benzodiazepines, were more prevalent in poisoning-related suicides, indicating the association between mental health conditions and self-harm behaviors (86). In the U.S., rising self-poisoning cases among adolescents reflect the broader mental health crisis, with substances like acetaminophen and ibuprofen commonly used in these cases (5). Adolescents with cannabis use disorder are also at increased risk of suicide attempts, especially when co-occurring with depression (87). These findings highlight the need to integrate mental health care into poisoning prevention strategies, with early intervention, mental health support, and reduced stigma being crucial to reducing the risk of suicide and self-harm (88).

### Public health strategies and policy recommendations

#### Enhancing pesticide management and safety training

Governments should strengthen regulations on pesticides and hazardous chemicals, especially in agricultural sectors. Providing farmer education and safety training is essential to ensure safe pesticide use and reduce exposure-related poisoning incidents.

#### Improving emergency response and healthcare accessibility

Investment in the establishment of toxicology centers and emergency response facilities is crucial. Training healthcare professionals to manage poisoning cases effectively will improve outcomes in regions with limited resources.

#### Increasing public awareness and risk communication

Public health campaigns should focus on raising awareness about the risks of pharmaceutical overdose, particularly opioids, and expand access to life-saving interventions such as naloxone.

#### Targeted interventions for high-risk populations

Policies should focus on the protection of vulnerable groups, such as children and the older adult, through measures like childproof packaging for household chemicals and safe medication practices.

#### Strengthening international collaboration with Global Health Organizations

Collaborative efforts with global health organizations, such as the WHO, should promote international standards for chemical safety and poisoning prevention, alongside improving data sharing.

#### Addressing socioeconomic determinants of health

Public health and social policies should address underlying socioeconomic factors such as mental health, poverty, and stress, to reduce self-inflicted poisonings and substance abuse-related poisoning.

## Limitations and future research

This study has several limitations. First, data quality in LMICs is often inadequate, which may result in underreporting of non-CO poisoning cases. Second, the study does not distinguish between specific toxicological agents, limiting the capacity for agent-specific analysis. Third, the etiology of non-CO poisoning may evolve over time, necessitating longitudinal studies to better capture emerging risks. Future research should focus on agent-specific epidemiological patterns, enhancing data collection and reporting in LMICs, investigating the impact of agricultural practices, chemical exposures, and environmental pollutants on poisoning risks, and exploring protective interventions for vulnerable populations, such as children under five and the older adult. Additionally, there is a need to investigate the relationship between mental health and poisoning risks, particularly in the context of self-inflicted poisonings, and to develop targeted preventive measures for this group.

## Conclusion

This study provides a global assessment of non-CO poisoning burden from 1990 to 2021, showing significant reductions in prevalence, mortality, and DALYs, though regional disparities persist, especially in LMICs. Vulnerable populations, particularly children under five and the older adult, experienced a disproportionate burden, with males generally more affected than females. Future research should focus on agent-specific epidemiology, improving data collection in LMICs, and examining the impact of environmental and agricultural changes on poisoning risks. Interventions for vulnerable groups and integrating mental health factors into prevention strategies are crucial for further reducing the global burden.

## Data Availability

Publicly available datasets were analyzed in this study. This data can be found here: https://vizhub.healthdata.org/gbd-results/.

## Footnotes

^1^ https://vizhub.healthdata.org/gbd-results/

^2^ https://ghdx.healthdata.org/gbd-2021/sources

## Supplementary material

SUPPLEMENTARY Table S1 Prevalence of non-CO poisoning in 1990 and 2021 and the percentage change in the age-standardized prevalence rate (ASPR) per 100,000, by location.

SUPPLEMENTARY Table S2 Death due to non-CO poisoning in 1990 and 2021 and the percentage change in the age-standardized death rate (ASDR) per 100,000, by location.

SUPPLEMENTARY Table S3 DALY due to non-CO poisoning in 1990 and 2021 and the percentage change in the age-standardized DALY rate per 100,000, by location.

SUPPLEMENTARY Figure S1 Age-standardized disability-adjusted life year (DALY) rate of non-CO poisoning for 204 countries and territories, by Socio-Demographic Index (SDI), in 2021. Each point represents the observed DALY rate for a specific country or territory. The black line indicates the expected rate based on SDI and global patterns.

## References

[1] Tarhani F Nezami A Heidari G Hosseinizadeh-Salavati N. Epidemiological study of acute unintentional poisoning among children in Iran. Drug Res (Stuttg). (2022) 72:306–11. doi: 10.1055/a-1819-6453, PMID: 35605968

[2] Upchurch C Blumenberg A Brodie D MacLaren G Zakhary B Hendrickson RG. Extracorporeal membrane oxygenation use in poisoning: a narrative review with clinical recommendations. Clin Toxicol (Phila). (2021) 59:877–87. doi: 10.1080/15563650.2021.1945082, PMID: 34396873

[3] GBD 2021 Carbon Monoxide Poisoning Collaborators. Global, regional, and national mortality due to unintentional carbon monoxide poisoning, 2000-2021: results from the global burden of disease study 2021. Lancet Public Health. (2023) 8:e839–49. doi: 10.1016/S2468-2667(23)00185-8, PMID: 37813118 PMC10602911

[4] Cui P Jin Y Feng H Li Z Ding S Li Y. Burden of carbon monoxide poisoning in China, 1990-2019: a systematic analysis of data from the global burden of disease study 2019. Front Public Health. (2022) 10:930784. doi: 10.3389/fpubh.2022.930784, PMID: 35968482 PMC9371476

[5] Ross JA Woodfin MH Rege SV Holstege CP. Pediatric suicides reported to U.S. poison centers. Clin Toxicol (Phila). (2022) 60:869–71. doi: 10.1080/15563650.2022.2042013, PMID: 35240919

[6] Khan NU Khan U Khudadad U Ali A Raheem A Waheed S. Trends in mortality related to unintentional poisoning in the south Asian region from 1990 to 2019: analysis of data from the global burden of disease study. BMJ Open. (2023) 13:e062744. doi: 10.1136/bmjopen-2022-062744, PMID: 36754559 PMC9923325

[7] GBD 2019 Snakebite Envenomation Collaborators. Global mortality of snakebite envenoming between 1990 and 2019. Nat Commun. (2022) 13:6160. doi: 10.1038/s41467-022-33627-9, PMID: 36284094 PMC9596405

[8] Chelkeba L Mulatu A Feyissa D Bekele F Tesfaye BT. Patterns and epidemiology of acute poisoning in Ethiopia: systematic review of observational studies. Arch Public Health. (2018) 76:34. doi: 10.1186/s13690-018-0275-3, PMID: 29988616 PMC6027736

[9] Yenew C Shewaye M Yeshiwas AG Gebeyehu AA. Burden of chemical poisoning and contributing factors in the case of the Amhara region, Ethiopia. BMC Public Health. (2024) 24:2650. doi: 10.1186/s12889-024-20190-9, PMID: 39334010 PMC11437713

[10] Karunarathne A Bhalla A Sethi A Perera U Eddleston M. Importance of pesticides for lethal poisoning in India during 1999 to 2018: a systematic review. BMC Public Health. (2021) 21:1441. doi: 10.1186/s12889-021-11156-2, PMID: 34294076 PMC8296580

[11] Cowans C Love A Tangiisuran B Jacob SA. Uncovering the hidden burden of pharmaceutical poisoning in high-income and low-middle-income countries: a scoping review. Pharmacy (Basel). (2023) 11:184. doi: 10.3390/pharmacy11060184, PMID: 38133459 PMC10747954

[12] Lyden J Binswanger IA. The United States opioid epidemic. Semin Perinatol. (2019) 43:123–31. doi: 10.1053/j.semperi.2019.01.001, PMID: 30711195 PMC6578581

[13] Skolnick P. Treatment of overdose in the synthetic opioid era. Pharmacol Ther. (2022) 233:108019. doi: 10.1016/j.pharmthera.2021.108019, PMID: 34637841

[14] Plass D Beloconi A Bessems J Buekers J Kienzler S Martinez GS. Estimating the environmental burden of disease resulting from exposure to chemicals in European countries - potentials and challenges revealed in selected case studies. Environ Res. (2025) 269:120828. doi: 10.1016/j.envres.2025.120828, PMID: 39800302

[15] Roxburgh A Hall WD Dobbins T Gisev N Burns L Pearson S. Trends in heroin and pharmaceutical opioid overdose deaths in Australia. Drug Alcohol Depend. (2017) 179:291–8. doi: 10.1016/j.drugalcdep.2017.07.018, PMID: 28826104

[16] Organization WH. (2025). Prevention and management of cases of poisoning. Available online at: https://www.who.int/teams/environment-climate-change-and-health/chemical-safety-and-health/incidents-poisonings/prevention-and-management-of-cases-of-poisoning (Accessed July 19, 2025).

[17] Organization WH. (2025). Preventing and reducing poisoning. Available online at: https://www.who.int/europe/activities/preventing-and-reducing-poisoning (Accessed July 19, 2025).

[18] Dong J Li X. Lead pollution-related health of children in China: disparity, challenge, and policy. Sci Total Environ. (2023) 882:163383. doi: 10.1016/j.scitotenv.2023.163383, PMID: 37068684

[19] Institute for Health Metrics and Evaluation (IHME). (2025). Evaluation IfHMa. Available online at: https://www.healthdata.org/ (Accessed July 19, 2025).

[20] Global incidence, prevalence, years lived with disability (YLDs), disability-adjusted life-years (DALYs), and healthy life expectancy (HALE) for 371 diseases and injuries in 204 countries and territories and 811 subnational locations, 1990-2021: a systematic analysis for the global burden of disease study 2021. Lancet. (2024) 403:2133–61. doi: 10.1016/s0140-6736(24)00757-8, PMID: 38642570 PMC11122111

[21] Safiri S Carson-Chahhoud K Noori M Nejadghaderi SA Sullman MJM Ahmadian Heris J. Burden of chronic obstructive pulmonary disease and its attributable risk factors in 204 countries and territories, 1990-2019: results from the global burden of disease study 2019. BMJ. (2022) 378:e069679. doi: 10.1136/bmj-2021-069679, PMID: 35896191 PMC9326843

[22] Huang M Chen H Wang H Zhang Y Li L Lan Y. Global burden and risk factors of MASLD: trends from 1990 to 2021 and predictions to 2030. Intern Emerg Med. (2025) 20:1013–24. doi: 10.1007/s11739-025-03895-6, PMID: 40019669 PMC12130103

[23] Wei Y Lin Z Huang Q Wu H Wang R Wang J. Burden of female infertility in 204 countries and territories, 1990-2021: results from the global burden of disease study 2021. J Psychosom Obstet Gynaecol. (2025) 46:2459618. doi: 10.1080/0167482x.2025.2459618, PMID: 39936646

[24] Kyei EF Zhang L Ansong R Kyei GK. Empowering communities and enhancing public safety: stakeholders' perspectives on opioid overdose prevention strategies in Boston. Community Ment Health J. (2024) 61:834–46. doi: 10.1007/s10597-024-01420-1, PMID: 39648182

[25] Kouli M Al Houri HN Jomaa S Issa A Arrouk DMN Alhouri A. Epidemiology of poisoning in Syria (1999 through 2020). Clin Toxicol (Phila). (2023) 61:116–22. doi: 10.1080/15563650.2022.2156882, PMID: 36524826

[26] Gummin DD Mowry JB Beuhler MC Spyker DA Rivers LJ Feldman R. 2022 annual report of the National Poison Data System(®) (NPDS) from America's poison centers(®): 40th annual report. Clin Toxicol (Phila). (2023) 61:717–939. doi: 10.1080/15563650.2023.2268981, PMID: 38084513

[27] Mottla ME Bowler ME Asgary R. Epidemiology, risk factors, and strategies to prevent and manage poisonings due to pharmaceuticals in children in low income and low-middle income countries: a systematic review. J Glob Health. (2023) 13:04173. doi: 10.7189/jogh.13.04173, PMID: 38154015 PMC10754493

[28] Zhou Z Mohamed F. Pharmacist gatekeeper interventions for suicide prevention: how evidence from developed countries support their role in low- and middle-income countries. Front Psychol. (2024) 15:1508621. doi: 10.3389/fpsyt.2024.1508621, PMID: 39935627 PMC11810978

[29] Asrie AB Atnafie SA Getahun KA Birru EM Mekonnen GB Alemayehu GA. Poisoning cases and their management in Amhara National Regional State, Ethiopia: hospital-based prospective study. PLoS One. (2024) 19:e0303438. doi: 10.1371/journal.pone.0303438, PMID: 38820326 PMC11142576

[30] Telayneh AT Habtegiorgis SD Birhanu MY Sume BW Ayenew T Gedif G. Mortality of acute poisoning and its predictors in Ethiopia: a systematic review and meta-analysis. Heliyon. (2024) 10:e29741. doi: 10.1016/j.heliyon.2024.e29741, PMID: 38681614 PMC11046229

[31] Brassell M Karunarathne A Utyasheva L Eddleston M Konradsen F Rother HA. Current pesticide suicide surveillance methods used across the African continent: a scoping review protocol. BMJ Open. (2022) 12:e055923. doi: 10.1136/bmjopen-2021-055923, PMID: 35981770 PMC9394204

[32] Thal F Reinhold T. Advice for lay callers with low-risk poison exposures by a regional poison control center: the impact on health care expenditures. Arch Public Health. (2022) 80:243. doi: 10.1186/s13690-022-00994-0, PMID: 36451203 PMC9713099

[33] Diendéré E Turgeon AF Gagnon-Labelle K Couture A Neveu X St-Onge M. Clinical outcomes of indigenous versus non-indigenous patients: a multicenter retrospective cohort study in the province of Quebec. J Prim Care Community Health. (2023) 14:21501319231178654. doi: 10.1177/21501319231178654, PMID: 37283306 PMC10262648

[34] Rivera BD Friedman SR. What would it really take to solve the overdose epidemic in the United States? Int J Drug Policy. (2024) 128:104435. doi: 10.1016/j.drugpo.2024.104435, PMID: 38729061 PMC11220856

[35] Rezaee M Esfahani Z Nejadghaderi SA Abbasi-Kangevari M Saeedi Moghaddam S Ghanbari A. Estimating the burden of diseases attributable to lead exposure in the North Africa and Middle East region, 1990-2019: a systematic analysis for the global burden of disease study 2019. Environ Health. (2022) 21:105. doi: 10.1186/s12940-022-00914-3, PMID: 36309664 PMC9617306

[36] Kou J Li X Zhang M Wang L Hu L Liu X. Accumulative levels, temporal and spatial distribution of common chemical pollutants in the blood of Chinese adults. Environ Pollut. (2022) 311:119980. doi: 10.1016/j.envpol.2022.119980, PMID: 35985432

[37] Batyrova G Tlegenova Z Kononets V Umarova G Kudabayeva K Bazargaliyev Y. Hair toxic trace elements of residents across the Caspian oil and gas region of Kazakhstan: cross-sectional study. Int J Environ Res Public Health. (2022) 19:1158. doi: 10.3390/ijerph191811158, PMID: 36141431 PMC9517423

[38] Klafke F Barros VG Henning E. Solid waste management and *Aedes aegypti* infestation interconnections: a regression tree application. Waste Manag Res. (2023) 41:1684–96. doi: 10.1177/0734242x231164318, PMID: 37013436

[39] Li H Li Y. The impact of digital economy development on public health: evidence from Chinese cities. Front Public Health. (2024) 12:1347572. doi: 10.3389/fpubh.2024.1347572, PMID: 39071150 PMC11272619

[40] Torabi Z Shakibazadeh E Tajvar M Rezaei N. Non-communicable diseases challenges and opportunities in Iran: a qualitative study. Sci Rep. (2025) 15:8975. doi: 10.1038/s41598-025-92897-7, PMID: 40089611 PMC11910664

[41] Salem W Abdulrouf P Thomas B Elkassem W Abushanab D Rahman Khan H. Epidemiology, clinical characteristics, and associated cost of acute poisoning: a retrospective study. J Pharm Policy Pract. (2024) 17:2325513. doi: 10.1080/20523211.2024.2325513, PMID: 38741897 PMC11089918

[42] Ali FMH Nikoloski Z Gjebrea O Mossialos E. Trends and inequalities in health system satisfaction: results from the latest nationally representative surveys in Qatar. Int J Equity Health. (2024) 23:249. doi: 10.1186/s12939-024-02317-x, PMID: 39587627 PMC11590323

[43] Gasciauskaite G Lunkiewicz J Tucci M Von Deschwanden C Nöthiger CB Spahn DR. Environmental and economic impact of sustainable anaesthesia interventions: a single-Centre retrospective observational study. Br J Anaesth. (2024) 133:1449–58. doi: 10.1016/j.bja.2023.11.049, PMID: 38177005

[44] Graabak G Næss LE Laugsand LE Bjørnsen LP. Intoxication cases in the emergency department at a Norwegian university hospital 2019-20. Tidsskr Nor Laegeforen. (2024) 144:417. doi: 10.4045/tidsskr.23.0417 38934322

[45] Nyman AAT Bogstrand ST Clausen T Edvardsen HME. Toxicological findings in overdose suicides 2016-21. Tidsskr Nor Laegeforen. (2025) 145:836. doi: 10.4045/tidsskr.23.0836, PMID: 39932090

[46] Amundsen EJ Melsom AM Eriksen BO Løchen ML. No decline in drug overdose deaths in Norway: an ecological approach to understanding at-risk groups and the impact of interventions. Nordisk Alkohol Nark. (2024) 41:111–30. doi: 10.1177/14550725231195413, PMID: 38356787 PMC10863554

[47] Myhre M Kildahl AT Astrup H Bergsager D Walby FA. Suicide in services for mental health and substance use: a national hybrid registry surveillance system. Health Inform J. (2023) 29:14604582231167439. doi: 10.1177/14604582231167439, PMID: 36989536

[48] Dabholkar S Pirani S Davis M Khan M Eddleston M. Suicides by pesticide ingestion in Pakistan and the impact of pesticide regulation. BMC Public Health. (2023) 23:676. doi: 10.1186/s12889-023-15505-1, PMID: 37041526 PMC10088141

[49] Eldhose R Viggeswarpu S Jambugulam M. Unmasking herbal medication-induced lead poisoning in a geriatric patient with gastrointestinal symptoms. BMJ Case Rep. (2023) 16:8065. doi: 10.1136/bcr-2023-258065, PMID: 38086570 PMC10728943

[50] Ahmed GEM Ahmed EYM Ahmed AE Hemmeda L Birier AB Abdelgadir T. Prevalence and reasons to seek traditional healing methods among residents of two localities in North Kordofan state, Sudan 2022: a cross-sectional study. Health Sci Rep. (2023) 6:e1487. doi: 10.1002/hsr2.1487, PMID: 37621385 PMC10444970

[51] Zúñiga-Venegas LA Hyland C Muñoz-Quezada MT Quirós-Alcalá L Butinof M Buralli R. Mora AM. Health effects of pesticide exposure in Latin American and the Caribbean populations: a scoping review. Environ Health Perspect. (2022) 130:9934. doi: 10.1289/ehp9934, PMID: 36173136 PMC9521041

[52] Landeros N Duk S Márquez C Inzunza B Acuña-Rodríguez IS Zúñiga-Venegas LA. Genotoxicity and reproductive risk in workers exposed to pesticides in rural areas of Curicó, Chile: a pilot study. Int J Environ Res Public Health. (2022) 19:6608. doi: 10.3390/ijerph192416608, PMID: 36554491 PMC9779056

[53] Nguyen SN Vu LT Nguyen HT Nguyen LMT. Childhood acute poisoning at Haiphong children's hospital: a 10-year retrospective study. Int J Pediatr. (2023) 2023:2130755. doi: 10.1155/2023/2130755, PMID: 37700774 PMC10495236

[54] Robledo-Aceves M Corona-Gutiérrez AA Camarena-Pulido EE Barrón-Balderas A Meza-López C Ramos-Gutiérrez RY. Epidemiology of acute childhood poisoning in pediatric emergencies in Western Mexico. Bol Med Hosp Infant Mex. (2024) 81:287–93. doi: 10.24875/bmhim.23000183, PMID: 39378401

[55] Bosshart N Bearth A Wermelinger S Daum M Siegrist M. Seeing household chemicals through the eyes of children-investigating influential factors of preschoolers' perception and behavior. J Saf Res. (2022) 83:400–9. doi: 10.1016/j.jsr.2022.09.015, PMID: 36481033

[56] Bosshart N Bearth A Stutz SE Wermelinger S Daum MM Siegrist M. Avoiding unintentional injuries from household chemicals: comparing the appeal to children from the perspectives of children, caregivers, and experts. Appl Ergon. (2025) 122:104401. doi: 10.1016/j.apergo.2024.104401, PMID: 39396407

[57] Perveen F Ahmed N Masud S Ihsan MU Khan UR Khan NU. Parental knowledge attitude and practices about chemical and medicinal poisons: a hospital based study from Karachi, Pakistan. Injury. (2023) 54:110481. doi: 10.1016/j.injury.2022.11.024, PMID: 37573064

[58] Shimony-Kanat S Orr D Falk A. Social and economic factors associated with child unintentional injury mortality in high-income countries. Inj Prev. (2024) 30:194–9. doi: 10.1136/ip-2023-045016, PMID: 38050075

[59] Yu X Qian Y Zhang Y Chen Y Wang M. Association between polypharmacy and cognitive impairment in older adults: a systematic review and meta-analysis. Geriatr Nurs. (2024) 59:330–7. doi: 10.1016/j.gerinurse.2024.07.005, PMID: 39111065

[60] GBD 2016 Alcohol and Drug Use Collaborators. The global burden of disease attributable to alcohol and drug use in 195 countries and territories, 1990-2016: a systematic analysis for the global burden of disease study 2016. Lancet Psychiatry. (2018) 5:987–1012. doi: 10.1016/s2215-0366(18)30337-7, PMID: 30392731 PMC6251968

[61] Mason M Pandya K Lundberg A. Older adult drug overdose: an application of latent class analysis to identify prevention opportunities. Harm Reduct J. (2024) 21:61. doi: 10.1186/s12954-024-00973-4, PMID: 38481307 PMC10936079

[62] El Zahran T Hammoud L Salam R Salam Y Kazzi Z Tamim H. Sex differences among patients with intentional poisoning presenting to the emergency department at a tertiary care Centre in Beirut, Lebanon. Basic Clin Pharmacol Toxicol. (2024) 135:353–63. doi: 10.1111/bcpt.14039, PMID: 38965652

[63] Abebe MT Kumie A Ayana SW Assefa T Ambaw W. Assessment of occupational exposure to lead among workers engaged in a city bus garage in Addis Ababa, Ethiopia: a comparative cross-sectional study. J Occup Med Toxicol. (2024) 19:26. doi: 10.1186/s12995-024-00422-9, PMID: 38902821 PMC11188258

[64] Llorens P Lirón-García Á Santos-Redondo M Marín-Aparicio J Espinosa B Martínez E. Adherence to quality indicators for emergency department treatment of acute poisoning according to patient sex. Emergencias. (2024) 36:97–103. doi: 10.55633/s3me/028.2024, PMID: 38597616

[65] Lirón-García Á Ramos-Rincón JM Valero-Novella B Marín-Aparicio J Sánchez-Martínez R Llorens P. Epidemiology and quality of care of acute intoxication in people over 65 years of age in Alicante, Spain. Rev Clin Esp (Barc). (2023) 223:610–8. doi: 10.1016/j.rceng.2023.10.006, PMID: 39491180

[66] Fan L Wang X Lv T Xue F Wu B Ma A. Follow-up of patients with a 5-year survival after paraquat poisoning using computed tomography images and spirometry. Hum Exp Toxicol. (2023) 42:9603271221150243. doi: 10.1177/09603271221150243, PMID: 36622665

[67] Merrill RM Ashton MK. How do mental disorders and combinations of disorders affect the odds of injuries and poisoning? J Nerv Ment Dis. (2024) 212:303–11. doi: 10.1097/nmd.0000000000001771, PMID: 38704650

[68] Eddleston M Nagami H Lin CY Davis ML Chang SS. Pesticide use, agricultural outputs, and pesticide poisoning deaths in Japan. Clin Toxicol (Phila). (2022) 60:933–41. doi: 10.1080/15563650.2022.2064868, PMID: 35475715

[69] Choi S Kim GW Lim H. A narrative review of contemporary lethal pesticides: unveiling the ongoing threat of pesticide poisoning. Clin Exp Emerg Med. (2024) 11:335–48. doi: 10.15441/ceem.23.167, PMID: 38286498 PMC11700689

[70] Noghrehchi F Dawson AH Raubenheimer J Mohamed F Gawarammana IB Eddleston M. Restrictions on pesticides and deliberate self-poisoning in Sri Lanka. JAMA Netw Open. (2024) 7:e2426209. doi: 10.1001/jamanetworkopen.2024.26209, PMID: 39106063 PMC11304112

[71] Suphim B Songthap A. Factors affecting safe pesticide-use behaviors among farm plant agriculturists in northeastern Thailand. BMC Public Health. (2024) 24:1096. doi: 10.1186/s12889-024-18662-z, PMID: 38643084 PMC11032588

[72] Bajaj A Han D Elman I Thanos PK Dennen CA Badgaiyan RD. Positive clinical outcomes for severe reported pain using robust non-addictive home electrotherapy-a case-series. J Pers Med. (2023) 13:336. doi: 10.3390/jpm13020336, PMID: 36836570 PMC9965228

[73] Biancuzzi H Dal Mas F Brescia V Campostrini S Cascella M Cuomo A. Opioid misuse: a review of the Main issues, challenges, and strategies. Int J Environ Res Public Health. (2022) 19:1754. doi: 10.3390/ijerph191811754, PMID: 36142028 PMC9517221

[74] Ledlie S Juurlink DN Tadrous M Mamdani M Paterson JM Gomes T. Opioid-related deaths between 2019 and 2021 across 9 Canadian provinces and territories. CMAJ. (2024) 196:E469–e476. doi: 10.1503/cmaj.231339, PMID: 38621782 PMC11019600

[75] Brett J Gillies MB Buckley NA Pearson SA Zoega H. Patterns of suboptimal antipsychotic use and misuse in Australia: what can routinely collected data tell us? Br J Clin Pharmacol. (2023) 89:3411–20. doi: 10.1111/bcp.15821, PMID: 37309058 PMC10953398

[76] Leung KHB Grunau BE Lee MK Buxton JA Helmer J van Diepen S. Optimizing placement of public-access naloxone kits using geospatial analytics: a modelling study. CMAJ. (2025) 197:E258–e265. doi: 10.1503/cmaj.241228, PMID: 40097007 PMC11913478

[77] Xiong S Wu A Weng L Zhang L Wu M Li H. Study on the correlation between the number of mushroom poisoning cases and meteorological factors based on the generalized additive model in Guizhou Province, 2023. BMC Public Health. (2024) 24:2628. doi: 10.1186/s12889-024-20050-6, PMID: 39333979 PMC11437644

[78] Miao T Li X Hu B Zhang H Xu L Zhang D. An outbreak of foodborne botulism caused by *Clostridium botulinum* BoNT/A3 in pickled eggs - Weihai City, Shandong Province, China, 2024. China CDC Wkly. (2024) 6:1375–80. doi: 10.46234/ccdcw2024.273, PMID: 39802083 PMC11724129

[79] Zhang H Guo Y Chen L Liu Z Liang J Shi M. Epidemiology of foodborne bongkrekic acid poisoning outbreaks in China, 2010 to 2020. PLoS One. (2023) 18:e0279957. doi: 10.1371/journal.pone.0279957, PMID: 36630445 PMC9833544

[80] Pacini A Tsutaoka B Lai L Durrani TS. Unintentional pediatric exposures to household cleaning products: a cross-sectional analysis of the National Poison Data System (2000-2015). J Occup Med Toxicol. (2023) 18:16. doi: 10.1186/s12995-023-00384-4, PMID: 37568177 PMC10422824

[81] Bosshart N Bearth A Wermelinger S Daum MM Siegrist M. Childhood poisonings: effects of ambiguous product characteristics on preschool children's categorization of household chemicals. Risk Anal. (2024) 44:1193–203. doi: 10.1111/risa.14217, PMID: 37698161

[82] Zheng K Zeng Z Tian Q Huang J Zhong Q Huo X. Epidemiological evidence for the effect of environmental heavy metal exposure on the immune system in children. Sci Total Environ. (2023) 868:161691. doi: 10.1016/j.scitotenv.2023.161691, PMID: 36669659

[83] Yawei S Jianhai L Junxiu Z Xiaobo P Zewu Q. Epidemiology, clinical presentation, treatment, and follow-up of chronic mercury poisoning in China: a retrospective analysis. BMC Pharmacol Toxicol. (2021) 22:25. doi: 10.1186/s40360-021-00493-y, PMID: 33941274 PMC8091676

[84] An Q Wang Q Liu R Zhang J Li S Shen W. Analysis of relationship between mixed heavy metal exposure and early renal damage based on a weighted quantile sum regression and Bayesian kernel machine regression model. J Trace Elem Med Biol. (2024) 84:127438. doi: 10.1016/j.jtemb.2024.127438, PMID: 38520795

[85] Dani A Balachandran S McGill K Whyte I Carter G. Prevalence of depression and predictors of discharge to a psychiatric Hospital in Young People with hospital-treated deliberate self-poisoning at an Australian sentinel unit. Int J Environ Res Public Health. (2022) 19:5733. doi: 10.3390/ijerph192315753, PMID: 36497828 PMC9737120

[86] Lim JS Buckley NA Cairns R Schumann J Schaffer AL Chitty KM. Substances detected during coroner postmortem toxicology analyses in poisoning- and nonpoisoning-related suicides. JAMA Psychiatry. (2023) 80:1121–30. doi: 10.1001/jamapsychiatry.2023.2289, PMID: 37494023 PMC10372754

[87] Oladunjoye AF Li E Aneni K Onigu-Otite E. Cannabis use disorder, suicide attempts, and self-harm among adolescents: a national inpatient study across the United States. PLoS One. (2023) 18:e0292922. doi: 10.1371/journal.pone.0292922, PMID: 37847698 PMC10581466

[88] Zheng W Han L Fan Y Yi M Lu X Yang J. Association of mental health status between self-poisoning suicide patients and their family members: a matched-pair analysis. BMC Psychiatry. (2023) 23:294. doi: 10.1186/s12888-023-04779-9, PMID: 37118663 PMC10144897

