# Supplementary material for: Burden of non-CO poisoning in 204 countries and territories, 1990–2021: results from the global burden of disease study 2021
Source: Front Public Health. 2025 Jul 25;13:1620523. doi: 10.3389/fpubh.2025.1620523 (PMC12331598; doi:10.3389/fpubh.2025.1620523)

**Supplementary table and figure legends**

**Table S1:** Prevalence of non-CO poisoning in 1990 and 2021 and the percentage change in the age-standardized prevalence rate (ASPR) per 100,000, by location

**Table S2:** Death due to non-CO poisoning in 1990 and 2021 and the percentage change in the age-standardized death rate (ASDR) per 100,000, by location

**Table S3:** DALY due to non-CO poisoning in 1990 and 2021 and the percentage change in the age-standardized DALY rate per 100,000, by location

**Figure S1:** Age-standardized disability-adjusted life year (DALY) rate of non-CO poisoning for 204 countries and territories, by Socio-Demographic Index (SDI), in 2021.Each point represents the observed age-standardized DALY rate for a specific country or territory in 2021. The black line indicates the expected DALY rate based on the SDI and global disease patterns.

| **Table S1:Prevalence of non-CO poisoning in 1990 and 2021 and the percentage change in the age-standardized prevalence rate (ASPR) per 100,000, by location** | | | | | |
| --- | --- | --- | --- | --- | --- |
|  | Prevalence_Number_1990.95._UI. | ASPRs_1990_per_100000.95._UI. | Prevalence_Number_2021.95._UI. | ASPRs_2021_per_100000.95._UI. | Percentage_change_in_the_ASPRs_from_1990_to_2021_per_100000 |
| Global | 3752769 (3184652,4679041) | 77.3 (65.5,95.7) | 3582786 (3045510,4448134) | 43.3 (36.8,53.7) | -43.9 (-44.6,-43.1) |
| Central Asia | 71523 (57779,91540) | 113.8 (92.3,146.8) | 71835 (56468,93477) | 75.3 (59.1,98) | -33.8 (-36,-31.5) |
| Armenia | 2608 (2063,3426) | 78.8 (62.6,103.5) | 2087 (1640,2701) | 62.9 (49.2,81.3) | -20.2 (-23.7,-16.6) |
| Azerbaijan | 6395 (5148,8397) | 93.7 (75.6,123.1) | 7875 (6246,10398) | 70 (55.5,92.2) | -25.3 (-27.6,-22.7) |
| Georgia | 6859 (5584,9070) | 118.9 (96.6,156.9) | 4588 (3680,5864) | 115.5 (92.1,146.4) | -2.8 (-7.2,2.2) |
| Kazakhstan | 23359 (19102,29402) | 148.3 (121.5,187.1) | 15930 (12463,20433) | 83.6 (65.2,107.2) | -43.6 (-46.9,-40.1) |
| Kyrgyzstan | 4355 (3533,5470) | 109 (88.8,138) | 4402 (3427,5804) | 67.5 (52.7,89.5) | -38.1 (-41.5,-34.8) |
| Mongolia | 4359 (3550,5580) | 240.9 (197.4,310.5) | 2677 (2144,3510) | 83.6 (66.9,110) | -65.3 (-67.5,-63.2) |
| Tajikistan | 4369 (3484,5688) | 99.4 (80.1,131) | 6850 (5353,8919) | 72.8 (56.9,95.4) | -26.8 (-30.4,-23.4) |
| Turkmenistan | 3060 (2424,3907) | 95.7 (76.6,123.9) | 3605 (2860,4712) | 71 (56.4,92.8) | -25.8 (-28.9,-23) |
| Uzbekistan | 16159 (12739,20705) | 89.7 (71.2,116.4) | 23821 (18875,31327) | 70.6 (55.9,92.7) | -21.3 (-23.8,-18.2) |
| Central Europe | 322264 (259745,405321) | 245.9 (198.1,309.9) | 177517 (140720,226732) | 138 (108.8,178.4) | -43.9 (-46,-41.9) |
| Albania | 5527 (4400,6996) | 175.4 (139.8,220.6) | 4225 (3338,5373) | 144.3 (114.2,185.8) | -17.7 (-20.7,-15.5) |
| Bosnia and Herzegovina | 15828 (12752,19860) | 339.6 (273.8,424.3) | 6119 (4768,8004) | 167 (129.5,218.3) | -50.8 (-54.4,-46.3) |
| Bulgaria | 19815 (15889,24787) | 213.6 (171.1,269.1) | 10665 (8410,13554) | 140.6 (110.4,180.1) | -34.2 (-37.7,-30.6) |
| Croatia | 8341 (6546,10649) | 160.7 (126,206.1) | 6305 (4851,8253) | 135.4 (103.5,179.7) | -15.8 (-19.5,-11.7) |
| Czechia | 28734 (22861,36513) | 261.2 (208.7,333.5) | 21568 (16629,28077) | 183.9 (140.8,240) | -29.6 (-33.1,-24.8) |
| Hungary | 21906 (17257,27716) | 196.3 (155,250.4) | 16370 (12781,21155) | 153.5 (118.8,200.3) | -21.8 (-25.1,-18.5) |
| Montenegro | 1133 (887,1452) | 176.8 (138.5,227.1) | 990 (768,1275) | 147.8 (114.2,190.4) | -16.4 (-20.9,-12.7) |
| North Macedonia | 3561 (2821,4526) | 175.2 (138.9,223) | 3495 (2760,4479) | 144.9 (114.2,187.5) | -17.3 (-20.7,-13.6) |
| Poland | 84554 (68175,108838) | 212.4 (171.3,273.7) | 45324 (36132,60084) | 104.4 (83,137.9) | -50.8 (-52.8,-48.6) |
| Romania | 94181 (76006,117715) | 387.2 (312.5,485.9) | 31513 (24942,39713) | 149.8 (119.5,188.6) | -61.3 (-63.5,-58.9) |
| Serbia | 19474 (15440,24797) | 192.7 (152.7,246.3) | 15476 (11957,19896) | 156.1 (120.3,203.7) | -19 (-22.8,-12.8) |
| Slovakia | 10127 (8075,12858) | 185.3 (147.7,235.6) | 8891 (6872,11357) | 148.2 (114.1,190) | -20 (-23.7,-16.1) |
| Slovenia | 3930 (3059,5165) | 187.1 (145.8,247) | 3993 (3100,5115) | 173.7 (133.4,225.2) | -7.2 (-10.5,-1.8) |
| Eastern Europe | 281746 (222879,381410) | 117 (92.4,157.4) | 158892 (127908,220254) | 66.5 (52.9,91.7) | -43.2 (-46.8,-40) |
| Belarus | 12107 (9545,15770) | 109.4 (86.3,141.6) | 7735 (6014,10426) | 72.7 (56.4,97.1) | -33.5 (-36.7,-30.5) |
| Estonia | 1724 (1359,2310) | 102.5 (80.3,136.6) | 1319 (1017,1749) | 88.8 (68.1,118.3) | -13.4 (-17.8,-4.6) |
| Latvia | 2769 (2163,3662) | 96.6 (75.2,126.8) | 1798 (1396,2375) | 84.2 (64.9,112.4) | -12.9 (-17.1,-5.5) |
| Lithuania | 4394 (3454,5700) | 113 (88.8,145.5) | 2411 (1904,3218) | 76.1 (59.9,100.5) | -32.7 (-36.7,-28.9) |
| Republic of Moldova | 7056 (5696,8731) | 157.3 (126.9,194.8) | 3670 (2896,4749) | 89.2 (70.3,113.4) | -43.3 (-47.2,-39.6) |
| Russian Federation | 205912 (162253,278359) | 128.8 (101.4,173) | 110743 (88835,154639) | 66.8 (53.1,92.6) | -48.2 (-52.1,-44.7) |
| Ukraine | 47784 (38543,65709) | 83.3 (67,114.6) | 31215 (25312,43121) | 60.5 (48.8,83.5) | -27.4 (-31,-24.6) |
| Australasia | 27920 (22935,36164) | 132.4 (108.7,172.2) | 38334 (31399,49280) | 113.9 (92.7,146) | -14 (-16.4,-10.5) |
| Australia | 21774 (17800,28415) | 123.9 (101.3,161.7) | 30789 (25141,39858) | 109.2 (88.6,141.4) | -11.9 (-14.9,-8.3) |
| New Zealand | 6146 (5096,7796) | 174.5 (144.6,221.9) | 7545 (6272,9438) | 136.9 (113.8,171.4) | -21.5 (-25,-17.7) |
| High-income Asia Pacific | 365699 (306185,462920) | 194.2 (163.1,245.6) | 296715 (248882,373303) | 127 (106,160.8) | -34.6 (-36.1,-32.9) |
| Brunei Darussalam | 413 (345,518) | 181.6 (152.6,224.9) | 602 (501,751) | 127.4 (106.5,158) | -29.9 (-32.5,-27.5) |
| Japan | 256177 (213257,324922) | 180.6 (151,229) | 213568 (179217,269091) | 131.3 (109.3,167.3) | -27.3 (-28.7,-25.9) |
| Republic of Korea | 103959 (87685,131032) | 240.5 (203.7,299.8) | 72838 (60831,91578) | 115.6 (96.2,145.4) | -51.9 (-54.2,-49.6) |
| Singapore | 5151 (4290,6436) | 165.1 (138.1,204.9) | 9706 (8085,12188) | 142.9 (119.9,179.7) | -13.4 (-15.7,-10.3) |
| High-income North America | 652184 (544451,809942) | 214.7 (179.5,265.7) | 655449 (549229,796045) | 147.2 (123,181.1) | -31.5 (-33.8,-29.4) |
| Canada | 59156 (48733,72516) | 197.2 (163.1,240.9) | 71067 (59399,85838) | 150.9 (125.6,182.6) | -23.5 (-26,-21) |
| Greenland | 112 (94,135) | 213.4 (180,254.7) | 79 (66,95) | 124.7 (104.3,150.7) | -41.6 (-43.1,-40.1) |
| United States of America | 592900 (495220,737893) | 216.6 (180.9,268.8) | 584293 (489623,709620) | 146.7 (122.6,180.7) | -32.3 (-34.8,-30) |
| Southern Latin America | 165083 (134159,210906) | 337.5 (274.8,431.4) | 193606 (160252,249284) | 269.7 (222.7,349.4) | -20.1 (-25.4,-17.6) |
| Argentina | 126754 (103023,161353) | 386.9 (314.8,491.9) | 139033 (115002,178785) | 289.8 (239.6,374.6) | -25.1 (-31.6,-22.2) |
| Chile | 29832 (24401,38562) | 230.8 (189.6,296.9) | 46373 (37406,59723) | 231.2 (185.7,297.7) | 0.2 (-5.9,5.8) |
| Uruguay | 8490 (6926,10886) | 263.1 (214.5,338.3) | 8189 (6651,10642) | 220.8 (178.8,288) | -16.1 (-18.9,-12.6) |
| Western Europe | 699756 (586805,853183) | 166.1 (138.6,204) | 641146 (537572,787399) | 126.9 (105.5,156.7) | -23.6 (-24.9,-22.4) |
| Andorra | 78 (65,95) | 132.9 (110.4,163.6) | 116 (97,143) | 113.8 (94.1,141.7) | -14.4 (-17,-11.5) |
| Austria | 13641 (11438,16436) | 161.4 (135.2,194.1) | 14656 (12336,17661) | 141.5 (118.3,172) | -12.4 (-15.1,-8.9) |
| Belgium | 25996 (21841,31731) | 237.1 (198.2,289.4) | 18361 (15492,22285) | 139.4 (116.6,169.1) | -41.2 (-43.5,-39) |
| Cyprus | 1140 (961,1390) | 143.1 (120.7,174.4) | 1738 (1443,2123) | 112.8 (93,139.9) | -21.2 (-24.3,-17.5) |
| Denmark | 7293 (6110,8880) | 128.2 (107.2,156.7) | 7984 (6655,9879) | 119.2 (98.4,148.2) | -7.1 (-10.3,-3.8) |
| Finland | 6069 (5033,7520) | 112 (93.1,139.4) | 6607 (5544,8022) | 102.7 (85.9,125.7) | -8.3 (-11.5,-4.6) |
| France | 86024 (72119,106197) | 138.2 (115.8,172.5) | 90901 (76316,110400) | 119.6 (99.7,148.1) | -13.4 (-16.7,-10.6) |
| Germany | 110595 (92086,135490) | 123.9 (102.9,153.2) | 113270 (94371,139460) | 113.2 (93.7,141.8) | -8.6 (-11.3,-5.4) |
| Greece | 14252 (11914,17175) | 125.3 (104.6,152.6) | 12872 (10786,16005) | 106.7 (89,134.7) | -14.8 (-17.8,-10.4) |
| Iceland | 396 (333,485) | 151.9 (127.9,185.8) | 486 (404,597) | 124.9 (103.1,154.1) | -17.7 (-20,-14.6) |
| Ireland | 5216 (4369,6381) | 142.3 (119.1,174) | 6804 (5654,8442) | 123.9 (102.5,155.1) | -13 (-15.6,-8.5) |
| Israel | 6772 (5660,8309) | 139.1 (116.3,170.9) | 10778 (8929,13262) | 109.6 (90.5,135.3) | -21.2 (-24.5,-17.6) |
| Italy | 141996 (118805,175859) | 223.5 (186.9,277.5) | 92846 (78867,112654) | 128.2 (107.8,156) | -42.6 (-44.5,-40.9) |
| Luxembourg | 626 (525,761) | 148.5 (124.6,180.4) | 828 (684,1021) | 112.9 (93.1,139.4) | -24 (-26.9,-19.9) |
| Malta | 547 (456,666) | 140.2 (116.7,172) | 619 (516,752) | 119.6 (99.2,147.9) | -14.7 (-17.5,-11.6) |
| Monaco | 105 (90,126) | 282.7 (239.2,342.2) | 84 (70,99) | 172.7 (144,207.3) | -38.9 (-41.4,-36) |
| Netherlands | 20129 (16929,24562) | 125.5 (105.5,153.3) | 21391 (17777,26358) | 108.5 (89,134.9) | -13.5 (-16.5,-10.1) |
| Norway | 14445 (12144,17725) | 323.3 (271.9,396.1) | 25718 (21507,31877) | 443.6 (369.6,551.9) | 37.2 (32.6,41.3) |
| Portugal | 20266 (16924,24675) | 185.9 (154.6,227.9) | 15484 (12995,19049) | 123.4 (102.7,152.4) | -33.6 (-36.1,-31.6) |
| San Marino | 52 (44,63) | 199.2 (166.9,239.6) | 66 (56,80) | 166 (139.2,206.2) | -16.7 (-19.5,-12.8) |
| Spain | 63574 (53406,76941) | 151.5 (127.4,183.9) | 62159 (51506,77661) | 116 (95.9,146.5) | -23.5 (-26.8,-19.3) |
| Sweden | 23704 (19985,28476) | 246.8 (207.9,297.2) | 20296 (17122,24784) | 170.2 (143.4,208.4) | -31 (-33,-28.9) |
| Switzerland | 8329 (6976,10197) | 110.2 (92.1,136.2) | 11281 (9414,13785) | 109.5 (91.2,137.2) | -0.7 (-4.5,3) |
| United Kingdom | 127936 (107494,157245) | 204.2 (171.4,251.1) | 105236 (88398,129249) | 136.5 (113.8,169.6) | -33.2 (-34.8,-31.9) |
| Andean Latin America | 32799 (27537,40776) | 98.8 (82.8,122.7) | 39999 (33441,50635) | 60.3 (50.5,76.2) | -38.9 (-41,-36.3) |
| Bolivia (Plurinational State of) | 5517 (4589,6819) | 101.4 (84.3,125.6) | 6747 (5626,8491) | 58.8 (49.2,73.9) | -42 (-44.8,-39.3) |
| Ecuador | 7221 (6033,9023) | 83.3 (70.3,104) | 11144 (9266,14036) | 61.8 (51.5,77.7) | -25.8 (-27.7,-23.6) |
| Peru | 20061 (16782,25025) | 105.1 (88,130.2) | 22108 (18613,28264) | 60.1 (50.5,76.7) | -42.9 (-45.3,-39.6) |
| Caribbean | 31270 (26289,38784) | 94.1 (78.9,116) | 38036 (31700,48140) | 76.6 (63.7,96.5) | -18.7 (-21,-16.5) |
| Antigua and Barbuda | 60 (50,74) | 103 (86,126.4) | 64 (53,79) | 65 (54,80.9) | -36.9 (-39.9,-33.9) |
| Bahamas | 196 (164,248) | 80.2 (67.1,100.7) | 250 (205,325) | 59.8 (49,77.8) | -25.4 (-29,-21.8) |
| Barbados | 223 (186,277) | 85.4 (71.2,106.2) | 225 (185,292) | 66.2 (54.2,86.7) | -22.5 (-25.9,-18.3) |
| Belize | 213 (179,261) | 135.4 (113.1,163.6) | 244 (202,304) | 58.2 (48.3,72.2) | -57 (-60.1,-53.9) |
| Bermuda | 83 (70,101) | 129.4 (110.1,159.6) | 52 (43,66) | 68.3 (56.4,88.5) | -47.2 (-50.4,-44.1) |
| Cuba | 8697 (7200,10961) | 78.3 (65.1,98) | 12793 (10470,16584) | 98.4 (80.7,127.6) | 25.7 (21,30.7) |
| Dominica | 136 (115,168) | 206.1 (174,253.9) | 77 (64,93) | 104.1 (87.8,125.9) | -49.5 (-51.8,-47.2) |
| Dominican Republic | 5281 (4369,6638) | 82.1 (68.7,102) | 6990 (5850,8730) | 63 (52.8,78.6) | -23.4 (-26.8,-19.2) |
| Grenada | 109 (92,131) | 141.8 (119.8,169.7) | 71 (59,90) | 64.4 (53.5,82.3) | -54.6 (-57.9,-51.1) |
| Guyana | 542 (453,667) | 78.7 (66.1,96.3) | 444 (365,564) | 58.2 (48,73.8) | -26 (-29.1,-23) |
| Haiti | 6065 (5010,7657) | 109.2 (91.1,135.3) | 9045 (7575,11079) | 75.1 (63.6,91.5) | -31.2 (-34.8,-28.3) |
| Jamaica | 1958 (1620,2477) | 89.6 (73.8,113.2) | 2150 (1774,2752) | 72 (59.4,92.3) | -19.6 (-21.9,-16.9) |
| Puerto Rico | 5134 (4302,6159) | 141.5 (118.5,169.4) | 2747 (2248,3577) | 71.7 (58.3,94.8) | -49.3 (-55.9,-43.3) |
| Saint Kitts and Nevis | 34 (28,42) | 86.9 (72.6,106.9) | 42 (34,54) | 63.6 (52.5,82.6) | -26.8 (-30,-22.2) |
| Saint Lucia | 108 (90,136) | 88.1 (74.2,109.4) | 127 (104,162) | 64 (52.3,82.3) | -27.4 (-30.5,-23.7) |
| Saint Vincent and the Grenadines | 76 (64,95) | 76.9 (64.3,94.9) | 77 (63,98) | 62.6 (51.5,80.4) | -18.6 (-21.9,-14.5) |
| Suriname | 333 (279,412) | 92.5 (77.5,114.9) | 354 (290,447) | 58.4 (47.9,73.6) | -36.9 (-39.6,-34) |
| Trinidad and Tobago | 867 (721,1101) | 76.1 (63.5,96.6) | 927 (764,1192) | 60.4 (49.6,77.5) | -20.7 (-23.7,-17.7) |
| United States Virgin Islands | 95 (79,117) | 91.2 (76,112) | 72 (59,91) | 72.1 (59.4,92.3) | -20.9 (-23.9,-16) |
| Central Latin America | 215879 (177410,276922) | 144.2 (119.5,183.2) | 251323 (207225,324139) | 96.7 (79.8,124.8) | -33 (-34.1,-31.5) |
| Colombia | 43745 (35689,56008) | 143.1 (117.4,181.8) | 47069 (37996,62689) | 91.5 (73.8,122.2) | -36 (-38.2,-33.4) |
| Costa Rica | 3732 (3014,4964) | 131.7 (106.9,174) | 5590 (4590,7301) | 111.1 (91.2,145.6) | -15.6 (-18.1,-11.8) |
| El Salvador | 6135 (4990,8166) | 128 (105.1,168.4) | 6332 (5004,8454) | 98 (77.5,130.6) | -23.5 (-27.1,-19.3) |
| Guatemala | 11937 (9807,15146) | 170.6 (140.8,214.5) | 19992 (16589,25297) | 132.6 (110.3,165.7) | -22.3 (-25.7,-19.3) |
| Honduras | 7424 (6093,9530) | 180.6 (148.7,228.7) | 11492 (9385,14952) | 118.3 (97.3,152.8) | -34.5 (-37.3,-31.5) |
| Mexico | 107796 (89111,138420) | 140 (116.7,178.2) | 118232 (97918,152098) | 88.6 (73.4,114.2) | -36.7 (-37.9,-35.4) |
| Nicaragua | 5240 (4218,6933) | 153.7 (124.5,201.6) | 6526 (5269,8402) | 99.2 (80.3,127.4) | -35.5 (-38.6,-32.7) |
| Panama | 3213 (2610,4190) | 141 (114.9,182.9) | 4423 (3592,5844) | 101.5 (82.4,134.1) | -28 (-31.7,-24.7) |
| Venezuela (Bolivarian Republic of) | 26657 (21506,34900) | 152.4 (123.6,197.9) | 31667 (25744,41990) | 115.1 (93.3,153) | -24.5 (-27.6,-21.6) |
| Tropical Latin America | 63630 (53002,79920) | 45.3 (38.3,56.8) | 68838 (58340,84920) | 28.3 (24.1,35.1) | -37.6 (-39,-35.8) |
| Brazil | 61832 (51482,77559) | 45.2 (38.1,56.6) | 66382 (56239,81883) | 28.1 (23.9,34.8) | -37.9 (-39.3,-36) |
| Paraguay | 1798 (1516,2249) | 51 (43.1,63.2) | 2456 (2084,3032) | 34.6 (29.5,42.6) | -32.1 (-35.1,-29.2) |
| North Africa and Middle East | 83708 (70126,103725) | 29 (24.4,36) | 92419 (76083,115411) | 15.1 (12.5,18.8) | -47.9 (-49.5,-46.2) |
| Afghanistan | 2163 (1787,2740) | 26.4 (21.9,33.9) | 3473 (2828,4344) | 14.7 (12.2,18.3) | -44.4 (-46.7,-41.9) |
| Algeria | 7295 (6096,8988) | 34.6 (29.2,42.8) | 6549 (5421,8148) | 15.1 (12.5,18.7) | -56.5 (-58.4,-54.4) |
| Bahrain | 137 (115,171) | 29 (24.4,36.2) | 313 (260,396) | 19 (15.9,24) | -34.3 (-36.8,-32) |
| Egypt | 9462 (7790,12153) | 19.9 (16.4,25.4) | 13283 (10756,16870) | 13.6 (11,17.3) | -31.5 (-34.4,-28.9) |
| Iran (Islamic Republic of) | 16893 (14303,21124) | 35.9 (30.5,44.5) | 11811 (9796,14462) | 12.9 (10.8,15.9) | -64.1 (-66.3,-61.8) |
| Iraq | 3110 (2553,3964) | 20.8 (17.1,26.5) | 5555 (4572,7039) | 14.3 (11.7,17.9) | -31.5 (-33.9,-28.7) |
| Jordan | 979 (816,1266) | 33.2 (27.9,42.7) | 1904 (1567,2434) | 15.9 (13.1,20.2) | -52.1 (-54.1,-49.4) |
| Kuwait | 424 (355,534) | 26.8 (22.5,33.5) | 885 (726,1112) | 17.2 (14.1,21.6) | -35.8 (-38.2,-33) |
| Lebanon | 752 (631,948) | 27.4 (23,34.4) | 961 (792,1215) | 16.3 (13.4,20.6) | -40.5 (-42.6,-38.4) |
| Libya | 1016 (848,1265) | 29.1 (24.5,36.3) | 1200 (999,1485) | 16.4 (13.7,20.3) | -43.7 (-45.8,-41.7) |
| Morocco | 5952 (4942,7433) | 26.6 (22.1,33.4) | 5746 (4709,7116) | 15.1 (12.4,18.7) | -43.3 (-45.9,-41.1) |
| Oman | 509 (426,646) | 30.2 (25.2,38.3) | 733 (598,938) | 15.4 (12.6,19.7) | -48.9 (-51.2,-46.6) |
| Palestine | 389 (321,502) | 24.6 (20.4,31.2) | 805 (650,1037) | 17.5 (14.2,22.6) | -29 (-31.7,-26.3) |
| Qatar | 108 (90,137) | 25.1 (21,31.6) | 531 (434,678) | 16.4 (13.4,20.7) | -34.8 (-37,-32.3) |
| Saudi Arabia | 3673 (3050,4619) | 27.2 (22.7,34.2) | 5807 (4729,7328) | 14.4 (11.7,18.1) | -47 (-49.7,-44.7) |
| Sudan | 6008 (5004,7417) | 36.2 (30.3,44.8) | 7212 (5984,9101) | 19.2 (16.1,24) | -47 (-50.8,-44.1) |
| Syrian Arab Republic | 2434 (2028,3046) | 23.7 (20,29.5) | 2096 (1726,2619) | 14.5 (12,18.1) | -38.7 (-41,-36.7) |
| Tunisia | 2247 (1870,2798) | 29.9 (24.9,36.9) | 2011 (1654,2530) | 15.8 (13,20) | -47.2 (-49.8,-44.6) |
| Turkey | 16434 (13734,20376) | 31.5 (26.5,38.8) | 14484 (11913,18247) | 16 (13.1,20.2) | -49.2 (-51.6,-46.7) |
| United Arab Emirates | 483 (402,608) | 27.5 (22.9,34.6) | 2153 (1783,2711) | 19 (15.7,24.1) | -30.9 (-32.8,-28.8) |
| Yemen | 3194 (2671,3920) | 30.6 (25.7,37.5) | 4823 (3985,6046) | 17.1 (14.2,21.2) | -44.1 (-47,-41.2) |
| South Asia | 135874 (112023,172159) | 14 (11.6,17.8) | 153581 (127053,194092) | 8.3 (6.9,10.4) | -40.7 (-42.8,-38.6) |
| Bangladesh | 11022 (9038,14090) | 12 (9.9,15.2) | 12473 (10146,16024) | 7.5 (6.2,9.7) | -37 (-40,-34) |
| Bhutan | 70 (57,91) | 12.7 (10.3,16.2) | 64 (52,83) | 8.2 (6.7,10.7) | -35.3 (-37.9,-32.3) |
| India | 106815 (88073,135824) | 13.9 (11.5,17.7) | 114516 (95231,144420) | 7.9 (6.6,10) | -42.8 (-45.2,-40.5) |
| Nepal | 3831 (3163,4800) | 23.2 (19.2,29.4) | 4102 (3372,5113) | 13.5 (11.2,16.8) | -41.7 (-43.6,-39.5) |
| Pakistan | 14135 (11647,18088) | 14.9 (12.4,18.8) | 22426 (18584,28247) | 10.5 (8.7,13.1) | -29.9 (-33.1,-26.8) |
| East Asia | 352799 (294980,428646) | 30.3 (25.5,36.5) | 375956 (311940,454221) | 20.9 (17.4,25.6) | -30.9 (-35.7,-26.7) |
| China | 342217 (285882,416409) | 30.4 (25.5,36.6) | 366532 (303833,442772) | 21.1 (17.6,25.9) | -30.6 (-35.4,-26.3) |
| Democratic People's Republic of Korea | 4223 (3537,5104) | 21.2 (17.8,25.5) | 4512 (3733,5408) | 14.9 (12.3,17.9) | -29.9 (-34.7,-24.6) |
| Taiwan (Province of China) | 6359 (5306,7332) | 32.5 (27.1,37.4) | 4912 (4061,6040) | 16.7 (13.8,21.1) | -48.5 (-54.6,-42.3) |
| Oceania | 1333 (1124,1646) | 24.4 (20.5,30.3) | 2631 (2231,3272) | 21.4 (18.1,26.5) | -12.1 (-14,-10.1) |
| American Samoa | 10 (9,13) | 24.6 (20.6,30.6) | 9 (8,12) | 18.6 (15.6,23.4) | -24.2 (-26.4,-22.3) |
| Cook Islands | 5 (4,6) | 25.8 (21.6,32.2) | 4 (3,5) | 20.1 (16.7,25.8) | -22 (-24.3,-19.2) |
| Fiji | 153 (128,188) | 22.4 (18.9,27.8) | 154 (130,193) | 16.8 (14.2,21.1) | -25.2 (-27.1,-23) |
| Guam | 33 (28,41) | 25.2 (21.4,31.5) | 34 (28,43) | 20 (16.7,25.2) | -20.7 (-23.1,-18.1) |
| Kiribati | 20 (16,25) | 30.3 (25.2,38.4) | 25 (21,32) | 22.3 (18.6,28.6) | -26.5 (-28.7,-24.3) |
| Marshall Islands | 8 (7,11) | 23.4 (19.6,30) | 9 (8,12) | 17 (14.2,21.7) | -27.3 (-29.4,-25) |
| Micronesia (Federated States of) | 21 (17,26) | 23.8 (19.7,30.2) | 17 (14,21) | 16.5 (13.8,21.2) | -30.4 (-33.2,-27.1) |
| Nauru | 2 (2,3) | 23.6 (19.7,30.1) | 2 (1,2) | 16.2 (13.5,20.5) | -31.4 (-33.6,-29.2) |
| Niue | 1 (0,1) | 23.9 (20.1,29.9) | 0 (0,0) | 17.6 (14.6,22.4) | -26.3 (-28.4,-24.2) |
| Northern Mariana Islands | 12 (10,15) | 26.8 (22.5,33.8) | 11 (9,13) | 20.4 (17.1,25.9) | -23.9 (-26.3,-21.8) |
| Palau | 7 (6,9) | 48.8 (40.6,61.8) | 7 (6,9) | 35.3 (29.7,46) | -27.5 (-32.1,-24.7) |
| Papua New Guinea | 819 (691,1009) | 24.3 (20.5,30.3) | 1970 (1668,2438) | 22 (18.5,27) | -9.8 (-12.2,-7.5) |
| Samoa | 33 (28,41) | 23.3 (19.5,28.9) | 33 (28,42) | 17.3 (14.6,21.8) | -25.7 (-28.4,-22.4) |
| Solomon Islands | 70 (59,86) | 26.1 (22,32.3) | 165 (138,209) | 28 (23.4,35.2) | 7.1 (3.3,11.2) |
| Tokelau | 0 (0,0) | 25.5 (21.4,31.2) | 0 (0,0) | 17.5 (14.8,22) | -31.3 (-33.6,-28.8) |
| Tonga | 25 (21,31) | 29.8 (25.1,37.6) | 23 (19,28) | 23.7 (20,29.7) | -20.4 (-23.3,-17.7) |
| Tuvalu | 2 (2,2) | 23 (19,29) | 2 (2,2) | 14.9 (12.5,19) | -35.3 (-37.7,-32.5) |
| Vanuatu | 28 (23,36) | 22.8 (19.1,28.8) | 48 (40,61) | 17.1 (14.4,21.6) | -25.1 (-27.1,-22.8) |
| Southeast Asia | 116729 (98065,148003) | 28.6 (24,35.9) | 136059 (114283,170657) | 18.7 (15.7,23.4) | -34.5 (-35.8,-33.5) |
| Cambodia | 1913 (1583,2403) | 23.2 (19.3,29.3) | 2405 (1988,2974) | 14.6 (12.1,18) | -37.1 (-39.4,-34.7) |
| Indonesia | 40414 (33853,51497) | 24.8 (20.9,31.5) | 52083 (43708,65762) | 17.8 (15,22.5) | -28.2 (-29.3,-27) |
| Lao People's Democratic Republic | 979 (819,1216) | 28.7 (24,35.6) | 1074 (884,1331) | 15.5 (12.7,19.1) | -46.1 (-48.6,-43.9) |
| Malaysia | 5046 (4273,6207) | 33.3 (28.3,40.7) | 7299 (6139,8907) | 22.3 (18.8,27.2) | -33.2 (-35.4,-31) |
| Maldives | 45 (38,58) | 27.1 (22.9,34.2) | 125 (102,159) | 22 (18.1,28.1) | -18.9 (-21.6,-16.6) |
| Mauritius | 250 (209,316) | 23.6 (19.9,29.8) | 263 (217,331) | 17.9 (14.8,22.7) | -24 (-26.6,-21.3) |
| Myanmar | 19541 (16358,24452) | 54.3 (45.4,67.7) | 17699 (14802,22315) | 31.4 (26.3,39.5) | -42.2 (-44.6,-39.6) |
| Philippines | 13903 (11673,17794) | 26.6 (22.3,33.8) | 20874 (17448,26641) | 19.2 (16.1,24.4) | -27.8 (-29.1,-26.4) |
| Seychelles | 20 (17,25) | 30.1 (25.5,37.2) | 24 (20,30) | 21.3 (17.9,26.6) | -29.2 (-31.1,-27.3) |
| Sri Lanka | 5107 (4310,6393) | 32.3 (27.3,40.1) | 4174 (3469,5269) | 17.5 (14.5,22.1) | -45.9 (-48.9,-43.2) |
| Thailand | 11304 (9417,14514) | 20.8 (17.4,26.4) | 11182 (9290,13940) | 14 (11.7,17.7) | -32.5 (-34.8,-29.7) |
| Timor-Leste | 159 (131,200) | 25.1 (20.8,31.6) | 189 (156,239) | 15.8 (13.1,19.8) | -37.2 (-40,-34.2) |
| Viet Nam | 17878 (14938,22069) | 31 (25.9,38) | 18479 (15433,22763) | 17.4 (14.5,21.4) | -44 (-46.4,-41.4) |
| Central Sub-Saharan Africa | 12045 (9902,15258) | 26.8 (22.3,33.4) | 17639 (14649,22089) | 15.5 (13,19.3) | -42 (-45,-39.3) |
| Angola | 2650 (2178,3322) | 31.6 (26.4,39.5) | 4213 (3486,5247) | 16.3 (13.7,20.3) | -48.4 (-51.3,-45.7) |
| Central African Republic | 565 (466,708) | 25 (20.8,31.2) | 947 (784,1176) | 20.1 (16.8,25.1) | -19.7 (-22.4,-17.3) |
| Congo | 500 (408,639) | 24.8 (20.6,31.5) | 685 (565,861) | 14.1 (11.7,17.7) | -43.3 (-45.9,-40.9) |
| Democratic Republic of the Congo | 8004 (6578,10192) | 25.7 (21.4,32.1) | 11374 (9431,14250) | 15.1 (12.6,18.8) | -41.1 (-44.7,-38) |
| Equatorial Guinea | 102 (84,128) | 29.7 (24.6,37.3) | 169 (137,215) | 13 (10.7,16.5) | -56.2 (-58.8,-53.5) |
| Gabon | 223 (186,281) | 26.4 (22.1,33.1) | 251 (207,316) | 15.3 (12.7,19.1) | -42 (-45.1,-39.5) |
| Eastern Sub-Saharan Africa | 60317 (49762,76070) | 39 (32.5,49.2) | 79405 (65391,99891) | 22.1 (18.5,27.6) | -43.4 (-45.6,-41.4) |
| Burundi | 1747 (1432,2187) | 38.4 (31.7,48.4) | 2645 (2184,3241) | 24.1 (20,29.8) | -37.2 (-40,-34.7) |
| Comoros | 132 (110,163) | 35 (29.3,43.5) | 166 (137,208) | 23.6 (19.7,29.5) | -32.4 (-35.5,-29.5) |
| Djibouti | 120 (98,150) | 33.8 (28.1,42.5) | 281 (232,355) | 23.9 (19.9,29.9) | -29.3 (-31.7,-26.9) |
| Eritrea | 985 (809,1235) | 35 (29.2,44.5) | 1483 (1211,1887) | 25.4 (20.9,32.1) | -27.5 (-30.1,-24.4) |
| Ethiopia | 21973 (18315,27956) | 55.2 (46.5,69.2) | 20521 (17273,25771) | 22.8 (19.3,28.4) | -58.8 (-60.9,-57) |
| Kenya | 4845 (3962,6134) | 26.8 (22.2,33.6) | 8028 (6682,10139) | 18.5 (15.5,23.2) | -31.1 (-33.4,-29.2) |
| Madagascar | 3814 (3124,4740) | 38.7 (31.8,48.6) | 5082 (4173,6401) | 21 (17.3,26.6) | -45.6 (-48.2,-43) |
| Malawi | 2879 (2344,3662) | 35 (28.7,44.8) | 3339 (2715,4171) | 19.9 (16.4,25.2) | -43.1 (-46.6,-39.4) |
| Mozambique | 3659 (2975,4625) | 32.2 (26.4,40.6) | 4955 (4066,6213) | 19.6 (16.3,24.6) | -39.1 (-43.5,-34.9) |
| Rwanda | 2194 (1797,2769) | 37.1 (30.6,47.2) | 2512 (2066,3139) | 21.1 (17.4,26.6) | -43.2 (-45.9,-40.2) |
| Somalia | 2148 (1739,2714) | 33.7 (27.7,42.6) | 4592 (3776,5745) | 26.6 (22.1,33.2) | -21 (-23.9,-17.9) |
| South Sudan | 1745 (1423,2222) | 35.1 (29,44.2) | 1879 (1529,2366) | 22.9 (18.9,29) | -34.8 (-37.7,-32.1) |
| Uganda | 4109 (3309,5278) | 29.4 (23.8,37.8) | 7358 (6008,9145) | 20.6 (16.9,25.8) | -30.1 (-34.1,-26.9) |
| United Republic of Tanzania | 7612 (6213,9602) | 35.4 (29.3,44.7) | 13162 (10664,16583) | 25.9 (21.2,32.7) | -26.8 (-29.3,-24) |
| Zambia | 2313 (1890,2961) | 35.1 (29,44.3) | 3334 (2741,4210) | 20.2 (16.8,25.5) | -42.3 (-46.4,-38.6) |
| Southern Sub-Saharan Africa | 12296 (10222,15530) | 26.4 (22.1,33.6) | 12911 (10741,16225) | 16.2 (13.5,20.4) | -38.4 (-39.8,-37) |
| Botswana | 271 (222,345) | 24.3 (20,30.6) | 554 (452,693) | 23.3 (19.1,29.3) | -3.8 (-8.1,0.9) |
| Eswatini | 172 (139,218) | 25.3 (20.7,32) | 216 (179,269) | 20 (16.6,25) | -21.1 (-24.8,-18) |
| Lesotho | 288 (237,364) | 21.6 (17.7,27) | 373 (310,464) | 20.8 (17.3,25.8) | -3.6 (-6.6,-0.6) |
| Namibia | 303 (248,388) | 25 (20.7,31.7) | 412 (343,515) | 18 (15,22.3) | -28 (-32.5,-25.1) |
| South Africa | 9075 (7568,11474) | 26.9 (22.6,34.3) | 8283 (6891,10444) | 14.4 (11.9,18.1) | -46.7 (-48.6,-44.9) |
| Zimbabwe | 2187 (1797,2813) | 25.3 (21.1,32.1) | 3072 (2555,3826) | 22.4 (18.8,27.9) | -11.5 (-15.8,-7.8) |
| Western Sub-Saharan Africa | 47916 (39056,60520) | 29.7 (24.4,37.3) | 80493 (65840,99892) | 19.5 (16.1,24.3) | -34.5 (-36.3,-32.6) |
| Benin | 1224 (995,1544) | 31.1 (25.5,38.8) | 2641 (2164,3291) | 23.2 (19,28.5) | -25.4 (-27.8,-23) |
| Burkina Faso | 2405 (1953,3062) | 30.6 (25.1,38.6) | 4775 (3920,6065) | 25 (20.7,31.3) | -18.4 (-20.8,-15.6) |
| Cabo Verde | 85 (68,112) | 27.9 (22.5,36.3) | 107 (86,137) | 18.7 (15.1,24) | -33 (-37.1,-29.2) |
| Cameroon | 2182 (1760,2694) | 25.4 (20.6,31.7) | 5216 (4247,6558) | 19.1 (15.7,23.9) | -24.8 (-26.9,-22.2) |
| Chad | 1399 (1132,1758) | 28.3 (23.1,35.3) | 3424 (2799,4300) | 24.5 (20.2,30.6) | -13.6 (-16.2,-10.1) |
| C么te d'Ivoire | 2761 (2240,3462) | 27.5 (22.5,34.3) | 5047 (4074,6361) | 20.9 (17,26.3) | -24.2 (-26.6,-21.9) |
| Gambia | 244 (197,307) | 30.2 (24.7,38.1) | 395 (322,498) | 19.4 (16,24.3) | -35.8 (-39.1,-32.8) |
| Ghana | 3512 (2865,4367) | 27.8 (22.8,34.6) | 5963 (4882,7472) | 19.4 (16,24.3) | -30.1 (-33.2,-26.9) |
| Guinea | 1691 (1380,2149) | 33.2 (27.2,41.9) | 2854 (2332,3565) | 25 (20.7,31.1) | -24.6 (-27.2,-21.6) |
| Guinea-Bissau | 270 (218,344) | 32.3 (26.4,40.7) | 334 (273,417) | 19.2 (16,24) | -40.5 (-44.1,-37.4) |
| Liberia | 641 (522,813) | 30.9 (25.5,39.3) | 791 (645,994) | 16.7 (13.8,21) | -45.9 (-49.9,-42) |
| Mali | 2517 (2046,3204) | 35 (28.6,44.1) | 5162 (4191,6523) | 26.1 (21.3,32.5) | -25.4 (-27.9,-20.7) |
| Mauritania | 447 (366,554) | 26.1 (21.5,32.4) | 629 (516,791) | 16.8 (13.8,21) | -35.8 (-38.5,-33.3) |
| Niger | 2114 (1697,2672) | 32.7 (26.6,41) | 4585 (3717,5759) | 23.1 (18.9,28.3) | -29.2 (-33,-25.4) |
| Nigeria | 22359 (18169,28328) | 29.5 (24.1,37.7) | 32745 (27035,41209) | 17 (14.1,21.2) | -42.3 (-44.9,-40.4) |
| Sao Tome and Principe | 33 (27,42) | 31.7 (25.9,39.7) | 37 (30,47) | 18.6 (15.2,23.7) | -41.3 (-44.3,-38.1) |
| Senegal | 2119 (1719,2687) | 33.6 (27.6,42.1) | 2796 (2299,3516) | 20.3 (16.9,25.4) | -39.6 (-43.8,-35.8) |
| Sierra Leone | 1080 (873,1372) | 30.3 (24.7,38.1) | 1698 (1373,2111) | 21.7 (17.8,26.7) | -28.2 (-30.9,-25.7) |
| Togo | 832 (672,1050) | 27.7 (22.5,34.9) | 1295 (1056,1628) | 17.5 (14.4,21.9) | -36.9 (-39.5,-34.2) |

| **Table S2: Death due to non-CO poisoning in 1990 and 2021 and the percentage change in the age-standardized death rate (ASDR) per 100,000, by location** | | | | | |
| --- | --- | --- | --- | --- | --- |
|  | Death_Number_1990.95._UI. | ASDR_1990_per_100000.95._UI. | Death_Number_2021.95._UI. | ASDR_2021_per_100000.95._UI. | Percentage_change_in_ASDR_from_1990_to_2021 |
| Global | 29075.95035 (24656.91997,39796.22088) | 0.56463 (0.48361,0.78008) | 27262.50651 (16621.16596,33236.51698) | 0.34513 (0.21061,0.42511) | -38.9 (-62.7,-20.5) |
| Central Asia | 133.96854 (111.44982,162.56111) | 0.20391 (0.16706,0.25393) | 40.68352 (32.97183,52.92344) | 0.04425 (0.03616,0.0572) | -78.3 (-83.8,-70.6) |
| Armenia | 10.05295 (7.54933,13.16542) | 0.31022 (0.23269,0.40825) | 3.57279 (2.60731,4.67142) | 0.09881 (0.07256,0.12847) | -68.1 (-80.4,-52.4) |
| Azerbaijan | 12.47571 (8.13702,25.9246) | 0.17938 (0.11867,0.37253) | 9.11029 (4.75306,17.64753) | 0.08883 (0.04683,0.16892) | -50.5 (-71.3,-9) |
| Georgia | 7.70161 (5.77008,10.8151) | 0.13618 (0.10264,0.18824) | 3.79409 (2.95868,4.83891) | 0.08393 (0.0658,0.10543) | -38.4 (-58.5,-11.6) |
| Kazakhstan | 64.67314 (53.25065,83.07555) | 0.41171 (0.33856,0.5357) | 9.11742 (6.8019,11.85213) | 0.04886 (0.03653,0.0636) | -88.1 (-92,-83.3) |
| Kyrgyzstan | 3.45732 (2.69325,4.57444) | 0.08052 (0.06114,0.10999) | 2.09118 (1.56209,2.72271) | 0.03294 (0.02484,0.04243) | -59.1 (-75.3,-34.9) |
| Mongolia | 11.27734 (3.64344,20.72808) | 0.56389 (0.1865,1.00011) | 2.24549 (1.32261,4.73141) | 0.06978 (0.04132,0.14844) | -87.6 (-94.6,-63.9) |
| Tajikistan | 1.8033 (0.79907,6.0814) | 0.03021 (0.01524,0.09125) | 1.62689 (0.72451,3.9251) | 0.01662 (0.00794,0.04014) | -45 (-77,35.6) |
| Turkmenistan | 6.11346 (3.09574,7.79599) | 0.16625 (0.09344,0.23595) | 1.54141 (0.98571,2.44878) | 0.03072 (0.01994,0.04864) | -81.5 (-87.7,-71.6) |
| Uzbekistan | 16.4137 (10.13271,29.35711) | 0.0763 (0.04592,0.14873) | 7.58397 (5.21884,10.17395) | 0.02266 (0.01572,0.03014) | -70.3 (-85,-44.4) |
| Central Europe | 989.98439 (923.6739,1080.78823) | 0.75969 (0.70634,0.82703) | 70.08209 (55.31707,110.46077) | 0.0413 (0.0326,0.06474) | -94.6 (-95.7,-91.3) |
| Albania | 15.64133 (10.22472,20.43414) | 0.52199 (0.34604,0.67171) | 3.28606 (1.98895,6.55044) | 0.10741 (0.06742,0.21275) | -79.4 (-87.9,-55.9) |
| Bosnia and Herzegovina | 98.3054 (58.94517,144.32244) | 2.26225 (1.33812,3.34087) | 29.38916 (18.18503,55.3682) | 0.59349 (0.36373,1.11596) | -73.8 (-85.6,-48.7) |
| Bulgaria | 100.64138 (89.3747,112.99321) | 1.09655 (0.98041,1.21908) | 4.33533 (3.13161,5.88231) | 0.04291 (0.0306,0.0583) | -96.1 (-97.2,-94.4) |
| Croatia | 17.5186 (15.62046,19.53861) | 0.32812 (0.29447,0.36366) | 0.31871 (0.24687,0.41682) | 0.00458 (0.00368,0.00579) | -98.6 (-98.9,-98.2) |
| Czechia | 74.06391 (64.78237,84.37664) | 0.62143 (0.54889,0.70048) | 1.83549 (1.35948,2.38188) | 0.01047 (0.00809,0.01327) | -98.3 (-98.7,-97.8) |
| Hungary | 80.66366 (72.21786,88.74541) | 0.67834 (0.61199,0.74368) | 1.95387 (1.58369,2.42028) | 0.01352 (0.011,0.01666) | -98 (-98.4,-97.5) |
| Montenegro | 0.80275 (0.49646,1.27322) | 0.12928 (0.08027,0.20659) | 0.44336 (0.28686,1.00963) | 0.05694 (0.03716,0.12737) | -56 (-73.2,-31.6) |
| North Macedonia | 5.87813 (2.72384,7.9071) | 0.31073 (0.1442,0.41666) | 1.13615 (0.64582,3.24725) | 0.0441 (0.02528,0.12457) | -85.8 (-92.8,-48.1) |
| Poland | 377.09785 (365.243,388.92177) | 0.95283 (0.92197,0.98267) | 7.68667 (7.00196,8.44667) | 0.0144 (0.01314,0.01571) | -98.5 (-98.6,-98.4) |
| Romania | 165.61524 (130.96217,215.03679) | 0.68941 (0.54824,0.87001) | 12.23467 (9.29993,15.70988) | 0.04466 (0.03365,0.0576) | -93.5 (-95.4,-90.6) |
| Serbia | 17.44967 (9.23458,25.6292) | 0.18416 (0.09797,0.26767) | 4.13039 (2.6806,11.36177) | 0.03009 (0.01981,0.07953) | -83.7 (-91.4,-57.8) |
| Slovakia | 14.11591 (5.35957,18.78469) | 0.25553 (0.09676,0.33905) | 2.15956 (1.19155,7.32546) | 0.02865 (0.01595,0.09505) | -88.8 (-94.7,-56.1) |
| Slovenia | 6.35885 (5.85109,6.89336) | 0.28848 (0.26628,0.31348) | 0.1526 (0.11994,0.19071) | 0.00424 (0.00341,0.00521) | -98.5 (-98.8,-98.2) |
| Eastern Europe | 198.51043 (160.14428,278.2473) | 0.0852 (0.06943,0.11561) | 81.85491 (75.24471,89.29351) | 0.03453 (0.0319,0.03748) | -59.5 (-71.2,-48.2) |
| Belarus | 40.6136 (30.81482,58.03572) | 0.36868 (0.28461,0.49913) | 5.67369 (4.07826,7.42817) | 0.04789 (0.03457,0.06211) | -87 (-91.7,-79.7) |
| Estonia | 5.00677 (3.82001,7.18745) | 0.30086 (0.2327,0.42472) | 0.25233 (0.19016,0.32137) | 0.01449 (0.01107,0.01823) | -95.2 (-96.9,-92.9) |
| Latvia | 4.38363 (3.10393,6.56579) | 0.15509 (0.11111,0.22952) | 0.80901 (0.63796,1.01879) | 0.0322 (0.02546,0.04056) | -79.2 (-86.3,-69) |
| Lithuania | 17.10694 (13.0227,24.39109) | 0.44167 (0.33643,0.6228) | 1.00385 (0.79447,1.268) | 0.028 (0.02202,0.03531) | -93.7 (-95.7,-90.6) |
| Republic of Moldova | 12.95949 (9.16073,18.89412) | 0.29214 (0.20761,0.42181) | 2.38455 (1.85292,3.04696) | 0.05522 (0.04318,0.07041) | -81.1 (-87.7,-71.7) |
| Russian Federation | 92.14166 (72.87975,138.29187) | 0.05976 (0.04773,0.08781) | 62.98507 (57.96897,68.43702) | 0.03857 (0.03572,0.04159) | -35.5 (-58.2,-14.6) |
| Ukraine | 26.29834 (20.78504,34.64577) | 0.04816 (0.03841,0.06279) | 8.74641 (6.15831,11.69193) | 0.0174 (0.01236,0.02268) | -63.9 (-77.6,-45.7) |
| Australasia | 25.98681 (24.57796,27.43808) | 0.12154 (0.115,0.12824) | 9.76857 (8.79246,10.6117) | 0.0244 (0.02213,0.02669) | -79.9 (-82,-77.8) |
| Australia | 23.1865 (21.78096,24.63565) | 0.13027 (0.12264,0.13816) | 9.51936 (8.55981,10.36429) | 0.02827 (0.02565,0.03094) | -78.3 (-80.5,-75.8) |
| New Zealand | 2.8003 (2.64151,2.97014) | 0.07887 (0.07421,0.08354) | 0.24921 (0.2223,0.2808) | 0.00451 (0.00405,0.00507) | -94.3 (-94.9,-93.5) |
| High-income Asia Pacific | 604.44965 (504.21668,720.21398) | 0.31355 (0.26209,0.37603) | 165.15116 (137.76579,245.37719) | 0.04194 (0.03535,0.06803) | -86.6 (-89.1,-77.9) |
| Brunei Darussalam | 0.42652 (0.26571,0.5795) | 0.23064 (0.14736,0.30482) | 0.34499 (0.25267,0.55054) | 0.07932 (0.05836,0.13067) | -65.6 (-76.2,-40.1) |
| Japan | 279.48998 (265.80805,286.78771) | 0.18217 (0.17324,0.18696) | 83.16667 (71.53146,89.89377) | 0.02682 (0.02467,0.02836) | -85.3 (-86,-84.6) |
| Republic of Korea | 320.87173 (222.32879,437.64703) | 0.93593 (0.6205,1.25951) | 81.11089 (61.0976,158.63583) | 0.09624 (0.0717,0.19523) | -89.7 (-92.9,-77.2) |
| Singapore | 3.66142 (3.42383,3.88673) | 0.13386 (0.12575,0.1414) | 0.52861 (0.47409,0.58912) | 0.00666 (0.00602,0.00736) | -95 (-95.5,-94.4) |
| High-income North America | 590.5023 (574.27164,607.66314) | 0.19519 (0.19011,0.20086) | 204.79796 (195.13838,212.97161) | 0.04851 (0.04655,0.05046) | -75.1 (-76.2,-74.1) |
| Canada | 87.61824 (80.62586,94.99915) | 0.29738 (0.27434,0.32146) | 21.41752 (18.97854,24.04137) | 0.04661 (0.04123,0.05233) | -84.3 (-86.4,-81.9) |
| Greenland | 0.11843 (0.02628,0.16192) | 0.22087 (0.04922,0.29948) | 0.01766 (0.0082,0.06683) | 0.02728 (0.01275,0.11004) | -87.6 (-94.3,-49.5) |
| United States of America | 502.75209 (489.37315,517.71671) | 0.18405 (0.17937,0.18927) | 183.35957 (174.40504,190.85541) | 0.0487 (0.04678,0.05084) | -73.5 (-74.6,-72.3) |
| Southern Latin America | 280.52266 (263.39649,298.48396) | 0.58527 (0.54979,0.62277) | 36.55736 (30.85255,42.81607) | 0.04882 (0.04135,0.05758) | -91.7 (-93.1,-90) |
| Argentina | 203.54052 (187.9503,219.04523) | 0.63004 (0.58209,0.6787) | 26.31806 (22.09276,31.42649) | 0.05394 (0.0451,0.06402) | -91.4 (-93,-89.6) |
| Chile | 55.06009 (52.2813,58.20493) | 0.44852 (0.42675,0.47353) | 6.11823 (5.17341,7.25178) | 0.02808 (0.02392,0.0333) | -93.7 (-94.8,-92.5) |
| Uruguay | 21.90873 (20.24318,23.23638) | 0.66587 (0.61405,0.70646) | 4.11905 (3.4931,4.81104) | 0.09267 (0.07906,0.10859) | -86.1 (-88.4,-83.4) |
| Western Europe | 797.03402 (770.54423,819.53583) | 0.17902 (0.17326,0.18387) | 129.68749 (120.68248,136.81568) | 0.02032 (0.01939,0.02113) | -88.7 (-89.1,-88.1) |
| Andorra | 0.00367 (0.00243,0.00534) | 0.00631 (0.00427,0.00919) | 0.00294 (0.00193,0.0042) | 0.00257 (0.00168,0.00364) | -59.2 (-74.5,-38.7) |
| Austria | 15.13019 (14.19374,16.13553) | 0.16675 (0.15728,0.17845) | 2.83158 (2.60042,3.09401) | 0.02404 (0.02234,0.02599) | -85.6 (-87,-84) |
| Belgium | 15.50891 (14.16065,16.85183) | 0.13244 (0.12073,0.14447) | 1.97098 (1.73057,2.22464) | 0.012 (0.0107,0.01335) | -90.9 (-92.2,-89.6) |
| Cyprus | 0.8041 (0.48316,1.41065) | 0.1116 (0.06598,0.20308) | 0.42148 (0.27521,0.79862) | 0.02503 (0.01623,0.04677) | -77.6 (-87.8,-62.4) |
| Denmark | 6.70042 (6.3682,7.077) | 0.10691 (0.10167,0.11256) | 0.70507 (0.63641,0.76739) | 0.00796 (0.00731,0.00858) | -92.6 (-93.2,-91.8) |
| Finland | 7.90203 (6.24258,8.98907) | 0.13511 (0.10498,0.15412) | 1.02239 (0.9135,1.12992) | 0.01239 (0.01127,0.01348) | -90.8 (-92.2,-87.9) |
| France | 95.45327 (89.03113,102.37578) | 0.14158 (0.13218,0.1515) | 20.61497 (17.95565,23.0047) | 0.01746 (0.01591,0.01925) | -87.7 (-89,-86.2) |
| Germany | 52.12182 (39.08636,61.88545) | 0.0563 (0.04163,0.06656) | 9.26341 (7.85628,11.00215) | 0.00687 (0.00589,0.00813) | -87.8 (-90.4,-83) |
| Greece | 22.7811 (21.53678,24.16405) | 0.1904 (0.18029,0.20172) | 4.87067 (4.38429,5.36653) | 0.03003 (0.02705,0.033) | -84.2 (-85.9,-82.4) |
| Iceland | 0.45686 (0.41582,0.4939) | 0.17129 (0.15626,0.18597) | 0.10864 (0.0912,0.12598) | 0.02381 (0.02038,0.02743) | -86.1 (-88.4,-83.3) |
| Ireland | 9.05207 (8.5578,9.59041) | 0.2468 (0.23369,0.26051) | 1.30493 (1.16493,1.43964) | 0.02058 (0.01876,0.02256) | -91.7 (-92.5,-90.8) |
| Israel | 8.05351 (7.61921,8.50637) | 0.16952 (0.16044,0.17889) | 2.26215 (2.03778,2.4613) | 0.02105 (0.01918,0.02285) | -87.6 (-88.7,-86.4) |
| Italy | 173.62435 (166.98642,179.24418) | 0.2504 (0.24161,0.25791) | 12.88397 (11.18932,13.97939) | 0.01087 (0.00995,0.01159) | -95.7 (-96,-95.4) |
| Luxembourg | 0.73186 (0.67506,0.78791) | 0.16474 (0.15267,0.17716) | 0.11723 (0.10235,0.13282) | 0.01285 (0.01135,0.01444) | -92.2 (-93.2,-91.1) |
| Malta | 0.29547 (0.27001,0.32146) | 0.07545 (0.06845,0.08214) | 0.04714 (0.04141,0.05396) | 0.00751 (0.00668,0.00849) | -90 (-91.4,-88.7) |
| Monaco | 0.52125 (0.26763,0.68398) | 1.34639 (0.69382,1.81188) | 0.27793 (0.203,0.42213) | 0.57662 (0.40254,0.90849) | -57.2 (-74.1,-13.8) |
| Netherlands | 6.71772 (6.29015,7.22355) | 0.03934 (0.0369,0.04206) | 1.1166 (1.00558,1.23426) | 0.0044 (0.00402,0.00482) | -88.8 (-90,-87.5) |
| Norway | 3.85414 (3.70959,3.98579) | 0.07862 (0.07611,0.08124) | 1.68225 (1.5241,1.78957) | 0.02024 (0.0188,0.02144) | -74.3 (-75.9,-72.6) |
| Portugal | 80.3129 (74.89182,85.86601) | 0.75002 (0.70014,0.80131) | 12.27309 (11.04106,13.43807) | 0.07157 (0.06521,0.07816) | -90.5 (-91.4,-89.3) |
| San Marino | 0.15955 (0.11928,0.1962) | 0.57533 (0.43536,0.70391) | 0.08775 (0.05685,0.13445) | 0.18329 (0.11451,0.2883) | -68.1 (-80.6,-50.3) |
| Spain | 79.30301 (74.20488,85.02147) | 0.18348 (0.17158,0.1959) | 13.81712 (12.47229,15.37906) | 0.01887 (0.01745,0.02057) | -89.7 (-90.7,-88.4) |
| Sweden | 75.28172 (71.87559,78.46259) | 0.74608 (0.71214,0.77802) | 12.25308 (10.93361,13.75767) | 0.09549 (0.08538,0.10731) | -87.2 (-88.6,-85.5) |
| Switzerland | 1.87392 (1.69407,2.07971) | 0.02294 (0.02065,0.02537) | 0.13002 (0.11534,0.14384) | 0.00095 (0.00086,0.00104) | -95.9 (-96.4,-95.3) |
| United Kingdom | 139.7351 (137.52182,141.94189) | 0.21845 (0.21537,0.22178) | 29.50789 (28.56251,30.35313) | 0.03681 (0.03578,0.0378) | -83.1 (-83.6,-82.6) |
| Andean Latin America | 475.09481 (398.28651,542.27245) | 1.28311 (1.07397,1.46421) | 204.84683 (161.24898,281.55144) | 0.31377 (0.24746,0.42986) | -75.5 (-81.2,-65.1) |
| Bolivia (Plurinational State of) | 99.64847 (74.95045,132.45353) | 1.55042 (1.19464,2.07484) | 51.83408 (34.15884,85.57653) | 0.45969 (0.30495,0.75006) | -70.4 (-80.7,-51.7) |
| Ecuador | 167.55244 (151.17379,183.25963) | 1.80632 (1.62547,1.97267) | 55.41106 (44.28967,67.45836) | 0.31207 (0.24964,0.37912) | -82.7 (-86.5,-78.7) |
| Peru | 207.8939 (144.48939,254.95835) | 0.96371 (0.67027,1.16964) | 97.60169 (71.37127,147.22479) | 0.26912 (0.19654,0.40544) | -72.1 (-81.3,-54.6) |
| Caribbean | 336.3635 (255.41039,426.49694) | 0.94903 (0.7414,1.16806) | 98.84004 (72.47526,157.66786) | 0.21936 (0.15815,0.35196) | -76.9 (-83.9,-60.3) |
| Antigua and Barbuda | 0.43597 (0.39424,0.48326) | 0.75163 (0.67965,0.82928) | 0.02941 (0.02663,0.03297) | 0.03372 (0.03038,0.03768) | -95.5 (-96,-94.9) |
| Bahamas | 1.858 (1.66058,2.08871) | 0.78457 (0.70403,0.87488) | 0.10095 (0.07918,0.13071) | 0.02631 (0.02048,0.03395) | -96.6 (-97.4,-95.6) |
| Barbados | 2.1042 (1.98342,2.23115) | 0.8 (0.75187,0.84991) | 0.33619 (0.26973,0.41907) | 0.09138 (0.07276,0.11511) | -88.6 (-91,-85.5) |
| Belize | 4.75662 (4.3441,5.22661) | 2.382 (2.20629,2.58028) | 0.28406 (0.24079,0.33161) | 0.07064 (0.06007,0.08223) | -97 (-97.5,-96.5) |
| Bermuda | 0.137 (0.09217,0.17337) | 0.21536 (0.14304,0.26957) | 0.00053 (0.00044,0.00065) | 0.00069 (0.00057,0.00083) | -99.7 (-99.8,-99.5) |
| Cuba | 38.53799 (34.96823,42.14704) | 0.36617 (0.33329,0.40143) | 4.3349 (3.64462,5.06019) | 0.02971 (0.02504,0.03454) | -91.9 (-93.3,-90.2) |
| Dominica | 2.20141 (1.08078,2.73666) | 3.23809 (1.60127,3.95997) | 0.51045 (0.33822,1.19974) | 0.78412 (0.5136,1.81137) | -75.8 (-85.8,-12.2) |
| Dominican Republic | 48.34247 (25.19736,64.64364) | 0.62052 (0.33059,0.80872) | 23.04308 (14.04709,31.31012) | 0.21165 (0.12913,0.28428) | -65.9 (-76.1,-51) |
| Grenada | 1.66984 (1.48704,1.85834) | 2.00839 (1.79174,2.22426) | 0.05753 (0.05033,0.06506) | 0.059 (0.05169,0.0665) | -97.1 (-97.4,-96.6) |
| Guyana | 9.00394 (7.74932,10.06574) | 1.23127 (1.07493,1.36261) | 0.33387 (0.25066,0.43224) | 0.04535 (0.034,0.05869) | -96.3 (-97.4,-95.2) |
| Haiti | 139.55867 (72.03026,223.97238) | 1.83925 (1.08277,2.64564) | 62.05991 (37.62429,120.10405) | 0.48674 (0.28788,0.92993) | -73.5 (-84.9,-44.5) |
| Jamaica | 4.25638 (3.88159,4.68164) | 0.18587 (0.17037,0.20273) | 1.79302 (1.37663,2.29726) | 0.06055 (0.04632,0.07753) | -67.4 (-74.6,-58.8) |
| Puerto Rico | 56.90948 (52.70953,61.0882) | 1.59453 (1.47753,1.70839) | 0.38786 (0.32113,0.45784) | 0.00757 (0.00631,0.00885) | -99.5 (-99.6,-99.4) |
| Saint Kitts and Nevis | 0.34626 (0.32332,0.37011) | 0.90315 (0.84311,0.96334) | 0.03339 (0.02754,0.04011) | 0.0653 (0.0539,0.07816) | -92.8 (-94,-91.2) |
| Saint Lucia | 1.11349 (1.01006,1.21952) | 0.9263 (0.85364,1.00499) | 0.06167 (0.05021,0.07432) | 0.03256 (0.02641,0.0393) | -96.5 (-97.2,-95.7) |
| Saint Vincent and the Grenadines | 0.7777 (0.70561,0.84903) | 0.78545 (0.71847,0.85225) | 0.0675 (0.0585,0.07735) | 0.05741 (0.04952,0.06618) | -92.7 (-93.9,-91.5) |
| Suriname | 3.74477 (1.46859,4.80307) | 1.01879 (0.41641,1.29318) | 1.12423 (0.76171,2.34796) | 0.19727 (0.13288,0.4131) | -80.6 (-88.4,-23.1) |
| Trinidad and Tobago | 9.0258 (8.56608,9.50523) | 0.82335 (0.78595,0.86502) | 0.90213 (0.70325,1.13445) | 0.06 (0.04691,0.07577) | -92.7 (-94.3,-90.9) |
| United States Virgin Islands | 0.19383 (0.07562,0.27078) | 0.20839 (0.08026,0.29363) | 0.03449 (0.02,0.07945) | 0.03492 (0.01948,0.08041) | -83.2 (-90.7,-56.5) |
| Central Latin America | 1097.4602 (1056.19917,1147.73927) | 0.73998 (0.71565,0.77234) | 554.44868 (480.27956,635.33522) | 0.21625 (0.18713,0.24789) | -70.8 (-74.8,-66.7) |
| Colombia | 149.06894 (141.9394,156.56598) | 0.50124 (0.4797,0.52312) | 40.48291 (34.04558,47.67092) | 0.07727 (0.06477,0.09105) | -84.6 (-87.1,-81.6) |
| Costa Rica | 9.13699 (8.59486,9.66967) | 0.37113 (0.34913,0.39385) | 10.28509 (9.08682,11.41918) | 0.19663 (0.17375,0.21824) | -47 (-54.2,-40) |
| El Salvador | 106.41108 (92.30864,128.36004) | 2.34668 (2.05129,2.85868) | 49.18567 (36.86493,68.20648) | 0.7648 (0.57177,1.05495) | -67.4 (-77.8,-56.2) |
| Guatemala | 187.65303 (177.57596,198.4137) | 2.49514 (2.39701,2.5975) | 130.28088 (112.8664,149.24024) | 0.86635 (0.74687,0.99543) | -65.3 (-70.5,-60.3) |
| Honduras | 58.36695 (47.41334,75.09902) | 1.40029 (1.13505,1.79984) | 56.11385 (38.56656,85.05912) | 0.6928 (0.48944,1.0251) | -50.5 (-66.4,-30.5) |
| Mexico | 470.0207 (450.09259,491.48538) | 0.6136 (0.59392,0.6336) | 175.12698 (155.74351,194.05624) | 0.13536 (0.12046,0.15001) | -77.9 (-80.5,-75.4) |
| Nicaragua | 21.82566 (14.20088,27.7982) | 0.59988 (0.38678,0.73555) | 12.4501 (9.85825,17.32451) | 0.20324 (0.16116,0.28059) | -66.1 (-74.8,-43.7) |
| Panama | 9.48291 (8.92751,10.03235) | 0.44076 (0.4179,0.46443) | 5.93577 (4.82285,7.0836) | 0.13619 (0.11082,0.16235) | -69.1 (-74.6,-63.3) |
| Venezuela (Bolivarian Republic of) | 85.49394 (77.36384,91.6801) | 0.52934 (0.47846,0.56748) | 74.58743 (56.51514,94.57673) | 0.27409 (0.20784,0.34596) | -48.2 (-61.7,-31.4) |
| Tropical Latin America | 203.63319 (191.84036,216.47376) | 0.14667 (0.13966,0.15433) | 87.09993 (82.3565,92.97194) | 0.03663 (0.03435,0.03929) | -75 (-76.8,-72.7) |
| Brazil | 191.60055 (181.04536,202.99795) | 0.14204 (0.13572,0.14919) | 77.86374 (74.40347,81.27754) | 0.03353 (0.03179,0.03506) | -76.4 (-77.7,-75) |
| Paraguay | 12.03264 (8.65467,14.93562) | 0.30268 (0.22368,0.37848) | 9.2362 (6.59748,14.73563) | 0.13878 (0.09923,0.22003) | -54.2 (-69,-21.9) |
| North Africa and Middle East | 2580.26156 (1372.55477,3567.41372) | 0.70163 (0.37869,1.01866) | 1298.89952 (691.00265,1826.62532) | 0.21686 (0.1164,0.30391) | -69.1 (-76.6,-58) |
| Afghanistan | 274.50035 (103.25834,470.34129) | 2.52352 (0.91031,4.63641) | 331.07804 (132.11254,570.49134) | 1.12487 (0.43189,1.89789) | -55.4 (-69.2,-31.4) |
| Algeria | 164.67236 (62.24237,259.84886) | 0.62694 (0.2369,1.01192) | 68.06077 (27.89199,103.25226) | 0.1637 (0.06637,0.24656) | -73.9 (-81.5,-57.5) |
| Bahrain | 0.43963 (0.32205,0.67617) | 0.10842 (0.07749,0.17503) | 0.40957 (0.25766,0.68579) | 0.03379 (0.02116,0.06163) | -68.8 (-78.5,-54.7) |
| Egypt | 308.7939 (222.51277,381.58708) | 0.54318 (0.41062,0.66589) | 167.2623 (120.50767,219.00949) | 0.18068 (0.13418,0.24261) | -66.7 (-75,-56.3) |
| Iran (Islamic Republic of) | 595.05148 (384.82931,851.56685) | 0.96608 (0.62233,1.43218) | 122.81828 (93.3459,241.82114) | 0.144 (0.10884,0.28838) | -85.1 (-91.8,-70.2) |
| Iraq | 49.13474 (33.95899,71.18581) | 0.25398 (0.17253,0.38798) | 30.28374 (20.03469,48.81771) | 0.08022 (0.05316,0.1346) | -68.4 (-80.1,-53.1) |
| Jordan | 17.49912 (11.25658,28.55339) | 0.47298 (0.30372,0.81468) | 11.99743 (7.77423,23.15113) | 0.10672 (0.06885,0.21849) | -77.4 (-85.6,-64.7) |
| Kuwait | 3.06324 (2.7396,3.45719) | 0.19199 (0.17241,0.21819) | 0.9855 (0.78392,1.24149) | 0.0245 (0.01975,0.03049) | -87.2 (-89.8,-84) |
| Lebanon | 7.29817 (5.19264,9.98352) | 0.26138 (0.18665,0.35807) | 4.62344 (3.4004,6.68273) | 0.07871 (0.05793,0.11288) | -69.9 (-78.3,-56.7) |
| Libya | 17.95488 (7.40582,27.75745) | 0.44396 (0.18874,0.67829) | 15.35425 (6.26848,29.40273) | 0.23334 (0.09637,0.45159) | -47.4 (-69.9,-6.6) |
| Morocco | 232.78056 (83.09965,399.08415) | 0.85686 (0.30146,1.50712) | 92.03761 (33.05025,157.41914) | 0.25484 (0.09174,0.43623) | -70.3 (-79.5,-53.5) |
| Oman | 1.27064 (0.73445,2.38411) | 0.06937 (0.03947,0.13722) | 0.49144 (0.32559,0.92345) | 0.01306 (0.00851,0.02518) | -81.2 (-91.7,-61.6) |
| Palestine | 4.3353 (2.96672,6.21421) | 0.2116 (0.14335,0.32517) | 2.59539 (1.82487,4.59836) | 0.05874 (0.04205,0.10716) | -72.2 (-83.2,-56) |
| Qatar | 0.3857 (0.20291,0.65193) | 0.11363 (0.06228,0.20228) | 0.4172 (0.23488,0.91053) | 0.01949 (0.01072,0.05032) | -82.9 (-90,-68.4) |
| Saudi Arabia | 63.6982 (42.45844,101.68233) | 0.40733 (0.2668,0.66127) | 49.61093 (26.7883,92.00682) | 0.12599 (0.07325,0.23277) | -69.1 (-83.2,-46.1) |
| Sudan | 437.47191 (156.19748,738.79757) | 1.80778 (0.65175,2.85156) | 203.6717 (86.2461,362.92475) | 0.49021 (0.20252,0.89625) | -72.9 (-83.6,-54.5) |
| Syrian Arab Republic | 26.43156 (15.81907,43.75541) | 0.18719 (0.11148,0.33379) | 7.82441 (4.34476,16.37075) | 0.05904 (0.03296,0.1238) | -68.5 (-82.3,-44) |
| Tunisia | 43.23847 (16.96433,70.98567) | 0.5098 (0.19122,0.83797) | 15.50486 (6.43798,24.1938) | 0.12884 (0.05393,0.19913) | -74.7 (-85.2,-58.2) |
| Turkey | 108.80562 (69.30734,214.35318) | 0.19482 (0.12932,0.36424) | 22.09757 (14.27094,55.09612) | 0.02721 (0.01754,0.06847) | -86 (-91.3,-78.1) |
| United Arab Emirates | 2.17394 (1.02859,3.89782) | 0.14544 (0.06908,0.26564) | 2.07877 (1.20943,3.12462) | 0.03486 (0.0201,0.06071) | -76 (-87,-49.5) |
| Yemen | 219.85028 (65.36363,397.3263) | 1.37302 (0.3934,2.81342) | 148.48484 (49.76577,296.14338) | 0.49265 (0.15244,0.97141) | -64.1 (-78.9,-32) |
| South Asia | 3431.83438 (1822.56116,4379.20882) | 0.31477 (0.17398,0.40736) | 1950.32873 (1139.9723,2577.86414) | 0.1154 (0.06909,0.15145) | -63.3 (-72,-48.6) |
| Bangladesh | 651.58627 (48.13229,1136.81433) | 0.41125 (0.03842,0.67976) | 127.63566 (18.57033,232.52175) | 0.07956 (0.01195,0.14822) | -80.7 (-89,-54.5) |
| Bhutan | 0.68191 (0.34059,1.68491) | 0.11485 (0.05436,0.28604) | 0.30021 (0.09904,0.95889) | 0.04422 (0.01484,0.14234) | -61.5 (-80.1,-30.9) |
| India | 1695.66686 (580.37865,2334.60156) | 0.20899 (0.07602,0.29387) | 1059.62058 (327.11736,1552.12797) | 0.07849 (0.02526,0.1144) | -62.4 (-72.7,-52.6) |
| Nepal | 959.00438 (659.50286,1431.61651) | 5.30839 (3.86148,7.46103) | 619.0795 (432.71943,938.17383) | 2.40627 (1.6747,3.48078) | -54.7 (-68.6,-33.6) |
| Pakistan | 124.89496 (67.4063,355.44745) | 0.11062 (0.05968,0.31095) | 143.69277 (71.95149,478.45524) | 0.06847 (0.03447,0.22499) | -38.1 (-65.2,-14.9) |
| East Asia | 5368.65608 (3157.61041,15746.12405) | 0.49048 (0.28752,1.45975) | 8319.07708 (3273.76578,11003.5026) | 0.45454 (0.17695,0.59525) | -7.3 (-78.8,95.8) |
| China | 4777.99525 (2614.6457,14961.32236) | 0.45089 (0.24478,1.43485) | 8069.59997 (3024.13869,10766.82239) | 0.45641 (0.16984,0.6049) | 1.2 (-79.3,132.2) |
| Democratic People's Republic of Korea | 91.68204 (38.15891,311.50598) | 0.47102 (0.19354,1.65068) | 168.93147 (83.5572,410.78689) | 0.55691 (0.26773,1.35415) | 18.2 (-40.3,141.9) |
| Taiwan (Province of China) | 498.97879 (483.22545,515.51457) | 2.6288 (2.54002,2.71895) | 80.54563 (73.04983,87.69589) | 0.24153 (0.22051,0.26126) | -90.8 (-91.6,-90.1) |
| Oceania | 37.62141 (21.34267,71.04046) | 0.59816 (0.34977,1.21811) | 50.15201 (30.93319,103.38505) | 0.37006 (0.22587,0.77818) | -38.1 (-55.1,-8.9) |
| American Samoa | 0.10475 (0.06437,0.13866) | 0.25154 (0.15671,0.33691) | 0.05782 (0.04112,0.09102) | 0.121 (0.08629,0.19017) | -51.9 (-70,-8.7) |
| Cook Islands | 0.02617 (0.01728,0.03692) | 0.14931 (0.09885,0.21016) | 0.00765 (0.0052,0.01139) | 0.03887 (0.02665,0.05852) | -74 (-85.1,-57.2) |
| Fiji | 2.14828 (1.54271,3.01822) | 0.30598 (0.22125,0.42773) | 1.30472 (0.92283,2.0347) | 0.14893 (0.10682,0.22917) | -51.3 (-68.8,-23.8) |
| Guam | 0.15315 (0.06762,0.22426) | 0.12316 (0.05544,0.17667) | 0.0557 (0.03996,0.10358) | 0.03409 (0.02469,0.06394) | -72.3 (-84.8,-24) |
| Kiribati | 0.82776 (0.49947,1.15275) | 1.09507 (0.65186,1.52635) | 0.83978 (0.45386,1.2985) | 0.72791 (0.41986,1.12449) | -33.5 (-56.8,-3.5) |
| Marshall Islands | 0.19116 (0.09889,0.31545) | 0.50287 (0.26349,0.82043) | 0.17077 (0.0831,0.30889) | 0.32284 (0.16091,0.57385) | -35.8 (-58.7,-2.4) |
| Micronesia (Federated States of) | 0.57096 (0.36189,0.90196) | 0.60943 (0.38892,0.98168) | 0.30548 (0.18432,0.53741) | 0.31523 (0.1953,0.54552) | -48.3 (-65.5,-16.7) |
| Nauru | 0.04716 (0.02666,0.07306) | 0.5316 (0.30335,0.83225) | 0.03525 (0.02137,0.06048) | 0.34846 (0.21228,0.59338) | -34.5 (-59,6.5) |
| Niue | 0.00784 (0.00509,0.01226) | 0.34206 (0.21947,0.54266) | 0.00441 (0.00285,0.00785) | 0.27889 (0.1822,0.49574) | -18.5 (-48.1,18.8) |
| Northern Mariana Islands | 0.07761 (0.03486,0.12577) | 0.18942 (0.08387,0.29374) | 0.03341 (0.02148,0.06895) | 0.06718 (0.04373,0.13423) | -64.5 (-80.3,-7.8) |
| Palau | 0.24891 (0.14989,0.37392) | 1.70819 (1.03328,2.57927) | 0.25612 (0.18917,0.35925) | 1.35004 (0.99715,1.8856) | -21 (-45.7,27.5) |
| Papua New Guinea | 26.0915 (14.05881,52.90112) | 0.64786 (0.32847,1.4617) | 39.65273 (22.8361,88.09436) | 0.38933 (0.21916,0.89128) | -39.9 (-60,-5.6) |
| Samoa | 0.60574 (0.39899,0.98268) | 0.39699 (0.26115,0.64699) | 0.40086 (0.25694,0.67476) | 0.20954 (0.1377,0.35253) | -47.2 (-63.5,-23.3) |
| Solomon Islands | 2.28235 (1.19267,4.90183) | 0.76916 (0.39204,1.65745) | 2.88999 (1.63674,5.404) | 0.46705 (0.26362,0.88508) | -39.3 (-61.2,13.9) |
| Tokelau | 0.00514 (0.00252,0.00923) | 0.34463 (0.16739,0.61613) | 0.00397 (0.00165,0.00848) | 0.29626 (0.1281,0.63184) | -14 (-44.2,27.4) |
| Tonga | 0.99708 (0.72148,1.43877) | 1.14596 (0.85277,1.66122) | 0.78469 (0.56173,1.20706) | 0.79509 (0.56839,1.20999) | -30.6 (-53.4,-1.8) |
| Tuvalu | 0.05722 (0.03223,0.09518) | 0.62264 (0.34715,1.06332) | 0.03148 (0.01584,0.05664) | 0.26885 (0.13884,0.47879) | -56.8 (-72.4,-33.4) |
| Vanuatu | 0.77855 (0.46663,1.46273) | 0.59736 (0.3619,1.16069) | 1.0655 (0.61666,1.85247) | 0.37775 (0.2199,0.65367) | -36.8 (-58.7,-3.3) |
| Southeast Asia | 2439.74228 (1451.84963,3221.62755) | 0.6213 (0.34383,0.85137) | 2528.97775 (1303.71848,3216.25863) | 0.34949 (0.18389,0.44066) | -43.7 (-58.3,-20.3) |
| Cambodia | 52.0047 (37.5805,80.89754) | 0.59794 (0.43337,0.94206) | 58.0723 (39.16873,91.69656) | 0.36586 (0.25117,0.5679) | -38.8 (-55.7,-13.3) |
| Indonesia | 574.06689 (255.04673,787.93131) | 0.33404 (0.15374,0.47521) | 730.95301 (298.55428,1041.15322) | 0.25109 (0.10477,0.35524) | -24.8 (-48.8,10) |
| Lao People's Democratic Republic | 23.68099 (15.46743,40.6054) | 0.66283 (0.44825,1.10503) | 20.92503 (13.81672,33.5998) | 0.3084 (0.20548,0.4833) | -53.5 (-69.1,-28.9) |
| Malaysia | 137.76961 (109.45829,182.43387) | 0.94054 (0.7434,1.24103) | 152.47242 (120.2046,213.99699) | 0.47201 (0.37387,0.66271) | -49.8 (-66.6,-31.7) |
| Maldives | 0.56553 (0.35839,0.86119) | 0.32811 (0.21538,0.5062) | 0.51391 (0.35921,0.84924) | 0.09151 (0.06552,0.13817) | -72.1 (-82.3,-54.5) |
| Mauritius | 0.4359 (0.40989,0.46341) | 0.04363 (0.04121,0.04616) | 0.47165 (0.42227,0.52171) | 0.03013 (0.02698,0.03338) | -30.9 (-39.2,-22.3) |
| Myanmar | 738.69225 (381.49206,1020.75015) | 2.17906 (0.98404,3.05263) | 533.24123 (232.99738,753.56161) | 0.96914 (0.42325,1.36308) | -55.5 (-69.3,-31.8) |
| Philippines | 103.70793 (82.76751,128.55198) | 0.18486 (0.1515,0.23558) | 126.94962 (104.30655,166.35094) | 0.1196 (0.09768,0.15578) | -35.3 (-46.7,-17) |
| Seychelles | 0.28423 (0.23038,0.46138) | 0.42854 (0.34661,0.68983) | 0.23044 (0.16992,0.35828) | 0.20337 (0.15033,0.31802) | -52.5 (-65.1,-38.9) |
| Sri Lanka | 66.81691 (52.86612,87.20553) | 0.41643 (0.33219,0.54885) | 31.62539 (21.04057,48.16745) | 0.13313 (0.08966,0.20105) | -68 (-79.1,-53) |
| Thailand | 153.80254 (109.56659,238.34518) | 0.29736 (0.21674,0.44318) | 146.92002 (102.1091,192.37817) | 0.19082 (0.12957,0.24944) | -35.8 (-61.1,-4.8) |
| Timor-Leste | 2.99306 (1.90066,5.23396) | 0.42291 (0.27972,0.80656) | 3.44717 (2.2208,5.53142) | 0.28858 (0.18844,0.47117) | -31.8 (-52.8,2.3) |
| Viet Nam | 581.39243 (276.75375,942.93986) | 1.14121 (0.48367,1.89476) | 719.62816 (267.41418,1041.65297) | 0.65839 (0.25669,0.94376) | -42.3 (-68.4,-1.5) |
| Central Sub-Saharan Africa | 1114.37233 (775.04368,1477.97681) | 2.35763 (1.6476,3.18415) | 1348.65229 (809.43908,2217.58598) | 1.53153 (0.94079,2.26074) | -35 (-52.2,-9.2) |
| Angola | 248.23331 (162.26815,343.6447) | 2.73483 (1.89003,3.53142) | 283.2721 (167.39273,480.8682) | 1.32788 (0.83029,1.85549) | -51.4 (-67.6,-22.5) |
| Central African Republic | 79.0278 (55.03488,111.85641) | 3.48537 (2.53569,4.84116) | 102.87763 (63.39478,164.89428) | 2.62346 (1.62116,3.84596) | -24.7 (-43.7,7.3) |
| Congo | 36.14751 (25.58054,50.11155) | 2.08301 (1.495,2.6794) | 40.79773 (24.70943,63.14004) | 1.13484 (0.69863,1.68723) | -45.5 (-60.9,-18.6) |
| Democratic Republic of the Congo | 728.87555 (487.30363,1048.91196) | 2.21194 (1.51653,3.20745) | 902.79637 (523.03218,1527.37232) | 1.58409 (0.94705,2.48034) | -28.4 (-49.8,0.8) |
| Equatorial Guinea | 9.6308 (6.5851,13.43136) | 2.73781 (1.93473,3.58466) | 6.66229 (3.07606,12.23264) | 0.7935 (0.39532,1.41467) | -71 (-83,-47.3) |
| Gabon | 12.45737 (8.29061,16.83159) | 1.64949 (1.10955,2.18284) | 12.24617 (7.03268,19.91751) | 0.9589 (0.55293,1.51427) | -41.9 (-61,-16.1) |
| Eastern Sub-Saharan Africa | 4673.59374 (3319.99049,5754.31663) | 2.87935 (2.16545,3.66049) | 4803.11624 (3020.92864,6767.30164) | 1.6864 (1.13956,2.15055) | -41.4 (-53.4,-21.4) |
| Burundi | 141.92287 (88.27583,196.76459) | 3.02272 (1.97227,4.19155) | 173.73918 (84.49339,321.18807) | 2.09757 (1.13911,3.61811) | -30.6 (-54.2,9) |
| Comoros | 8.75728 (5.21119,12.84547) | 2.23873 (1.3941,3.2063) | 8.92236 (5.66079,12.98445) | 1.53584 (0.99017,2.16324) | -31.4 (-51.6,-0.6) |
| Djibouti | 5.3085 (3.32298,8.17895) | 1.81243 (1.16725,2.64544) | 12.71053 (7.21604,19.96811) | 1.51968 (0.89321,2.23337) | -16.2 (-42.6,23.9) |
| Eritrea | 76.53716 (54.27268,102.83196) | 3.10894 (2.25844,3.93804) | 101.09862 (59.52419,151.16278) | 2.28936 (1.40304,3.12066) | -26.4 (-45.3,4.7) |
| Ethiopia | 1726.48526 (1133.38341,2373.11701) | 4.26496 (2.93587,5.50479) | 1232.98299 (876.87866,1642.58219) | 1.73581 (1.25259,2.1385) | -59.3 (-70.2,-35.1) |
| Kenya | 360.60374 (208.94341,672.13983) | 2.01405 (1.20677,4.09252) | 515.24516 (286.17871,844.89521) | 1.66987 (0.96418,2.59416) | -17.1 (-43.6,15.5) |
| Madagascar | 218.33008 (145.09919,288.57027) | 2.10915 (1.50619,2.67308) | 286.31575 (172.70156,404.80519) | 1.48921 (0.92803,2.0344) | -29.4 (-50.4,-2.7) |
| Malawi | 239.58877 (156.37038,324.28405) | 2.42806 (1.70698,3.20572) | 183.96095 (110.88248,299.29413) | 1.51226 (0.97711,2.21301) | -37.7 (-54.2,-8.1) |
| Mozambique | 354.519 (231.54467,487.48079) | 2.64118 (1.91911,3.41322) | 384.84114 (225.72722,679.20104) | 1.86516 (1.17867,2.65028) | -29.4 (-49.2,-1.1) |
| Rwanda | 157.76918 (108.04453,214.47572) | 2.66353 (1.95758,3.43431) | 111.61738 (69.38036,186.9939) | 1.22233 (0.79242,1.79862) | -54.1 (-67.5,-29.7) |
| Somalia | 222.58313 (142.08861,337.79035) | 3.89812 (2.63949,6.55547) | 456.64976 (259.82779,879.21226) | 3.5206 (2.01848,6.73498) | -9.7 (-36.3,25.2) |
| South Sudan | 136.37489 (77.5579,207.49962) | 2.62917 (1.50909,3.8701) | 205.52238 (114.30138,315.71624) | 2.69036 (1.58542,4.00922) | 2.3 (-30.3,52) |
| Uganda | 305.00657 (194.15052,447.75377) | 2.08784 (1.4306,3.15819) | 416.6115 (216.70033,691.44162) | 1.44 (0.84022,2.0018) | -31 (-53.6,6.1) |
| United Republic of Tanzania | 544.29564 (354.22589,749.33492) | 2.17018 (1.54807,2.8324) | 534.71595 (314.29745,958.31124) | 1.18827 (0.76166,1.78727) | -45.2 (-60.2,-17.8) |
| Zambia | 172.1716 (119.03038,233.21553) | 2.48234 (1.79222,3.29478) | 174.00105 (103.90524,263.45613) | 1.43829 (0.87183,1.96515) | -42.1 (-60.8,-17.1) |
| Southern Sub-Saharan Africa | 853.85228 (647.05073,1008.41016) | 1.76024 (1.34067,2.09805) | 1113.90739 (849.20281,1361.69395) | 1.42487 (1.09188,1.73627) | -19.1 (-37.2,1.3) |
| Botswana | 20.67583 (13.08044,34.9143) | 1.90821 (1.20337,3.22024) | 24.02209 (15.57482,39.5058) | 1.05068 (0.68719,1.69885) | -44.9 (-65.2,-16.7) |
| Eswatini | 13.38629 (9.32465,20.98001) | 2.01786 (1.39835,3.10749) | 19.70573 (12.55024,31.71827) | 1.89322 (1.22884,3.06195) | -6.2 (-44.3,49.6) |
| Lesotho | 22.34039 (14.02911,36.4351) | 1.68782 (1.05818,2.8219) | 39.51709 (27.15176,55.84308) | 2.33825 (1.61787,3.35188) | 38.5 (-24.4,126.6) |
| Namibia | 18.56781 (12.80356,27.07797) | 1.59374 (1.11673,2.35393) | 28.47957 (18.56625,43.04481) | 1.29844 (0.86257,1.91898) | -18.5 (-47.4,20.8) |
| South Africa | 575.71725 (437.05138,711.14543) | 1.6142 (1.26357,1.95025) | 600.50861 (479.39888,721.80697) | 1.05603 (0.83656,1.27098) | -34.6 (-43.9,-19.8) |
| Zimbabwe | 203.1647 (115.2323,271.14791) | 2.42083 (1.49604,3.14629) | 401.67432 (257.91518,560.22216) | 2.99631 (1.91324,4.12836) | 23.8 (-31.8,92.1) |
| Western Sub-Saharan Africa | 2842.5058 (2091.9052,3920.91488) | 1.6162 (1.20282,2.1534) | 4165.57696 (2622.84995,5735.69449) | 1.09441 (0.64542,1.48402) | -32.3 (-55,-6.5) |
| Benin | 62.08517 (40.3637,91.66604) | 1.21998 (0.87223,1.76992) | 120.44122 (52.2357,210.5979) | 1.03348 (0.46538,1.70901) | -15.3 (-56.2,34.8) |
| Burkina Faso | 248.89311 (168.49477,373.42591) | 2.45787 (1.82219,3.32863) | 330.63849 (195.08093,648.99218) | 1.61335 (0.99394,2.57904) | -34.4 (-52.9,-4.2) |
| Cabo Verde | 0.09465 (0.04049,0.38997) | 0.03033 (0.01339,0.12599) | 1.53753 (0.30873,2.24694) | 0.30437 (0.06322,0.44172) | 903.7 (-31.9,2750.4) |
| Cameroon | 96.60811 (64.25503,137.63556) | 1.01596 (0.71425,1.41895) | 218.84877 (93.95337,348.26321) | 0.91276 (0.40278,1.39796) | -10.2 (-52.7,37.5) |
| Chad | 79.42615 (49.77529,121.56508) | 1.2579 (0.86637,2.0486) | 233.96129 (121.94634,363.1697) | 1.56003 (0.79615,2.53337) | 24 (-27.7,91.3) |
| C么te d'Ivoire | 102.30342 (66.8085,158.08088) | 1.024 (0.70219,1.50649) | 204.41212 (89.21818,326.53675) | 0.96328 (0.43499,1.46309) | -5.9 (-49,47.4) |
| Gambia | 10.76092 (7.4,15.61861) | 1.38655 (1.00342,2.0322) | 18.31707 (11.07793,29.45217) | 1.16173 (0.69336,1.60928) | -16.2 (-45.5,22.7) |
| Ghana | 186.79352 (127.05012,266.55719) | 1.40818 (0.98215,2.02451) | 235.26536 (135.48168,383.90673) | 0.90145 (0.56614,1.34177) | -36 (-54.4,-11.2) |
| Guinea | 87.85929 (57.63958,130.63399) | 1.25855 (0.89266,1.97344) | 134.6719 (60.97818,225.75307) | 1.22407 (0.57365,2.0639) | -2.7 (-50.4,66.1) |
| Guinea-Bissau | 16.53951 (11.30928,23.67052) | 1.85616 (1.36562,2.50258) | 20.82573 (10.58465,32.47723) | 1.49316 (0.79115,2.20054) | -19.6 (-51.7,24.7) |
| Liberia | 33.31125 (20.83519,47.95956) | 1.22485 (0.8265,1.72101) | 41.68289 (17.36055,85.44926) | 1.03992 (0.44397,2.05384) | -15.1 (-57.7,64.2) |
| Mali | 138.40485 (85.14448,206.40153) | 1.53282 (1.07276,2.45015) | 244.42988 (114.43337,416.24899) | 1.27594 (0.60465,2.2416) | -16.8 (-53.1,29.2) |
| Mauritania | 15.00464 (10.57141,21.82391) | 0.84986 (0.60861,1.17079) | 20.93928 (10.3173,34.73826) | 0.64676 (0.30821,0.9732) | -23.9 (-59,14.9) |
| Niger | 149.39593 (79.81195,235.29032) | 1.65048 (1.12406,2.74801) | 276.96781 (116.6021,607.2551) | 1.34173 (0.59017,2.89382) | -18.7 (-59.6,44.9) |
| Nigeria | 1437.24537 (931.00453,2305.15416) | 1.8542 (1.32639,2.45189) | 1801.78713 (1292.25618,2436.38184) | 1.04615 (0.70342,1.4213) | -43.6 (-60.7,-13.8) |
| Sao Tome and Principe | 0.94612 (0.52102,1.50672) | 0.62754 (0.34284,0.98097) | 0.38265 (0.19427,0.79774) | 0.2167 (0.11642,0.39098) | -65.5 (-80.1,-42.4) |
| Senegal | 85.57665 (56.79215,128.92224) | 1.14714 (0.8059,1.67833) | 114.52786 (56.22286,210.18403) | 0.97116 (0.47995,1.64839) | -15.3 (-51.9,32.5) |
| Sierra Leone | 58.53321 (37.20436,84.67405) | 1.25265 (0.88682,1.74384) | 83.22555 (37.06569,143.4521) | 1.11958 (0.50787,1.92317) | -10.6 (-52,45.6) |
| Togo | 32.63013 (22.84662,44.55798) | 1.01782 (0.76511,1.41948) | 62.66583 (28.1359,105.48509) | 1.01608 (0.46157,1.62482) | -0.2 (-46.7,50.3) |

| **Table S3: DALY due to non-CO poisoning in 1990 and 2021 and the percentage change in the age-standardized DALY rate per 100,000, by location** | | | | | |
| --- | --- | --- | --- | --- | --- |
|  | DALYs_Number_1990.95._UI. | ASRs_1990_per_100000.95._UI. | DALYs_Number_2021.95._UI. | ASRs_2021_per_100000.95._UI. | Percentage_change_in_the_ASRs_from_1990_to_2021_per_100000 |
| Global | 2124840 (1796815,2786509) | 38.4 (32.6,49.9) | 1652096 (1139789,2057575) | 21.7 (14.9,27.5) | -27.4 (-81,39) |
| High-income Asia Pacific | 49374 (40043,60637) | 26.7 (21.6,33.1) | 23331 (16709,31041) | 10.9 (7.5,14.8) | -20.7 (-36.9,2.5) |
| Oceania | 2676 (1626,4649) | 36.3 (22,67.1) | 18087 (12200,24886) | 26 (17.5,35.7) | -75.2 (-80.4,-69.8) |
| Eastern Europe | 35395 (27023,46142) | 15.4 (11.8,20.1) | 3637 (2496,5062) | 11.3 (7.7,16) | -68.4 (-77.7,-50.5) |
| Central Europe | 75113 (64340,88119) | 59.4 (51.1,69.5) | 14261 (11599,18825) | 21.4 (17.4,28.2) | -59.1 (-65.7,-51.6) |
| East Asia | 329902 (202899,933556) | 27.5 (16.9,77.3) | 87780 (50337,122186) | 14 (8,19.5) | -53.3 (-58.2,-48.7) |
| Central Latin America | 93586 (84812,104765) | 54.7 (49.3,61.5) | 294562 (182481,445139) | 71.4 (46.2,99.6) | -67.3 (-75.1,-53.1) |
| Southeast Asia | 143347 (93755,179919) | 32.2 (20.5,41.2) | 131483 (75355,163743) | 17.9 (10.3,22.2) | -47.8 (-62.2,-22.4) |
| Australasia | 3677 (2842,4716) | 17.8 (13.8,22.9) | 3526 (2312,6796) | 23.3 (15.2,45.7) | -48.4 (-55.3,-42.7) |
| Tropical Latin America | 19495 (17260,21946) | 21.7 (18,26.6) | 279223 (180797,383516) | 51.4 (33.3,70.1) | -66.3 (-69.9,-62.4) |
| Central Asia | 15223 (12659,18490) | 12.6 (11.2,14.2) | 77834 (44733,144212) | 61.9 (38.1,99.7) | -58.6 (-65.1,-53) |
| High-income North America | 70397 (56680,87331) | 24.3 (19.5,30.3) | 64975 (48472,80055) | 79.3 (59.1,97.7) | -36 (-54.2,-5.8) |
| Southern Latin America | 29810 (24742,36348) | 60.2 (50,73.4) | 18188 (12520,25452) | 14.7 (10,20.8) | -62.8 (-66.8,-59) |
| Eastern Sub-Saharan Africa | 326539 (227859,408385) | 136.6 (100.3,167.5) | 8640 (6483,11160) | 9 (6.7,11.6) | -44.3 (-56.9,-25.8) |
| South Asia | 252638 (135508,327699) | 20 (11.2,25.3) | 52301 (43872,63442) | 20.3 (17,24.7) | -56.8 (-64.9,-49.7) |
| North Africa and Middle East | 197597 (108969,264570) | 47.5 (26.6,64.4) | 45757 (34406,59289) | 11.7 (8.8,15.2) | -36.5 (-59.8,-5.3) |
| Western Europe | 89595 (70488,112333) | 22.4 (17.6,28.3) | 15859 (11870,20554) | 7.2 (5.4,9.3) | -51.9 (-55.3,-49.1) |
| Andean Latin America | 34825 (29526,39530) | 83.8 (71.7,94) | 9687 (7327,14011) | 21.6 (16,32.1) | -36.4 (-44.2,-28.7) |
| Western Sub-Saharan Africa | 197447 (143119,289890) | 81.1 (61.4,108.5) | 9913 (7922,12048) | 4.3 (3.4,5.2) | -43.5 (-61.8,-24.3) |
| Southern Sub-Saharan Africa | 55224 (41596,65246) | 100 (76.9,118.1) | 53306 (36427,72585) | 11.6 (7.9,16) | -74.4 (-79.5,-65.6) |
| Caribbean | 25760 (19246,33688) | 68.5 (52.4,87.6) | 320520 (143253,414845) | 20 (8.9,25.5) | -44.9 (-61.5,-12.4) |
| Central Sub-Saharan Africa | 77220 (51608,102817) | 112.3 (80.8,147.7) | 119226 (78842,157547) | 6.5 (4.3,8.6) | -70.5 (-77.3,-60.4) |
| Cuba | 2870 (2546,3274) | 26.8 (23.8,30.4) | 635 (424,885) | 10.3 (6.7,14.4) | -35.7 (-58,-4.1) |
| Ukraine | 5299 (3993,6855) | 9.9 (7.5,12.7) | 2430 (1620,3391) | 43.7 (29,61) | -62.2 (-67.1,-57.8) |
| Argentina | 22428 (18442,27509) | 68 (55.8,83.4) | 41117 (30989,53413) | 11.7 (8.8,15.2) | -45.1 (-55.5,-36.1) |
| Bolivia (Plurinational State of) | 7454 (5647,9907) | 100.9 (78,131.9) | 14328 (6531,24870) | 30.2 (13.9,52.1) | -29.1 (-42.8,-16.5) |
| Samoa | 42 (29,66) | 11.3 (7.7,16.2) | 976 (786,1211) | 19.6 (15.7,24.3) | -48.3 (-60.2,-30.6) |
| Tajikistan | 580 (384,918) | 23.3 (16.1,37) | 5280 (3406,9350) | 37.8 (25,66.2) | -46.3 (-57.7,-36.4) |
| Venezuela (Bolivarian Republic of) | 8073 (6876,9636) | 42.9 (36.6,51.3) | 90 (66,123) | 1.9 (1.4,2.6) | -51.1 (-59.8,-43.5) |
| Luxembourg | 80 (64,100) | 20 (15.8,25.1) | 22 (11,58) | 3 (1.4,8) | -38.4 (-59.4,11.3) |
| Botswana | 1300 (833,2124) | 99.8 (63.9,170.2) | 12 (9,18) | 10.9 (8.3,15.7) | -52.7 (-63,-39.7) |
| Myanmar | 41193 (24908,55064) | 108.2 (59.9,145.4) | 316 (243,391) | 9.9 (7.7,12.4) | -57 (-65.8,-45.6) |
| Sierra Leone | 4430 (2582,6628) | 74.2 (49.5,104.4) | 1118 (770,1563) | 15.4 (10.5,21.6) | -67.9 (-77.9,-56.4) |
| C么te d'Ivoire | 7430 (4717,11480) | 51.4 (35.3,76.7) | 696 (463,983) | 11.2 (7.5,16.2) | -68.7 (-80.8,-48.7) |
| American Samoa | 7 (5,10) | 15.1 (10.1,19.6) | 16863 (8260,27486) | 60.7 (30,101.2) | -64.4 (-73.7,-54.8) |
| Puerto Rico | 3384 (3136,3659) | 94.5 (87.4,102.1) | 310069 (132774,405384) | 20 (8.6,25.8) | -31 (-61.7,20.5) |
| Dominican Republic | 4198 (2369,5487) | 49.7 (29.8,63.7) | 7402 (5342,9479) | 10.8 (7.5,13.9) | -59.1 (-73.4,-27.6) |
| Peru | 16059 (11848,19618) | 67.6 (51,81) | 69 (42,116) | 21.4 (13.1,36.1) | -43.6 (-60.3,-16.6) |
| Poland | 24014 (21318,27070) | 61.6 (54.7,69.4) | 61 (44,83) | 12.9 (9.2,17.4) | -37.6 (-46.3,-29) |
| Austria | 1764 (1381,2218) | 21.9 (17.1,27.6) | 3048 (2796,3331) | 11.2 (10.3,12.1) | 2.4 (-34.6,52.8) |
| Uruguay | 1754 (1494,2060) | 56.5 (48.2,66.2) | 884 (597,1263) | 15.3 (10.2,22.1) | -39.9 (-60.3,-8.3) |
| Uzbekistan | 2797 (2089,3928) | 13.2 (9.8,18.4) | 31424 (13347,44862) | 28.6 (12.6,40.4) | -59.4 (-66.8,-51.8) |
| Cyprus | 133 (95,176) | 17 (12.1,22.5) | 2656 (1119,5418) | 48.8 (21.2,98) | -87.8 (-92.5,-75.6) |
| Vanuatu | 53 (33,96) | 33.6 (21,61.5) | 1410 (977,2234) | 38.2 (26.3,58.3) | -21.6 (-60.6,38) |
| Libya | 1332 (599,2022) | 28.3 (13.4,41.9) | 7 (5,10) | 8.4 (5.8,12.1) | -74.5 (-85.1,-50.7) |
| Sri Lanka | 4151 (3363,5234) | 23.6 (19.2,29.9) | 7568 (5152,10540) | 10.8 (7.2,15.1) | -47.2 (-64.7,-9.2) |
| Brazil | 18496 (16343,20850) | 12.3 (10.9,13.9) | 5201 (3589,7138) | 10.7 (7.2,15.1) | -38.6 (-59.6,-2.8) |
| China | 300830 (175770,894198) | 26 (15.2,76.8) | 7071 (5066,10092) | 11.7 (8.2,16.7) | -27.8 (-47.8,3.1) |
| San Marino | 11 (8,13) | 43.6 (34.3,51.7) | 2937 (2030,4093) | 11 (7.6,15.3) | -49.5 (-65.7,-20) |
| Guinea | 6573 (4051,10154) | 75 (51.7,109.8) | 49 (37,67) | 7.3 (4.8,12) | -56 (-70.7,-31.2) |
| Cook Islands | 2 (1,3) | 10.1 (7.4,13.3) | 3118 (1890,5313) | 14.7 (6.5,20.4) | -49 (-57.1,-40.9) |
| Thailand | 8886 (6487,13404) | 15.4 (11.4,22.7) | 43070 (19047,60058) | 43 (24.3,65.2) | -71.7 (-83.1,-45.5) |
| Canada | 7356 (6248,8595) | 25.9 (21.9,30.3) | 56 (31,86) | 7.6 (5.6,10) | -67.7 (-81.2,-37.9) |
| Tonga | 65 (47,93) | 61.5 (45.6,86.8) | 14 (10,18) | 14.2 (10.7,18.4) | -89.2 (-91.7,-86.1) |
| South Africa | 37232 (27498,46875) | 95 (72.1,117.3) | 541 (409,696) | 20.1 (12.9,33.2) | -72.7 (-84.3,-48.6) |
| Democratic Republic of the Congo | 50760 (32658,74051) | 105 (72.9,152) | 2 (1,4) | 8.5 (6.2,11.2) | -50.4 (-57.8,-43.7) |
| Brunei Darussalam | 56 (42,72) | 22.9 (17.3,29.1) | 862 (629,1123) | 64.7 (29.4,138.9) | -71 (-78.4,-62.4) |
| Mauritius | 47 (39,57) | 4.3 (3.6,5.1) | 19832 (8531,42707) | 11.4 (7.7,15.9) | -56.7 (-67.4,-43.4) |
| Greenland | 14 (9,18) | 24.3 (15.4,31.1) | 42 (29,58) | 3.2 (2.5,4.5) | -48 (-63.4,-28) |
| Serbia | 2751 (2012,3633) | 28.2 (20.6,37.2) | 2040 (1347,2906) | 18.2 (11.8,26.3) | -32.9 (-41.3,-26) |
| Bangladesh | 55120 (4794,96934) | 32.8 (3.7,55.5) | 456 (285,710) | 65.5 (41.9,99.9) | -79.3 (-82.9,-74.6) |
| Slovakia | 1593 (1127,2056) | 29.5 (20.7,38) | 32 (25,40) | 10.2 (8.1,13) | -73.1 (-79.7,-65.8) |
| Netherlands | 2009 (1407,2712) | 13 (9.1,17.5) | 9207 (8069,10440) | 57 (49.7,64.6) | -37.2 (-45.7,-28.4) |
| Palestine | 347 (247,484) | 14 (10.3,19.8) | 29527 (20141,49343) | 98.6 (68.5,157.2) | -59.5 (-74.2,-39.1) |
| Colombia | 13866 (12159,15946) | 41.7 (36.4,48.1) | 13789 (7599,21933) | 124.6 (70.8,190.2) | -51.4 (-54.6,-48.9) |
| Tokelau | 0 (0,1) | 21 (11.6,35.9) | 23674 (13230,50698) | 80.7 (50,149.5) | -55.8 (-63.2,-49.5) |
| Guyana | 639 (552,723) | 75.9 (66.2,85.2) | 7403 (3681,17365) | 26.1 (12.6,60.2) | -51.6 (-66.6,-34.8) |
| Bosnia and Herzegovina | 6706 (4448,9226) | 147.1 (96.5,202.5) | 1182 (756,1879) | 100.8 (65.5,161) | -16.5 (-54.5,30.2) |
| Zimbabwe | 13283 (7471,18345) | 123.5 (73.9,162.1) | 210 (144,325) | 15.2 (10.4,23.6) | -63.9 (-75.4,-48.3) |
| Cambodia | 3439 (2427,5270) | 33.1 (24.7,50.5) | 513 (370,712) | 18.8 (13.6,26) | -22.1 (-59.2,48.3) |
| Viet Nam | 29649 (16605,45862) | 51.8 (25.8,81.9) | 35266 (19750,69188) | 56.2 (34.3,99) | -41.5 (-54.7,-29.8) |
| Liberia | 2529 (1478,3806) | 71.8 (47.8,98.8) | 1284 (655,2110) | 65.8 (35.3,100.2) | -63.8 (-69.3,-59.3) |
| Taiwan (Province of China) | 23853 (23067,24591) | 115.1 (111.3,118.7) | 123 (95,157) | 8.7 (6.7,11) | -17.1 (-44.1,24.9) |
| Republic of Korea | 20989 (16349,27546) | 49.5 (38.5,64) | 6 (4,8) | 3.7 (2.8,5.3) | -61.3 (-68.7,-48.8) |
| United States Virgin Islands | 20 (13,25) | 18.6 (11.9,23.8) | 2 (1,3) | 15.5 (8.5,27.2) | -46.1 (-63.1,-19.1) |
| Gabon | 669 (430,974) | 68.4 (46.5,93.1) | 1310 (1033,1666) | 19.6 (15.5,24.8) | -82.8 (-86.4,-78.7) |
| India | 121539 (47676,161103) | 13.2 (5.4,17.6) | 1544 (1127,2033) | 13.4 (9.7,18) | -68.3 (-72,-64.8) |
| Qatar | 35 (23,53) | 8 (5.4,11.9) | 15503 (10899,20754) | 10.6 (7.3,14.5) | -76.1 (-83.3,-65.6) |
| Spain | 8521 (6778,10676) | 21.3 (17,26.6) | 371 (242,541) | 16.9 (10.8,24.9) | -46.6 (-59.5,-34.2) |
| Guatemala | 14045 (13143,15090) | 150.9 (142.1,160.8) | 544 (369,751) | 9.3 (6.2,13.1) | -55.1 (-67.6,-32.5) |
| Saint Lucia | 78 (70,87) | 57.1 (51.9,63.1) | 184 (131,251) | 9.1 (6.4,12.5) | -65.3 (-75.1,-53.9) |
| Croatia | 1490 (1199,1851) | 29.3 (23.6,36.4) | 274 (208,360) | 9.5 (7.3,12.4) | -49.9 (-67.2,-16.1) |
| Rwanda | 10872 (7241,15722) | 126.6 (89.1,166.8) | 2334 (1671,3445) | 5.6 (4,8.3) | 11.3 (-36.7,87.2) |
| Benin | 4689 (2922,6928) | 68.1 (46.8,96.3) | 4395 (2213,6510) | 9.9 (5,14.5) | -34.7 (-56.6,9.7) |
| France | 10844 (8299,13752) | 18.2 (13.8,23.2) | 2997 (2350,4026) | 46.2 (36.4,61.7) | -40.5 (-62.9,-2.5) |
| Mauritania | 998 (688,1518) | 42.1 (30.5,59.8) | 4 (3,6) | 5.9 (3.8,8.4) | -78.7 (-88,-50) |
| Saudi Arabia | 4804 (3373,7268) | 26.4 (18.7,40.7) | 910 (484,1348) | 7.8 (4.1,11.6) | -37.8 (-58.8,9.9) |
| Nepal | 65534 (40396,103327) | 278.5 (191.3,413.1) | 1972 (1476,2598) | 18 (13.3,23.7) | -49.6 (-57.9,-41.5) |
| Burundi | 9602 (5893,13660) | 141.4 (89.3,195.8) | 7031 (5377,13290) | 8.1 (6.2,15.7) | -67.1 (-78.2,-47.5) |
| Bahamas | 128 (113,144) | 49.4 (44.1,55.4) | 74052 (51271,107446) | 71.3 (51.2,93.9) | -74 (-83.3,-55.7) |
| Greece | 2083 (1685,2563) | 19.3 (15.6,23.8) | 9191 (6512,12324) | 12.9 (9.1,17.5) | -18.1 (-26.9,-8.7) |
| Bahrain | 42 (33,58) | 14.9 (11.5,19.4) | 19 (12,31) | 17.9 (11.4,29.8) | 221.2 (-42.6,528) |
| Kyrgyzstan | 655 (510,846) | 18.8 (14.1,29.5) | 2251 (1666,3843) | 2.8 (2,4.9) | -63.1 (-72.3,-44.1) |
| Azerbaijan | 1397 (1045,2218) | 18.7 (13.8,25.2) | 10 (8,13) | 8.8 (6.9,11.4) | -32.4 (-41.8,-24.1) |
| Fiji | 151 (111,208) | 86.1 (55.2,111.5) | 10602 (6356,17635) | 59.5 (36.4,88.5) | -86.8 (-90,-82.8) |
| Monaco | 27 (17,35) | 170.6 (113.2,265.5) | 7630 (6097,10435) | 22.5 (17.9,30.7) | -58.3 (-72.3,-19.9) |
| Somalia | 15399 (9388,23220) | 106.9 (64.5,155) | 5959 (3456,9778) | 96.4 (58.7,144.1) | -75.6 (-85.6,-55.9) |
| Comoros | 600 (346,905) | 8.3 (6.5,11.5) | 16785 (8695,25835) | 76.4 (40.8,117.7) | -10.7 (-51.3,41.1) |
| Estonia | 394 (318,503) | 25.2 (20.4,31.8) | 2 (2,4) | 5 (3.7,8) | -76.2 (-83.2,-69.5) |
| Burkina Faso | 18415 (11901,28756) | 130.9 (92.1,188.1) | 520 (379,683) | 7.7 (5.6,10.1) | -31.6 (-53.1,-1.7) |
| Switzerland | 765 (533,1061) | 10.7 (7.4,14.9) | 26 (14,58) | 12.2 (6.9,25.1) | -14.5 (-53.9,40.2) |
| Mexico | 42077 (37736,47523) | 47.3 (42.2,53.7) | 78 (25,112) | 14.4 (4.6,20.5) | -58.8 (-72.3,-34) |
| Suriname | 272 (125,348) | 68.1 (32.1,86.4) | 1187 (826,1621) | 9.8 (6.8,13.5) | -58.7 (-70.2,-41.4) |
| United Republic of Tanzania | 39880 (25333,57441) | 112.3 (76.7,151.3) | 12861 (8689,17814) | 27.6 (18.7,38.2) | -67.2 (-76.8,-54) |
| Tunisia | 3289 (1447,5094) | 35 (15.5,54.9) | 361 (246,518) | 15.4 (10.3,22) | -3.7 (-35.7,53.7) |
| Ireland | 818 (663,997) | 22.7 (18.4,27.6) | 988 (668,1371) | 10.3 (6.9,14.3) | -55 (-59.9,-49.6) |
| Micronesia (Federated States of) | 39 (25,60) | 35.4 (23.1,53.7) | 8963 (5987,12687) | 9.9 (6.5,14.3) | -50.8 (-68.5,-20.4) |
| North Macedonia | 659 (468,849) | 33.2 (23.2,42.6) | 419 (312,546) | 10.9 (8.2,14.3) | -39.6 (-48.4,-31.7) |
| Belize | 368 (332,406) | 162 (148.1,177.2) | 3500 (2483,5435) | 29.5 (21,45.4) | -61.9 (-77.2,-33.7) |
| Republic of Moldova | 1436 (1116,1845) | 32.4 (25.2,41.4) | 5 (4,8) | 9 (6.1,14.5) | -57.1 (-70.2,-34.8) |
| Mongolia | 1218 (686,1983) | 54.5 (32.6,82.6) | 1467 (982,2005) | 12.2 (8.1,16.9) | -26 (-34.5,-16.6) |
| Eritrea | 5151 (3502,7331) | 139.5 (102,180.1) | 6832 (5459,8528) | 25.8 (20.6,32.2) | -68 (-75.5,-55.5) |
| Northern Mariana Islands | 5 (3,8) | 11.2 (6.2,16.1) | 6297 (3744,11033) | 115.4 (72.1,179.9) | -23 (-81.6,56.3) |
| Sao Tome and Principe | 76 (44,123) | 44.8 (26.3,69.8) | 7 (5,9) | 7.5 (5.4,9.8) | -50.7 (-71.1,-18.4) |
| Angola | 17725 (10967,25572) | 134.8 (91.3,181.1) | 1243 (858,1691) | 13.2 (9,18.1) | -53.5 (-59.8,-48.3) |
| Panama | 905 (779,1082) | 37.5 (32,44.9) | 11187 (8199,14509) | 10.4 (7.7,13.4) | -33.1 (-56.8,4.6) |
| United Arab Emirates | 190 (117,303) | 10.2 (6.2,16.5) | 811 (575,1117) | 22.9 (16.2,31.7) | -53.9 (-68.3,-23.3) |
| Iran (Islamic Republic of) | 46579 (30028,64916) | 66.9 (44.3,94.4) | 8475 (7053,10657) | 7.4 (6.2,9.3) | -66.3 (-73.9,-58.8) |
| Jordan | 1339 (902,2074) | 30.4 (20.7,48.3) | 11638 (6596,34442) | 4.7 (2.7,13.5) | -42.7 (-60.6,-14.5) |
| Cabo Verde | 15 (9,33) | 4.5 (2.8,9.2) | 1889 (1403,2479) | 9.9 (7.3,13) | 24 (16.7,29.7) |
| Malaysia | 7805 (6271,10299) | 46.4 (37.4,60.7) | 126 (99,162) | 2.8 (2.2,3.5) | -79.3 (-83.1,-74.9) |
| Israel | 986 (768,1253) | 20.1 (15.7,25.5) | 2287 (1582,3182) | 122 (83.9,171.5) | -38.7 (-56.7,-3.4) |
| Zambia | 12533 (8253,17910) | 120.8 (84,157) | 3964 (2842,5703) | 41 (30.2,58.5) | -38.8 (-47.3,-24.3) |
| Papua New Guinea | 1876 (1093,3460) | 39.9 (22.5,79.3) | 10254 (4052,19798) | 28.3 (10.7,54.8) | -39.6 (-62.2,-8.7) |
| Kenya | 24943 (14684,43723) | 90.9 (55.2,173.4) | 11 (6,19) | 18.5 (9.9,32.7) | -64.4 (-71.3,-53.8) |
| Montenegro | 148 (107,200) | 23.3 (16.8,31.5) | 1566 (1040,2263) | 15.3 (10.2,22.4) | -28.7 (-51.2,9.9) |
| Russian Federation | 23511 (17180,31224) | 15.3 (11.2,20.2) | 1915 (1410,2410) | 17.3 (12.7,21.8) | -36.1 (-57.9,-5.8) |
| Philippines | 7774 (6159,9491) | 12.1 (10,14.8) | 17872 (10564,27310) | 64.9 (40.2,90.7) | -90.3 (-91.1,-89.5) |
| Turkmenistan | 739 (545,924) | 19 (14.6,23.7) | 422 (308,557) | 8.2 (6,10.8) | -54 (-63.2,-45.2) |
| Solomon Islands | 156 (85,327) | 43.7 (23.4,90.3) | 3715 (1713,6696) | 45.8 (21.7,76.7) | 5.3 (-41.8,98.5) |
| Democratic People's Republic of Korea | 5219 (2414,16716) | 24.7 (11.6,79) | 191 (113,351) | 27.2 (16.3,48.8) | -86.1 (-89.7,-81.9) |
| Italy | 17633 (13937,22004) | 29.4 (23.1,36.9) | 69 (46,96) | 10.1 (6.7,14.2) | -83.9 (-87.7,-80.1) |
| Romania | 17334 (13910,21960) | 73.9 (59.5,93.7) | 6906 (5376,9546) | 18.8 (14.7,26) | -70.7 (-82.5,-59.8) |
| Andorra | 7 (5,10) | 11.8 (8,16.9) | 50814 (28591,97331) | 63 (37.1,104.1) | -40 (-48.2,-30.1) |
| Yemen | 17192 (5414,30395) | 87.6 (28.6,160.1) | 9212 (7335,11251) | 4.1 (3.2,5) | -30.9 (-54.5,11.6) |
| Chile | 5627 (4722,6819) | 42.2 (35.4,51.1) | 49 (32,70) | 10.4 (6.8,15) | -76.2 (-84.1,-27.8) |
| Kuwait | 242 (218,272) | 13.5 (12.1,15.2) | 5 (4,7) | 8.8 (6.9,11.4) | -82.9 (-86.3,-79.2) |
| Malta | 58 (43,77) | 15.4 (11.3,20.2) | 1074 (658,1885) | 48.4 (30.2,76.4) | -63.1 (-74.6,-36.1) |
| Belgium | 2714 (2039,3538) | 26.2 (19.5,34.3) | 1236 (833,1931) | 16.4 (11.1,25.6) | -67.9 (-76.9,-54.1) |
| Lebanon | 492 (364,642) | 16.1 (12.1,21) | 3266 (2267,4467) | 16.1 (11.2,22.3) | -51.5 (-56.4,-46.5) |
| Madagascar | 15249 (9784,21169) | 102.9 (71.6,131.8) | 33636 (26125,40046) | 58 (44.9,68.7) | -34.5 (-44.9,-25.4) |
| Dominica | 135 (73,168) | 184 (100.4,227.1) | 573 (387,796) | 11.1 (7.5,15.5) | -64.6 (-77.1,-41) |
| Central African Republic | 5241 (3489,7730) | 162.3 (116,227.2) | 14332 (6464,23620) | 43.6 (19.9,68.1) | -41.9 (-49.8,-34.5) |
| Indonesia | 38372 (18882,49234) | 20.4 (10.5,26.5) | 930 (452,1613) | 13.9 (6.9,24) | -19.6 (-60.6,46.9) |
| Ecuador | 11312 (10259,12327) | 107.1 (97.1,116.7) | 39 (31,48) | 9 (7.1,11.2) | -78.8 (-84,-72.9) |
| Gambia | 753 (502,1119) | 65.8 (46.9,94.3) | 729 (572,941) | 16.9 (13.2,21.7) | -55.5 (-67,-44) |
| Japan | 27765 (21604,35027) | 20.3 (15.6,25.8) | 896 (619,1557) | 7.3 (5.1,12.8) | -26.4 (-52.4,14) |
| Malawi | 18000 (11601,25611) | 127.8 (86.4,169.1) | 17680 (10259,34197) | 54.8 (33.7,86.9) | -74.8 (-79.7,-69.2) |
| Slovenia | 637 (507,811) | 30.9 (24.6,39.5) | 619 (416,1046) | 4.5 (3,7.6) | -38.9 (-52.7,-27.7) |
| Timor-Leste | 213 (132,356) | 25 (16.7,41.9) | 26916 (14240,46743) | 64.5 (36.4,107.6) | -74.7 (-83.2,-58.9) |
| Congo | 2205 (1518,3284) | 90.5 (64.3,123.4) | 9 (7,12) | 8.5 (6.6,11) | -17.6 (-46.3,24.5) |
| Morocco | 17349 (6711,28558) | 57.4 (22.5,95.7) | 1474 (980,2288) | 60.3 (39.8,93.6) | -6.3 (-38.1,31.3) |
| Ghana | 13117 (8708,19118) | 73.1 (51,102.5) | 0 (0,1) | 21.5 (9.8,43.7) | 39 (-19.7,128.2) |
| Denmark | 849 (640,1101) | 15.7 (11.8,20.6) | 4 (3,6) | 8 (6,11.3) | -69.2 (-76.9,-62.1) |
| Lesotho | 1366 (866,2181) | 87.8 (55,143.6) | 286 (218,375) | 3.2 (2.3,4.5) | -67 (-70.6,-63.4) |
| Norway | 1513 (1048,2064) | 35.2 (24.5,48) | 13312 (5952,21733) | 45.9 (21.5,71.2) | -49.9 (-58.2,-40.7) |
| United States of America | 63026 (50318,78594) | 24.1 (19.2,30.2) | 798 (547,1099) | 8 (5.5,10.9) | -55.4 (-66.1,-34.3) |
| Togo | 2422 (1633,3393) | 53.6 (39.8,71.9) | 26766 (14387,37093) | 46.4 (25.2,64) | -29 (-47.2,1.8) |
| Paraguay | 999 (747,1226) | 22.1 (17.2,26.8) | 41 (33,49) | 2.9 (2.3,3.5) | -39.3 (-57.6,-7.3) |
| Grenada | 109 (96,123) | 122.5 (109.5,136.4) | 5701 (2665,9670) | 57.8 (27.3,98.2) | -30.1 (-56.4,2.7) |
| Bermuda | 14 (11,17) | 22 (17.3,27.2) | 239 (159,335) | 6.7 (4.4,9.4) | -32.1 (-68.8,54.5) |
| Albania | 1549 (1119,1897) | 45.6 (33.7,55.6) | 2715 (1953,3604) | 5.7 (4.1,7.7) | -63.3 (-72.1,-54.3) |
| Afghanistan | 19442 (7508,32457) | 156.3 (59.6,265.9) | 4138 (2828,5677) | 10 (6.8,13.8) | -42.7 (-55.2,-22.5) |
| Namibia | 1160 (816,1724) | 82.6 (57.7,120) | 26 (17,42) | 60.1 (35,93.6) | -94.5 (-95.6,-93.3) |
| Mozambique | 26026 (16439,36870) | 140.4 (94.6,189.2) | 668 (384,1092) | 9.8 (7.8,13.9) | -72.1 (-79.6,-59) |
| Lao People's Democratic Republic | 1555 (1000,2571) | 37.2 (25.4,62) | 701 (554,991) | 12.1 (8.1,19.8) | -53 (-58.4,-48.8) |
| Saint Kitts and Nevis | 22 (20,25) | 55 (50.4,60.4) | 2646 (1908,3479) | 7.7 (5.6,10.2) | -70.8 (-80.2,-55.6) |
| Kiribati | 59 (37,81) | 68.4 (42,93.7) | 120154 (86331,163109) | 47.2 (34.3,63.4) | -66.9 (-72.1,-61.7) |
| Bhutan | 57 (33,126) | 7.8 (4.6,17.2) | 1 (1,1) | 3.6 (2.8,4.9) | -48.1 (-65.6,-22.6) |
| Costa Rica | 878 (739,1064) | 30.1 (25.4,36.3) | 1772 (1280,2496) | 7.7 (5.6,10.8) | -40.8 (-60.2,-22.8) |
| Armenia | 766 (619,942) | 22.4 (18,27.6) | 4635 (3416,5995) | 11.4 (8.4,14.8) | -50.8 (-68.3,-12) |
| Belarus | 3069 (2476,3763) | 29.5 (24.1,36.4) | 149 (102,208) | 10.4 (7,14.6) | -80.2 (-83.4,-76.7) |
| Algeria | 12735 (5403,19345) | 42.3 (18.4,64.6) | 8 (6,10) | 21.8 (16,29.1) | -43.1 (-53.8,-30.5) |
| Guinea-Bissau | 1169 (736,1759) | 95.4 (67.8,131.4) | 1547 (1044,2221) | 16.1 (10.7,23.4) | -43 (-52.1,-34.1) |
| Guam | 12 (7,17) | 8.8 (5.3,11.9) | 6572 (4939,8613) | 13 (9.8,17) | -37 (-57.3,-1.7) |
| Portugal | 5333 (4731,6031) | 53.1 (47.3,59.7) | 1739 (1149,2419) | 9.6 (6.3,13.4) | -30.5 (-40,-21.9) |
| Equatorial Guinea | 620 (410,895) | 123.8 (86.6,167.9) | 46 (33,68) | 42.1 (30.5,62.5) | -48.1 (-55.7,-39.8) |
| Maldives | 39 (26,56) | 18.5 (12.6,27.2) | 588 (328,1021) | 36.9 (21.3,60.8) | -55.8 (-72.5,-13) |
| Bulgaria | 6226 (5452,7165) | 72.3 (63.6,83.1) | 9088 (4186,15128) | 60.3 (29,99.4) | -40.6 (-49.6,-32.9) |
| Singapore | 564 (425,737) | 18 (13.5,23.5) | 27207 (13554,49567) | 61.7 (34.1,97.3) | -18.7 (-32.5,-5.9) |
| Seychelles | 16 (13,25) | 23 (19.1,35.2) | 11177 (2341,19876) | 7 (1.4,12.7) | -38.9 (-48.3,-21.6) |
| Jamaica | 462 (394,555) | 19.2 (16.3,23.1) | 24732 (15839,34062) | 156.5 (100.4,216.9) | -63.9 (-71.8,-56) |
| Haiti | 11366 (5577,18767) | 133.5 (77.4,204.8) | 6435 (3835,11904) | 51.8 (32.4,87.2) | -59.3 (-75.2,-21.1) |
| Eswatini | 883 (614,1410) | 106.4 (74.4,163.7) | 3226 (2206,5048) | 18.7 (12.9,29) | -43.8 (-53,-31.2) |
| Finland | 854 (668,1080) | 16.3 (12.7,20.8) | 63 (49,81) | 8.2 (6.3,10.5) | -61.4 (-69.8,-51.4) |
| El Salvador | 6896 (5940,8325) | 130 (113.7,155.3) | 10434 (4823,21022) | 84.1 (43.1,151.9) | -28.1 (-60.4,19.1) |
| Sudan | 34101 (12903,59857) | 123.4 (45.8,203.9) | 234 (179,346) | 4.5 (3.4,6.7) | -72.1 (-80.6,-58.9) |
| Mali | 10204 (5641,16047) | 84.5 (55.2,123) | 603 (387,873) | 13.5 (8.7,19.7) | -81.1 (-87,-73.7) |
| Tuvalu | 4 (2,6) | 38.4 (22.9,61.5) | 66862 (27006,96496) | 4.7 (1.9,6.9) | -31 (-59.7,21.1) |
| Georgia | 970 (758,1216) | 17.4 (13.6,21.7) | 0 (0,1) | 19.7 (13.1,34.5) | -12.2 (-21.8,-1.6) |
| Oman | 136 (97,203) | 6.6 (4.7,10) | 29348 (16295,57230) | 141.4 (81.8,266.2) | -72.4 (-82.5,-55.6) |
| Iceland | 53 (42,65) | 20.4 (16.1,25.3) | 15 (11,20) | 39.7 (30.1,56.2) | 26.8 (-30.4,97.6) |
| Antigua and Barbuda | 28 (25,31) | 46.5 (41.6,52.6) | 1233 (630,2224) | 28.4 (14.7,46.9) | -76.7 (-83.5,-63.3) |
| Kazakhstan | 6099 (5047,7305) | 36.6 (30.2,44.1) | 8572 (3737,15384) | 53.5 (25,91.1) | -5.3 (-41.6,49.2) |
| Latvia | 456 (345,603) | 17 (12.9,22.5) | 66 (47,100) | 2.2 (1.6,3.6) | -22.5 (-46.1,19.4) |
| Australia | 2993 (2337,3826) | 17.5 (13.6,22.3) | 91 (68,131) | 9.7 (7.3,14) | -73 (-78.8,-66.5) |
| United Kingdom | 16277 (12941,20125) | 27.5 (21.8,34.2) | 28 (20,37) | 6.9 (5,9.2) | -44.8 (-68,-7.8) |
| Sweden | 4995 (4400,5698) | 54.4 (47.5,62.5) | 1126 (790,1535) | 10.3 (7.2,14.2) | -46.5 (-54.3,-38) |
| Saint Vincent and the Grenadines | 56 (50,62) | 51.3 (46.4,56.6) | 882 (580,1232) | 9.4 (6.1,13.2) | -33 (-68.1,36.2) |
| Barbados | 126 (116,137) | 49.4 (45.5,53.8) | 1145 (837,1576) | 10.8 (7.9,15) | -65.2 (-73.4,-51.1) |
| Hungary | 5395 (4661,6291) | 49.7 (42.7,58) | 19715 (15784,24522) | 15 (12.1,18.5) | -32.1 (-38.6,-25.8) |
| Egypt | 22939 (16408,28505) | 34.5 (25.3,42.2) | 123 (85,169) | 8.7 (6,12) | -93 (-94.7,-91.2) |
| Niger | 11737 (5934,19213) | 96.5 (56,148.1) | 91 (68,157) | 16.2 (11.8,28.4) | -78.2 (-82,-74.1) |
| Czechia | 5410 (4418,6683) | 50.2 (41.2,62) | 362 (260,522) | 10.9 (7.8,15.7) | -71.9 (-82.7,-11.8) |
| Lithuania | 1230 (986,1597) | 33.4 (27,43.2) | 700 (464,989) | 13.2 (8.7,18.8) | -51.2 (-65.1,-25) |
| Honduras | 4714 (3824,5759) | 94.8 (78.1,114.3) | 236 (169,313) | 7.9 (5.7,10.6) | -53.5 (-64.9,-33.2) |
| Marshall Islands | 13 (7,21) | 28.9 (15.9,45.8) | 106 (72,152) | 16.2 (10.9,23.3) | -47.5 (-53.3,-42.6) |
| New Zealand | 684 (500,913) | 19.7 (14.4,26.2) | 36 (27,55) | 6.1 (4.7,8.7) | -35 (-40.5,-29.2) |
| Nicaragua | 2131 (1610,2621) | 50.6 (39.6,61.1) | 2830 (1748,5784) | 24.5 (15.1,51.9) | -92.9 (-95.3,-90.3) |
| Iraq | 3818 (2748,5291) | 17.4 (12.6,24.4) | 13 (10,18) | 74.2 (55.7,103) | -43.9 (-53.3,-35.4) |
| Germany | 11197 (8068,14947) | 13.4 (9.6,18) | 4414 (2965,6257) | 22.6 (15.2,32) | -76.4 (-82.2,-65.2) |
| Nauru | 3 (2,5) | 30.1 (17.8,45.1) | 6722 (3353,13279) | 43.5 (22.4,79.4) | -26 (-36.5,-16.5) |
| Syrian Arab Republic | 2217 (1421,3292) | 14.1 (9.4,21.7) | 3854 (3227,4501) | 21.2 (17.8,24.8) | -80 (-87.5,-67.2) |
| Pakistan | 10388 (6196,26842) | 7.9 (4.8,19.4) | 9 (6,13) | 9.7 (6.3,13.8) | -77.7 (-85.5,-64.2) |
| Uganda | 21463 (13456,32081) | 94.5 (64,134.7) | 2067 (1254,3514) | 44.1 (27.2,70.5) | -68.9 (-73.6,-63.8) |
| Trinidad and Tobago | 614 (570,665) | 50.5 (47,54.6) | 11321 (8558,14673) | 7.4 (5.7,9.5) | -28.9 (-49.9,8.1) |
| Ethiopia | 116725 (74622,165512) | 193.2 (131.3,258.5) | 6853 (4667,9375) | 10.7 (7.2,14.9) | -59.4 (-66.5,-52.1) |
| South Sudan | 9492 (5316,15032) | 129.4 (75.9,192.8) | 1665 (1096,2537) | 68.1 (45.1,101.5) | -37.1 (-58.2,-6.8) |
| Turkey | 8869 (6000,16738) | 14.6 (10.2,26.5) | 290 (229,381) | 5.1 (4,6.7) | -46.3 (-63.8,-10.4) |
| Djibouti | 371 (234,583) | 84.2 (55,126.1) | 31 (22,65) | 51.6 (35.2,107.9) | -83.9 (-87.1,-80.2) |
| Niue | 0 (0,1) | 21 (14.3,32.9) | 14118 (7773,25417) | 41.9 (24.7,68.9) | -68.2 (-78.6,-50.2) |
| Nigeria | 93908 (56158,169338) | 87.9 (58.8,137.9) | 11000 (6328,19984) | 62.9 (39.1,101.9) | -32.5 (-62.5,8.5) |
| Cameroon | 6865 (4465,9903) | 52.2 (36.2,72.1) | 388 (180,736) | 31.6 (15.1,57.6) | -69.9 (-77.1,-60.3) |
| Palau | 15 (9,23) | 95.6 (58.9,142.3) | 5323 (2311,9357) | 14.5 (6.3,25.3) | -43.7 (-63.9,-0.9) |
| Senegal | 6316 (3998,9415) | 63 (43.9,90.3) | 22980 (9699,40363) | 64.3 (25.9,108.5) | -83.7 (-87.6,-79.8) |
| Chad | 5794 (3222,9112) | 68.6 (45.3,106.6) | 24301 (13902,48553) | 79.1 (48.3,132.9) | -50 (-61.1,-36.6) |

**Figure S1**


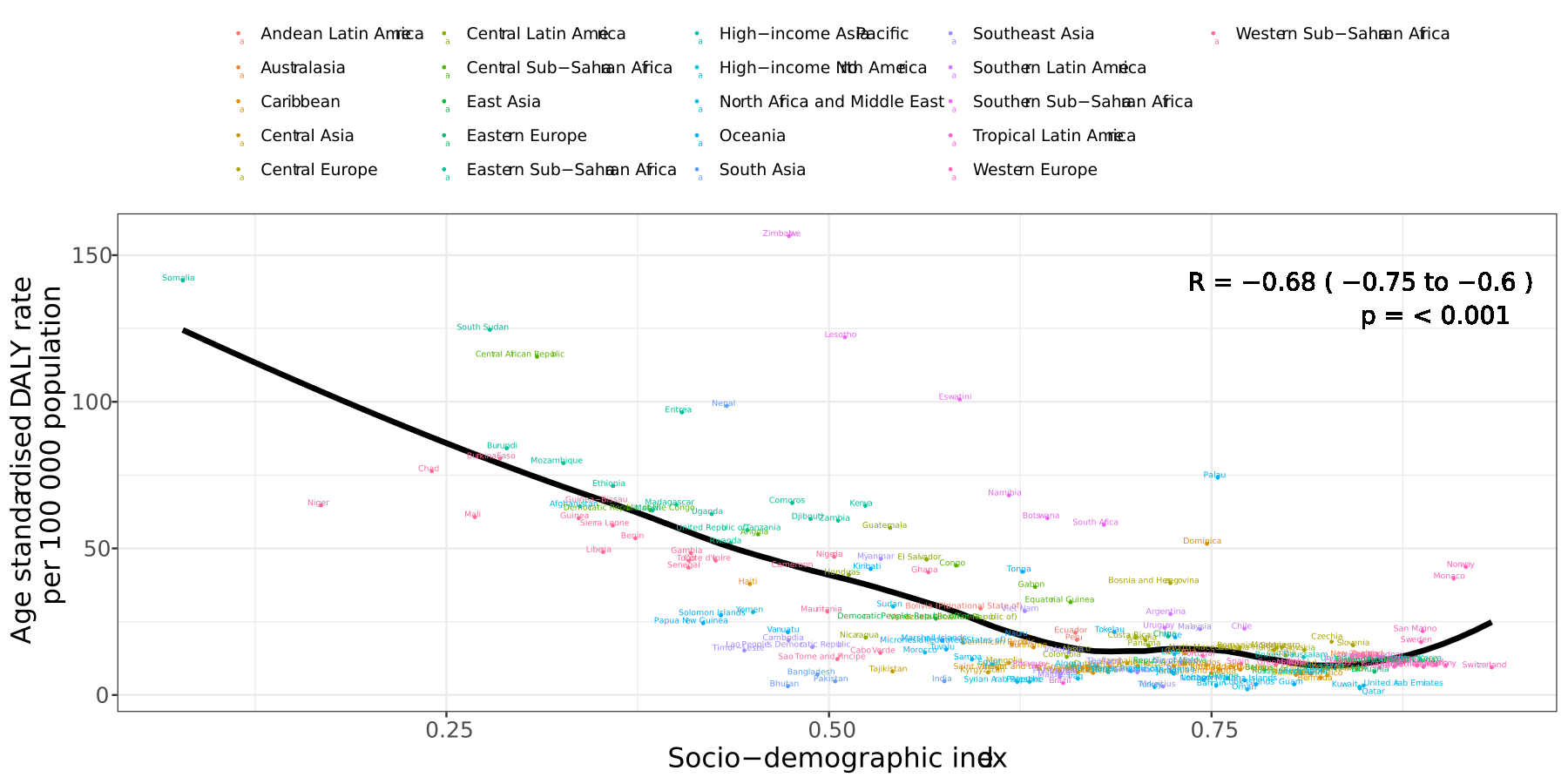

Supplement: SUPPLEMENTARY Table S1 — Prevalence of non-CO poisoning in 1990 and 2021 and the percentage change in the age-standardized prevalence rate (ASPR) per 100,000, by location. [file Table_1.docx]
